# Proximity to Natural Habitat Is Not Consistently Associated With Pollination Services in Tropical Smallholder Farms: A Systematic Review and Meta‐Analysis

**DOI:** 10.1111/ele.70229

**Published:** 2025-12-03

**Authors:** Ennia Bosshard, Mark E. Harrison, Frank van Veen, Chris J. Kettle, Nagaraja Badenahally Chikkarangappa, John E. Banks, Parthiba Basu, Quebin Bosbely Casiá‐Ajché, Bo Dalsgaard, Aditi Dutta, Eunice Enríquez, Natalia Escobedo‐Kenefic, Hugo Eduardo Fierros‐López, Barbara Gemmill‐Herren, Jaboury Ghazoul, Katrine Hansen, Annika L. Hass, Juliana Hipólito, Oliver Honnay, John Muo Kasina, Alexandra‐Maria Klein, Iris Kormann Motzke, Smitha Krishnan, Patricia Landaverde, Anderson Oliveira Latini, Kevin Li, Rodrigo Lucas‐García, Theodore Munyuli, Deepthi Narasimhaiah, Diana Obregon, J. Javier G. Quezada‐Euán, Mónica E. Riojas‐López, Victor Rosas‐Guerrero, Julian Schrader, Fernando Severiano‐Galeana, Tegegne Molla Sitotaw, Tuanjit Sritongchuay, Pornpimon Tangtorwangsakul, Manuel Toledo‐Hernandez, Teja Tscharntke, Poornima Viswanathan, Cassandra Vogel, Thomas C. Wanger, Kanuengnit Wayo, Catrin Westphal, Matt Lloyd Jones, Christopher N. Kaiser‐Bunbury

**Affiliations:** ^1^ Centre for Ecology and Conservation, Faculty of Environment, Science and Economy University of Exeter, Cornwall Campus Penryn UK; ^2^ Department of Earth and Environmental Science, Faculty of Environment, Science and Economy University of Exeter, Cornwall Campus Penryn UK; ^3^ Bioversity International Rome Italy; ^4^ Department of Environmental Science Bangalore University Bangalore India; ^5^ California State University, Monterey Bay Seaside California USA; ^6^ Department of Zoology University of Calcutta Kolkata India; ^7^ Centro de Estudios Conservacionistas Universidad de San Carlos de Guatemala Guatemala Guatemala; ^8^ Section for Molecular Ecology and Evolution, Globe Institute University of Copenhagen Copenhagen Denmark; ^9^ Departamento de Ecología, CUCBA Universidad de Guadalajara Zapopan Mexico; ^10^ World Agroforestry Centre Nairobi Kenya; ^11^ Ecosystem Management, Department of Environmental Systems Science ETH Zurich Zurich Switzerland; ^12^ Center for Macroecology, Evolution and Climate, Globe Institute University of Copenhagen Copenhagen Denmark; ^13^ Functional Agrobiodiversity & Agroecology, Department of Crop Sciences University of Göttingen Göttingen Germany; ^14^ Instituto Nacional da Mata Atlântica Santa Teresa ES Brazil; ^15^ Biology Department, Division of Ecology, Evolution and Biodiversity Conservation KU Leuven Leuven Belgium; ^16^ Kenya Agricultural and Livestock Research Organization Nairobi Kenya; ^17^ Chair of Nature Conservation and Landscape Ecology University of Freiburg Freiburg Germany; ^18^ Kormann & Marti GmbH, Eco‐Consulting Ins Switzerland; ^19^ Multifunctional Landscapes Bioversity International Bengaluru India; ^20^ Martin Luther University Halle‐Wittenberg General Zoology Institute of Biology Halle (Saale) Germany; ^21^ University of Guatemala Guatemala Guatemala; ^22^ Departamento de Ciências Exatas e Biológicas, Campus Sete Lagoas Universidade Federal de São João del‐Rei Sete Lagoas Brazil; ^23^ Department of Entomology Pennsylvania State University University Park Pennsylvania USA; ^24^ School for Environment and Sustainability University of Michigan Ann Arbor Michigan USA; ^25^ Posgrado en Recursos Naturales y Ecología, Facultad de Ecología Marina Universidad Autónoma de Guerrero Acapulco Guerrero México; ^26^ Escuela Superior en Desarrollo Sustentable Universidad Autónoma de Guerrero Tecpan de Galeana Guerrero México; ^27^ Department of Biology, Natural Resources & Environment, National Natural Sciences Research Center CRSN‐Lwiro Kavumu South‐Kivu Province DR Congo; ^28^ Department of Entomology Cornell University Ithaca New York USA; ^29^ Departamento de Apicultura Tropical Campus Ciencias Biológicasy Agropecuarias Universidad Autónoma de Yucatán Mérida Mexico; ^30^ School of Natural Sciences Macquarie University Sydney New South Wales Australia; ^31^ Department of Geography and Environmental Studies, Faculty of Social Sciences Bahir Dar University Bahir Dar Ethiopia; ^32^ Department of Environmental Science and Natural Resource Management Norwegian University of Life Sciences Ås Norway; ^33^ Samrongnuan, Muang Samutprakarn Thailand; ^34^ Instituto Tecnológico Vale (ITV) Belem Brazil; ^35^ Keystone Foundation The Nilgiris Tamil Nadu India; ^36^ Department of Ecology Swedish University of Agricultural Sciences Uppsala Sweden; ^37^ Production Technology and Cropping Systems Group, Department of Plant Production AgroScope Nyon Switzerland; ^38^ Academy of Global Food Economics and Policy China Agricultural University Beijing China; ^39^ Futuristic Science Research Center, School of Science Walailak University Nakhon Si Thammarat Thailand; ^40^ European Centre for the Environment and Human Health University of Exeter Medical School Penryn UK

**Keywords:** agroecosystems, bee, biodiversity conservation, ecosystem services, landscape ecology, pollinator diversity, sustainable agricultural landscapes, synthesis, tropical agriculture

## Abstract

Proximity to natural habitat is known to enhance pollination services in large‐scale agriculture, but it remains unclear whether this holds in tropical smallholder farms. These systems are embedded in ecologically complex landscapes, central to global food security, and depend heavily on biodiversity‐derived ecosystem services. We conducted a systematic review and meta‐analysis of 35 studies assessing the relationship between distance to natural habitat and pollinator abundance, species richness, and crop fruit set in tropical smallholder farms. We found no consistent patterns in pollinator abundance and crop fruit set with increasing distance, with relationships highly variable across studies. Similarly variable, yet slightly negative, was the relationship between distance and pollinator species richness. Our findings suggest limited support for the ‘proximity to natural habitat’ hypothesis in tropical smallholder farms, indicating that the inherent complexity of these landscapes may buffer negative effects of distance on pollination. This underscores the importance of maintaining and restoring landscape complexity to sustain biodiversity and ecosystem services such as crop pollination. We also highlight the need for greater methodological consistency and publicly available raw data in future studies to strengthen the evidence base and support management strategies for safeguarding pollination services in tropical smallholder farms.

## Graphical abstract

We conducted a systematic review and meta‐analysis of 35 studies to examine the relationship between distance to natural habitat and pollinator abundance, species richness and crop fruit set in tropical smallholder farms. We found no consistent effects on abundance or fruit set, and only a slight, highly variable negative effect on pollinator richness, suggesting limited support for the ‘proximity to natural habitat’ hypothesis. Our findings highlight the importance of maintaining and restoring landscape complexity to sustain pollinators and pollination services, while emphasising the need for greater methodological consistency and publicly available raw data in future studies.

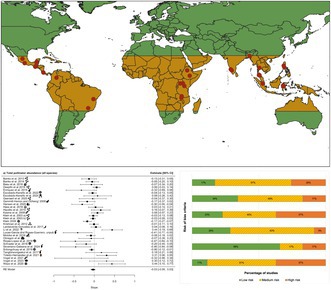

## 1 Introduction

Pollination plays a key role in supporting biodiversity and food production in agricultural landscapes (IPBES 2016), with an estimated 30% of the global food crop production volume depending on pollinators (Klein et al. 2007). Recognised as a key Nature's Contribution to People (IPBES 2019), pollination services underpin the availability of diverse and nutritionally balanced diets (Eilers et al. 2011; Smith et al. 2015; Gazzea et al. 2023), with many pollinator‐dependent crops relying on both managed and wild pollinators to boost yields (Osterman et al. 2021; Siopa et al. 2024). This is particularly important in smallholder farms, which account for 84% of the 570 million farms worldwide (Lowder et al. 2021) and constitute the primary means of livelihood for many of the world's most food insecure communities in the tropics (Laborde Debucquet et al. 2020; World Bank 2022). Smallholder farms are typically defined as family‐managed farms that produce crops or livestock on small plots (although definitions vary by region and context; FAO 2017) and have been found to support higher crop and non‐crop diversity compared to larger farms (Ricciardi et al. 2021). Maintaining and enhancing pollinator populations and diversity in tropical smallholder farms (TSFs) is thus a conservation and food security priority (United Nations 2015), especially amidst ongoing land‐use change, habitat fragmentation, and agriculture‐driven environmental degradation (Potts et al. 2010; Dicks et al. 2021).

One way of sustaining pollination services in TSFs is through pollinator‐friendly management and design strategies (IPBES 2016; Potts et al. 2016). Maintaining semi‐natural and natural habitat (hereafter ‘natural habitat’) near TSFs could be effective since these habitats provide essential resources for pollinators, such as food and nesting sites (Ricketts et al. 2008; Garibaldi et al. 2011; Cole et al. 2017; Tscharntke et al. 2021). Evidence from previous meta‐analyses supports this ‘proximity to natural habitat’ hypothesis, demonstrating that increasing distance to natural habitat negatively affects pollination (Ricketts et al. 2008; Garibaldi et al. 2011). However, these meta‐analyses included primarily data from studies on larger‐scale, more industrialised farms rather than TSFs. Furthermore, these meta‐analyses are now over a decade old and were not underpinned by a systematic review, meaning that relevant studies (old and new) may have been missed, and meta‐analytical results were not presented within the important context of quality appraisal of the included studies. Recent studies on the effects of isolation from natural habitat on pollination services in TSFs present an array of findings, ranging from declines (e.g., Silva et al. 2019; Obregon et al. 2021; Severiano‐Galeana et al. 2024) to no or even positive relationships (e.g., Bravo‐Monroy et al. 2015; Buchori et al. 2019; Toledo‐Hernandez et al. 2021). This raises the question of how consistent these patterns are in TSFs and what might explain the variability in findings across different studies.

We tested for the presence of a consistent negative relationship between distance to natural habitat and three proxies for pollination services: pollinator abundance, species richness and crop fruit set. We explored whether managed honeybees ( *Apis mellifera* Linnaeus, 1758 and/or *Apis cerana Fabricius, 1793* ) masked negative relationships between distance to natural habitat and the abundance of other pollinator species. Managed honeybees have been found to respond less to isolation from natural habitat compared to wild pollinators (Garibaldi et al. 2011), probably due to their larger foraging ranges, generalist feeding habits and capacity to colonise various habitats (Gathmann and Tscharntke 2002; Steffan‐Dewenter et al. 2002; Steffan‐Dewenter and Kuhn 2003; Scott Schneider et al. 2004; Osterman et al. 2021), as well as beekeeping practices such as artificial hives (Osterman et al. 2021) and supplementary feeding (Wakgari and Yigezu 2021).

The resources pollinators rely on, including nesting sites, nectar and pollen, can vary considerably among types of natural habitat (Eeraerts and Isaacs 2023), and many species depend on forest‐specific resources such as dead wood and floral resources from forest plants and tree resins (Ulyshen et al. 2023). We thus hypothesised that the relationship between distance to natural habitat and pollination services would be stronger for natural forests compared to other types of natural habitat. In addition, although TSFs generally exhibit lower‐input practices than large‐scale commercial farms, they can vary widely in management intensity. We hypothesised that decay relationships would be stronger in relatively high‐intensity TSFs, since extensive use of pesticides and other agrochemicals is strongly associated with pollinator declines (Millard et al. 2021), possibly making proximity to natural habitat more critical for maintaining pollination services. In contrast, low and intermediate levels of agricultural intensity can support diverse pollinator communities (Millard et al. 2021), and might thus sustain populations even at greater distances from natural habitat. Finally, we hypothesised that crops with higher pollinator dependence would show stronger fruit set declines with distance since reductions in pollination are likely to have greater yield impacts where biotic pollination is essential (Klein et al. 2007).

We investigated these hypotheses through a systematic review and meta‐analysis of studies of the relationship between isolation from natural habitat and pollination services in TSFs. This synthesis builds on and complements previous meta‐analyses (Ricketts et al. 2008; Garibaldi et al. 2011) by narrowing the geographical scope, which enabled us to underpin our meta‐analysis with a full systematic review conducted to current standards. This includes systematic searches for all relevant literature, critical quality appraisal of the included studies, and the application of more comprehensive and up‐to‐date meta‐analytical methods for the primary, subgroup and sensitivity analyses. These key features of a systematic review are designed to help reconcile the conflicting evidence for pollination distance‐decay relationships in TSFs, and thus inform more effective conservation and agricultural management strategies in these vital components of global food security and biodiversity.

## 2 Methods

### 2.1 Pre‐Registration, Guidelines and Reporting

The original systematic review and meta‐analysis plan was preregistered on the Open Science Framework in November 2022 (Bosshard et al. 2022). While the core hypotheses and overall methodological approach remain consistent with the preregistration, we have since implemented specific updates—particularly to the search strategy and statistical analysis—to align the systematic review with current best practices in evidence synthesis. All deviations from the original plan are reported in Table S3 following the standardised schema of Willroth and Atherton (2024). The systematic review and meta‐analysis was conducted and reported according to PRISMA (Page et al. 2021) and Cochrane Handbook guidelines (Higgins et al. 2024), to the extent possible for ecological studies.

### 2.2 Eligibility Criteria

Studies were considered eligible for inclusion based on the PECO (Population, Exposure/Comparator and Outcome) systematic review criteria (Morgan et al. 2018). Firstly, our Population criterion was that studies focused on insect pollinators and/or insect‐pollinated crops in TSFs. Farms were considered tropical if they were located between the Tropics of Cancer (23°27) and Capricorn (23°27). Given the lack of a universal definition of smallholder farming (Walpole et al. 2013; FAO 2017; Lowder et al. 2021), we applied a tiered classification approach to determine whether farms included in each study qualified as smallholder farms. We included studies explicitly describing farms as ‘smallholder’, ‘small‐scale’ or ‘subsistence’ farms, terms often used interchangeably (FAO 2017), and studies reporting farm sizes of ≤ 2 ha, a frequently adopted size threshold (FAO 2017). However, land size alone is often an insufficient criterion (Bukchin‐Peles 2025), and national thresholds vary widely. For example, 56% of 71 countries that use land size to define smallholder farms apply thresholds > 2 ha (GRAIN 2014; FAO 2017). We therefore also considered farms ≤ 15 ha as TSFs if they met at least two of the following characteristics: (a) low external inputs, (b) high crop diversity and/or (c) produce use mainly intended for household and local markets. These criteria were informed by existing literature (FAO 2017; Lowder et al. 2021) and adapted to reflect data availability, as detailed information on household income and labour allocation was often not accessible. When classification was uncertain, we also consulted corresponding authors to confirm whether their study sites met our smallholder farm criteria.

Secondly, our Exposure/Comparator criterion was that studies compared pollination variables in TSFs with varying degrees of isolation from natural habitat. We followed the definition of ‘natural habitat’ based on the classification used in the original studies, without imposing a standardised definition. What constituted natural habitat thus varied depending on how it was defined by the respective study authors (e.g., forests, shrublands or other semi‐natural areas). We included studies with different measures of isolation from natural habitat, including the proportion of natural habitat within a specific radius around the farm and categorical classifications such as ‘near’ and ‘far’, provided we were able to standardise these measures to distance to the nearest natural habitat (see data collection and data items section).

Thirdly, our outcome criterion was that studies assessed at least one of three proxies associated with insect pollination in smallholder farms: pollinator abundance (count of the number of individual pollinators), pollinator species richness (count of pollinator species) or fruit/seed set of pollinator crops (proportion of flowers that successfully developed into fruits or seeds).

Additionally, studies had to be empirical field studies, published in English, and report or make available upon request sufficient data to permit our meta‐analysis. A more detailed overview of our eligibility criteria is provided in Table S1.

### 2.3 Information Sources and Search Strategy

We identified relevant studies primarily by conducting systematic searches of three bibliographic databases: the Web of Science Core Collection, Scopus and CAB Abstracts. These were selected to identify peer‐reviewed reports of scientific studies (i.e., scientific papers) in ecology and agriculture. Web of Science and Scopus are widely recognised as core databases for ecological research (Foo et al. 2021), while CAB Abstracts is a dedicated database for agriculture and applied life sciences.

We searched the databases on 22 December 2024 using search terms related to (1) pollination services, particularly pollinator abundance, species richness, and/or fruit set; (2) agriculture and smallholder farms; (3) distance or isolation from natural habitat; and (4) the tropical biogeographic region. Boolean operators were used to combine terms, and the search strings were translated across information sources using PolyGlot (Clark et al. 2020). The complete search strategy is provided in Table S2.

In addition to the database searches, we screened all reports cited in three previously published meta‐analyses on this topic (i.e., Ricketts et al. 2008; Garibaldi et al. 2011; Moreaux et al. 2022), as well as studies and unpublished datasets that were recommended to us by colleagues and included those that met the above outlined eligibility criteria.

### 2.4 Selection Process

We conducted the study screening using Rayyan, a web‐based platform designed to facilitate systematic review screening (Ouzzani et al. 2016). Initially, duplicate records were removed using Rayyan's automated deduplication function. We then screened titles and abstracts to assess the relevance of the studies based on our eligibility criteria. We employed double‐blind screening, where EB and MLJ each screened all titles, abstracts, and full texts, and MEH triple screened 10% of the titles and abstracts. All screening was conducted independently, with reviewers blinded to each other's assessments. Discrepancies between reviewers were discussed and resolved through consensus. All studies that passed the initial abstract screening stage were subjected to full‐text review to confirm their eligibility based on the predefined eligibility criteria.

### 2.5 Data Collection Process and Data Items

We extracted data from all studies that met our eligibility criteria, focusing on distance to the nearest natural habitat (explanatory variable) in relation to pollinator abundance, species richness, and/or fruit set (response variables). Where possible, we accessed studies' raw data from open‐access repositories or directly from authors upon request, following an individual participant data style approach for our meta‐analysis (Tierney et al. 2024). This allowed us to reduce the variability associated with differing model specifications and co‐variates used to generate distance slopes (the effect size of interest) between studies. If raw data were not accessible, we extracted data from figures using the online graphical extraction tool ‘plotdigitiser’ where possible (Aydin and Yassikaya 2022). We also included relevant raw data from the online database compiled by Ricketts et al. (2008), who made their meta‐analysis data publicly available (NCEAS 2008). Studies for which we could not obtain at least one of the three response variables in relation to distance to natural habitat were excluded from the meta‐analysis, following our eligibility criteria. An overview of the variables and data extraction criteria is provided in the following sections. Outcomes regarding the inclusion and exclusion of studies are illustrated in the PRISMA diagram and detailed in the results section, alongside an overview of the characteristics of the included studies.

#### 2.5.1 Study Metadata

We extracted metadata for each dataset on the study design, location, number of sampling sites, crop species and/or flowering plant community observed and the description of the natural habitat following the definition in the original studies. We also recorded the focal pollinator taxa for each study, distinguishing between studies that considered all insect pollinators and those that focused on specific taxonomic groups (e.g., bees, Diptera or Arthropods). Taxonomic classifications were recorded as provided in the original studies, using the highest level of specificity reported.

#### 2.5.2 Pollination Variables

For each study, we extracted raw data relating to at least one of the three response variables: pollinator abundance, species richness, and/or fruit set (or seed set; collectively termed ‘fruit set’ herein). For the pollinator abundance and richness, we compiled count data of the number of individual pollinators (abundance) and pollinator species (richness) sampled within a specified time frame and area. We included studies that sampled pollinator abundance and richness with both active (such as timed observations of flower visitors either in plots or along transects, often by sweep netting) and passive methods (such as pan traps, sticky traps and glue traps). Where possible, we also extracted separate data for wild pollinator abundance, excluding the honeybees *Apis mellifera* and *Apis cerana* in areas where these were reported to be managed or where it was not possible to distinguish between wild and managed honeybees. We followed the definition of ‘pollinators’ provided in each primary study without further standardisation but accounted for inconsistencies or lack of clear definitions in our risk of bias assessment (see risk of bias section).

The term ‘fruit set’ in our synthesis refers to the proportion of crop flowers that successfully develop into fruits or seeds. This is considered the most direct indicator for pollination services among the three response variables as it captures the actual outcome of pollination, namely the successful fertilisation leading to fruit or seed production. Yet, it is also less frequently reported in the literature (e.g., Ricketts et al. 2008), and does not necessarily capture any insights on pollinator populations or diversity, as pollination services might be provided by a small minority of dominant species (Kleijn et al. 2015). Moreover, crop species vary widely in their levels of self‐compatibility and dependence on pollinators (Klein et al. 2007; Siopa et al. 2024), and fruit set can be influenced by various other factors such as soil nutrients, water availability and climate conditions (Bos et al. 2007). To ensure consistency across studies, we included only data on naturally occurring (open) fruit set, meaning fruit set measured under natural pollination conditions without experimental manipulation such as hand pollination or pollinator exclusions. We included both early‐stage and final fruit set data, depending on what was reported in each study, without distinguishing between the two.

#### 2.5.3 Distance Measures

We focused on the distance to the nearest natural habitat (in meters) as the explanatory variable for all three response variables. Some studies directly reported the distance of each sampling site from the nearest natural habitat, whereas others measured the proportion of natural habitat within the surrounding landscape in a specific radius or used distance categories such as ‘near’ and ‘far’ from natural habitat. Where possible, these indirect measures were converted to distance in meters using GPS locations provided by the corresponding authors to derive the distances from satellite imagery using the historical view in Google Earth Pro, estimated as close to the time of the study as possible (detailed description in Appendix 4 and Table S5). Otherwise, they were excluded from the systematic review and meta‐analysis as per the eligibility criteria because we could not derive the required data for the quantitative synthesis (Table S7).

#### 2.5.4 Other Potential Moderator Variables

To inform subgroup analyses (see below), we extracted data related to four variables hypothesised to be key moderators of the distance relationship: exclusion of managed honeybees in the pollinator counts, type of natural habitat, relative agricultural intensity and crop‐pollinator dependency for the fruit set response variable. Where managed honeybees ( *Apis mellifera* and in some cases *Apis cerana* ) were present or not distinguishable from wild honeybees in the study areas, we extracted separate data for the pollinator abundance variables with and without these species if this information was accessible from the published data or corresponding authors. We categorised natural habitat type based on the descriptions in the primary studies as either ‘natural forest’ or ‘other’, the latter including mixed, disturbed or non‐forest habitats such as agroforests, secondary vegetation, shrubland and wetlands. Agricultural intensity was coded as three categories: ‘high’ for studies on farms using synthetic pesticides and/or growing crops in monocultures; ‘low’ for study farms with no or very little agrochemical application and diverse crops; and ‘both’ where the study included sites with both relatively ‘high’ and ‘low’ agricultural intensity. Where primary studies did not report sufficient information to categorise the agricultural intensity of the farm sites, additional information was requested from the corresponding authors to confirm the categorization. Crop‐pollinator dependency levels for each crop within a study were assigned based on predefined quantitative values from a worldwide assessment of available pollination experiments (Siopa et al. 2024). We classified the continuous values ranging from 0 to 1 (zero representing lack of pollinator dependency and one representing the highest level) to the previously established following six levels of pollinator dependency by Klein et al. (2007): ‘essential’ (crop production reduction without pollinators ≥ 90%); ‘high’ (40− < 90% reduction); ‘modest’ (10− < 40% reduction); ‘little’ (> 0 and < 10% reduction); ‘none’ (0% reduction) and ‘unknown’ (no empirical studies available).

### 2.6 Study Risk of Bias Assessment

In systematic review, a risk of bias assessment (a type of critical appraisal) is used to evaluate the quality and reliability of included studies by identifying potential sources of methodological bias that could influence the overall findings (Moher et al. 2009; Stanhope and Weinstein 2023). We conducted study‐level risk of bias assessments using the Collaboration for Environmental Evidence's Critical Appraisal Tool (CEECAT) Version 0.3, a prototype that is currently one of the only available tools for environmental management research (Konno et al. 2021). The tool consists of six risks of bias criteria for observational studies: (1) confounding bias; (2) exposure selection bias; (3) misclassified comparison bias (incl. spatial autocorrelation); (4) detection bias; (5) outcome reporting bias; (6) outcome assessment bias. More details on the risk of bias assessment are provided in Appendix 7.

### 2.7 Effect Measures (Non Meta‐Analytical Models)

All effect measures for the meta‐analysis were calculated in R version 4.2.2 (R Core Team 2022). We estimated the slopes of distance to nearest natural habitat against each of the three pollination proxies (pollinator abundance, pollinator richness and fruit set) as the effect measures of our meta‐analyses. These effect measures estimate the expected change in pollination variables as distance to natural habitat increases. Slopes can be used directly as effect measures for meta‐analyses provided the slope in every study is measured in the same units (Rosenberg et al. 2013). This raw data approach allowed us to ensure standardised units across slopes for each of the three response variables, namely the count of pollinators (abundance), count of pollinator species (richness) and/or proportion of crop flowers setting fruit/seed (fruit set) per meter of increasing distance.

The datasets included in our meta‐analysis employed one of three broad study designs: (A) ‘single‐distance‐per‐site’, in which each site (e.g., farm) was sampled at a single distance to natural habitat, with distances varying between sites; (B) ‘nested distances’, in which multiple distances were sampled within a site; and (C) ‘paired sites’, in which sites were sampled in matched pairs that differed in proximity to natural habitat (more detail in Appendix 6). For designs B and C, we included site or pair identity as a random effect to account for non‐independence. Therefore, we estimated the exponential relationship via generalised linear models (GLMs; design A) or generalised linear mixed models (GLMMs; designs B and C) according to the following equation for each study individually in the first stage of our meta‐analysis, following the approach of Ricketts et al. (2008) and Garibaldi et al. (2011):

𝛾[𝑖𝑧]=α[𝑖]+𝛽[𝑖]𝐷[𝑖𝑧]+𝜀[𝑖𝑧]

where α[𝑖] and 𝛽[𝑖] are the intercept and slope of study 𝑖 respectively, 𝐷[𝑖𝑧] is the distance of site 𝑧 in study 𝑖 to the nearest natural habitat in meters, and 𝜀[𝑖𝑧] is the residual of site 𝑧 in study 𝑖. We used a negative binomial error distribution (with a log link function) for pollinator abundance and richness data as most studies showed overdispersion typical of count data (Lindén and Mäntyniemi 2011). We used a beta distribution (with a logit link function i.e., beta regression) for the fruit set data, which is well suited to the form in which this outcome is typically reported (without numerators and denominators; Mangiafico 2016). We used the ‘glm’ and ‘glm.nb’ functions from the MASS package (Venables and Ripley 2002) and ‘glmmTMB’ from the glmmTMB package (McGillycuddy et al. 2025) for the regression models. For datasets with multiple repeat measures but unbalanced sampling effort per site, we included the number of repeat measures per site as an offset in the pollinator abundance and richness models, and as weights in the fruit set beta regression models. As the explanatory variable (distance to the nearest natural habitat) was recorded at varying scales across the primary studies, we transformed the scales using the logarithm of the distance (log + 1) when fitting the models. We then extracted the estimated slopes and its standard error for each primary study for the meta‐analysis (Rosenberg et al. 2013), before quantitative synthesis via meta‐analysis.

### 2.8 Meta‐Analytical Synthesis Methods

#### 2.8.1 Primary Analyses

To estimate the overall effects across studies, we fitted separate meta‐analytical models for each of the three response variables, using the ‘rma’ function from the *metafor* package in R (Viechtbauer 2010). Effect sizes were represented by the estimated slopes from individual studies (see Section 2.7), with corresponding variances used as weights. A random‐effects model with restricted maximum likelihood (REML) estimation was applied to account for variation among studies, assuming that the true effect size differs across studies rather than being a single fixed value. We assessed the presence and strength of an effect using 95% confidence intervals (CIs) and *p*‐values, but did not rely solely on *p*‐value significance (e.g., *p* < 0.05) and interpreted effect sizes also in the context of their uncertainty, with narrower CIs indicating more precise estimates (Schünemann et al. 2023). Heterogeneity among the studies was assessed using the *Q* statistic and *I* ^2^ outputted from the ‘rma’ function. We used the following established rules of thumb when interpreting *I* ^2^ heterogeneity estimates: low, moderate and high for *I* ^2^ values of 25%, 50% and 75%, respectively (Higgins et al. 2003). We used the meta‐analytic effect size estimates to model the predicted decay and associated 95% confidence interval for all three pollination proxies with increasing distance to natural habitat, applying an exponential decay function based on the natural log of distance.

#### 2.8.2 Subgroup Analyses

To explore possible reasons for statistical heterogeneity, we conducted four subgroup analyses related to key hypotheses in the field. To test our hypothesis that the presence of managed honeybees may mask negative effects of distance to natural habitats on other pollinator species, we re‐ran our abundance model after excluding the honeybees *A. mellifera* as well as *A. cerana* in studies where the Asian honeybee was reported to be domesticated (Krishnan et al. 2012; Motzke et al. 2016; Schrader et al. 2018). The other two models were not rerun as for the species richness model, excluding only one or two species is unlikely to meaningfully affect the overall species richness patterns, and the fruit set model does not include a direct measure relating to pollinators. The results of subgroup models were then compared to those of the primary models in terms of the effect size estimate, statistical significance (*p*‐value), and direction of the distance relationship.

We performed meta‐regressions to investigate whether natural habitat type, agricultural intensity and crop pollinator dependency could moderate the effects of increasing distance to natural habitat on pollination variables. To test this, we extended the meta‐analytic models by adding single categorical moderators for agricultural intensity and crop pollinator dependency (more details on data items in Section 2.5.4) (Thompson and Higgins 2002).

#### 2.8.3 Sensitivity Analyses

We performed several sensitivity analyses to explore the robustness of our meta‐analytical results. To test whether the presence of outliers and influential cases may affect the validity and robustness of our meta‐analyses (Viechtbauer and Cheung 2010), we repeatedly fitted the model leaving out one study at a time using the ‘leave1out’ function provided in the *metafor* package (Viechtbauer 2010). We considered the results robust if excluding any individual study did not change the direction of the effect or shift the *p*‐value across the 0.05 threshold. We further conducted multiple sensitivity analyses to test for the potential effects of: (i) method of sampling pollinators (active vs. passive sampling methods); (ii) taxonomic level of species identification, i.e., (morpho)species‐ versus coarser levels such as family‐ or genus‐level (only for the species richness model); (iii) method of measuring distance to nearest natural habitat (reported vs. estimated); and (iv) spatial scale of maximum distances considered in each study (categorised as small < 750 m, medium 750–3000 m and large > 3000 m). Each moderator was included separately in the models to test its influence on effect size estimates for the meta‐analyses. More details on these sensitivity analyses are provided in Appendix 9 in the Supporting Information.

### 2.9 Publication Bias Assessment

We tested for publication bias (Egger et al. 1997), which can occur in meta‐analyses when certain research findings, such as non‐significant results, are less likely to be published, leading to a skewed representation of the available evidence (Nakagawa et al. 2022). We first generated funnel plots of the individual study effect sizes against their corresponding standard errors and evaluated the funnel plot asymmetry visually as an informal assessment of small study publication bias (Egger et al. 1997). However, as visual interpretation alone is subjective (Tang and Liu 2000), we also used the more formal method of Egger's regression, where a non‐significant result suggests no strong evidence of publication bias (Egger et al. 1997).

### 2.10 Certainty Assessment

Finally, we conducted a certainty assessment, which is a standardised way to bring together the results of a systematic review and meta‐analysis by evaluating the strength of the available evidence, considering factors that may influence confidence in the results (Schünemann et al. 2023). We used the GRADE (Grading of Recommendations, Assessment, Development and Evaluations) approach, following the Cochrane guidelines as far as possible in the context of our ecology‐focused meta‐analysis (Schünemann et al. 2023). Certainty was categorised as high, moderate, low or very low, based on five domains for potential downgrading: (i) risk of bias, assessed using CEECAT to evaluate the potential for bias in individual studies; (ii) inconsistency, assessed via visual inspection of heterogeneity in the forest plots and via the *I* ^2^ statistic; (iii) indirectness, considering whether the available evidence directly answers our research question or if there are differences in study populations, exposure measures (distance to natural habitat) and pollination response variables that make the evidence less applicable; (iv) imprecision, based on confidence interval width and sample size adequacy; and (v) publication bias, assessed using funnel plots and Egger's regression test. As our systematic review and meta‐analysis focused on observational studies, the initial certainty of evidence was set as low. Each of the five domains could further downgrade or, in some cases, upgrade the certainty by one or more levels (Schünemann et al. 2023). Final certainty ratings were summarised in a ‘Summary of Findings’ table, following GRADEpro GDT recommendations.

## 3 Results

### 3.1 Study Selection

**FIGURE 1:**
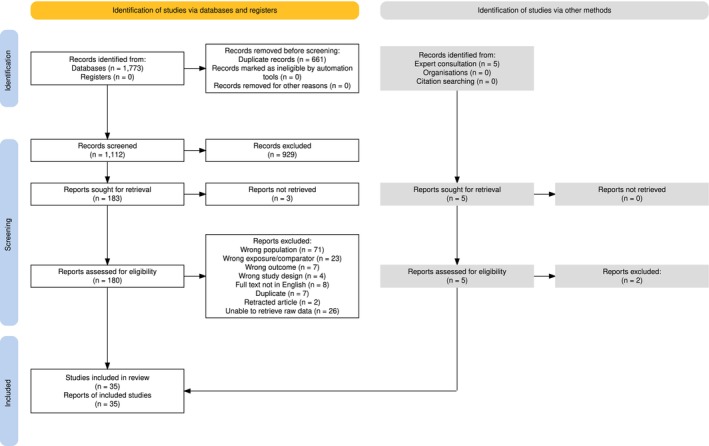
PRISMA flow diagram, created using the PRISMA flow diagram tool (Haddaway et al. 2022).

Our database searches returned a total of 1773 records, which were reduced to 1112 unique records after removing 661 duplicates. During title and abstract screening, 929 records were excluded based on our eligibility criteria, leaving 183 records for which we sought full‐text reports and subsequently 180 records for which we were able to obtain full texts. We excluded 148 of these based on our eligibility criteria (Table S5) and thus were able to obtain data of 32 reports identified through the systematic literature review. Corresponding authors from whom we requested data also directed us towards other potentially relevant datasets, resulting in the inclusion of three additional reports. The final dataset therefore included raw data from 35 reports (i.e., journal articles and unpublished manuscripts). The full selection process is summarised in the PRISMA flow diagram (Figure 1).

### 3.2 Study Characteristics

**FIGURE 2:**
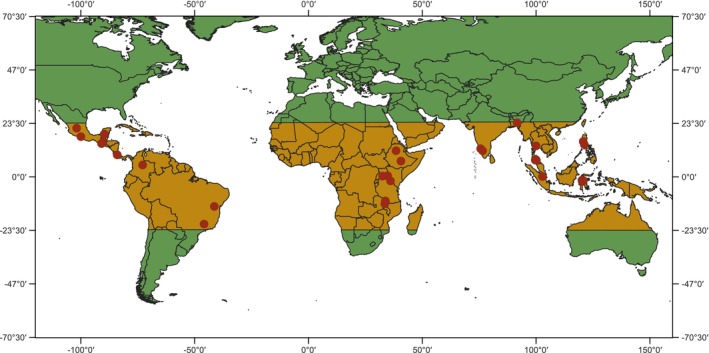
Map showing the geographic distribution of the 35 studies included in the meta‐analysis, represented by red dots. Note that there is spatial overlap of studies conducted in the same or nearby regions. The tropics are indicated in orange. Map was created in QGIS.

The 35 datasets included in the meta‐analysis covered over 500 farms across 13 tropical countries, with 31 studies reporting pollinator abundance, 30 studies reporting species richness and 17 studies reporting fruit set in relation to isolation from natural habitat (Figure 2, Table 1). Almost half of the studies were from Asia (16 studies; 46%), followed by the Americas (12 studies; 34%) and Africa (7 studies; 20%). The majority of studies employed a single‐distance‐per‐site design (27), six studies used nested distances within sites and two studies applied a paired‐site design. Of the 35 studies, 24 directly reported distances to the nearest natural habitat, while distance had to be calculated using satellite imagery for the remaining 11 studies. In total, distances for 21 studies (60%) were measured exclusively to the nearest natural forest, while distances for 14 studies (40%) focused on other or mixed types of natural habitat such as agroforests and shrublands. The studies covered a broad range of spatial scales, with maximum distances considered in studies ranging from 60 to 10,000 m (median = 700 m, mean = 1880 m). A total of 15 studies were carried out on low‐intensity farms that used very little or no agrochemicals and employed diverse cropping systems, 13 on relatively high‐intensity farms with chemical pesticide use or monocultures, five spanned both intensities, and two lacked sufficient data to classify.

**TABLE 1:** Characteristics of the 35 datasets on pollination variables in TSFs included in our analysis, with information on study location, number of study sites, crop species and pollinator dependency, focal pollinator taxa, agricultural intensity, a description of the semi‐natural habitat (SNH), reported distance measure, spatial scales and estimated slopes for the three pollination variables: a = pollinator abundance, r = pollinator richness, f = fruit set.

Authors and year	Country	Study sites	Study design		Crop species	Pollinator dependence^a^	Focal pollinator taxa	Agri. intensity^b^	SNH	Distance measure^c^	Spatial scale	Estimated slopes		
												a	r	f
Banks et al. (2013)	Costa Rica	12	Single distance per site	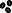	Coffee (arabica)	0.31	Hymenoptera	High	Forest, min. 0.5 ha size	Estimated	0–300 m	−0.13	0.02	—
Banks et al. (2014)	Costa Rica	12	Single distance per site	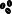	Coffee (arabica)	0.31	Hymenoptera	High	Forest, min. 0.5 ha size	Estimated	0–300 m	−0.05	—	—
Basu et al. (2016)	India	12	Single distance per site	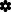	General community	NA	Bees	Both	Fallow	Reported	4–500 m	−0.07	0.02	—
Deepthi et al. (2019)	India	10	Nested distances	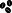	Coffee (canephora)	0.63	Bees	High	Riparian forest	Reported	10–60 m	0.06	0.03	—
Enríquez et al. (2015)	Guatemala	10	Single distance per site	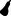	Squash	1	Bees	Low	Secondary vegetation	Reported	12–240 m	−0.32	−0.02	0.83
Escobedo‐Kenefic et al. (2022)	Guatemala	8	Single distance per site	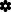	General community	NA	Insecta	High	Forest and semi‐natural vegetation	Estimated	0–750	−0.04	0.01	—
Escobedo‐Kenefic et al. (2024)	Guatemala	22	Nested distances	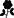	*Brassica rapa*	0.39	Insecta	Both	Humid montane and low‐montane forest	Reported	3–700 m	0.08	—	−0.01
Geeraert et al. (2020)	Ethiopia	18	Single distance per site	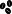	Coffee (arabica)	0.31	Bees	Low	Coffee production forest	Estimated	40–400 m	−0.04	−0.20	−0.26
Gemmill‐Herren and Ochieng (2008)	Kenya	5	Nested distances	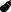	Eggplant	0.83	Bees	High	Forest	Reported	0–150 m	−0.17	−0.00	—
Hansen et al. (2020)	Thailand	6	Single distance per site	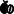	Guava	0.08	Insecta	High	Evergreen forest	Reported	200–1700 m	−0.30	−0.34	0.24
Hass et al. (2018)	Philippines	16	Single distance per site	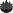	Rice	NA	Bees	High	Agroforests	Reported	0–2,55 m	0.03	−0.14	—
Hipólito et al. (2018)	Brazil	19	Single distance per site	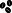	Coffee (arabica)	0.31	Insecta	Both	Natural vegetation	Reported	37–865 m	0.16	−0.09	0.15
Kasina et al. (2009)	Kenya	28	Single distance per site	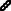	Dry common bean	0.19	Bees	Low	Rainforest	Reported	0–8000 m	0.08	−0.00	—
Klein et al. (2003a)	Indonesia	24	Single distance per site	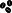	coffee (arabica)	0.31	Bees	Low	Rainforest	Reported	0–2500 m	−0.03	−0.10	−0.11
Klein et al. (2003b)	Indonesia	15	Single distance per site	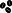	Coffee (canephora)	0.63	Bees	Low	Rainforest	Reported	0–1500 m	−0.03	−0.05	−0.18
Klein et al. (2009)	Indonesia	24	Single distance per site	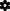	General community	NA	Bees	Low	Rainforest	Reported	0–1415 m	−0.17	−0.14	—
Krishnan et al. (2012)	India	35	Nested distances	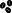	Coffee (canephora)	0.63	Bees	NA	Forest fragments (0.3–20 ha)	Reported	0–500 m	−0.01	−0.02	0.01
Landaverde‐Gonzalez et al. (2017)	Mexico	37	Single distance per site	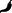	Chilli	0.48	Bees	High	Forest, woody vegetation, pastures	Estimated	0–600 m	0.04	−0.04	−0.14
Latini et al. (2020)	Brazil	8	Nested distances	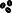	Coffee (arabica)	0.31	NA	Both	Atlantic Forest Remnants	Reported	0–120 m	—	—	−0.00
Li et al. (2022)	Indonesia	1	Single distance per site	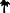	Oil palm	0.81	Arthropods	High	dipterocarp forest	Reported	0–100 m	0.44	0.04	−0.22
Lucas‐García et al. (2021 and 2025)	Mexico	18	Single distance per site	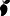	Mango	0.71	Insecta	High	Forest	Reported	50–1100 m	−0.41	−0.14	−0.39
Motzke et al. (2016)	Indonesia	13	Single distance per site	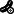	Cucumber	0.56	Bees	NA	Rainforest	Reported	1–2300 m	−0.08	—	—
Munyuli (2012)	Uganda	16	Single distance per site	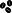	Coffee (canephora)	0.63	Bees	Low	Forest, wetland	Reported	5–7000 m	—	—	−0.31
Obregon et al. (2021)	Colombia	10	Single distance per site	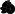	‘lulo’ (or ‘naranjilla’)	1	Bees	High	Primary/secondary forest	Estimated	0–90 m	−0.07	−0.02	0.05
Riojas‐Lopez et al. (2019)	Mexico	8	Single distance per site	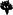	Nopal	NA	Bees	Low	Remnants of shrubland	Reported	100–870 m	−0.47	−0.04	—
Schrader et al. (2018)	Philippines	18	Paired sites	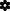	General community	NA	Bees	Low	Woody habitat	Estimated	0–90 m	−0.20	−0.13	—
Severiano‐Galeana et al. (2024)	Mexico	24	Single distance per site	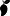	Mango	0.71	Insecta	High	Tropical dry forest patches	Reported	50–200 m	−0.46	−0.20	−0.29
Sitotaw et al. (2022)	Ethiopia	18	Nested distances	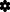	Mango, coffee (arabica), horse bean and field pea	0.71; 0.31; 0.05; NA	Insecta	Low	Sacred church forest	Reported	1–5000 m	—	−0.23	—
Sritongchuay et al. (2019)	Thailand	20	Paired sites	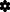	General community	NA	Insecta	Low	Rainforest (360–65,000 ha)	Estimated	500–8000 m	−0.06	0.26	—
Tangtorwongsakul et al. (2018)	Thailand	24	Single distance per site	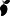	Mango	0.71	Bees	High	Mangrove forest, wetlands	Estimated	100–5500 m	0.18	0.08	—
Toledo‐Hernandez et al. (2021)	Indonesia	18	Single distance per site	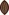	Cocoa	1	Diptera	Low	Secondary forest patches and cocoa agroforests	Reported	100–3200 m	0.31	0.17	—
Viswanathan et al. (2020)	India	7	Single distance per site	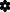	General community	NA	Insecta	Both	Forest reserve	Reported	100–2200 m	—	−0.31	—
Vogel et al. (2021)	Malawi	9	Single distance per site	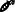	Pigeon pea	0.17	Bees	Low	Shrubland and forest	Estimated	10–250 m	−0.32	0.15	0.30
Vogel et al. (2023)	Malawi	24	Single distance per site	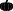	Pumpkin	1	Insecta	Low	Shrubland	Estimated	5–200 m	0.30	−0.02	−0.33
Wayo et al. (2020)	Thailand	30	Single distance per site	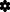	General community	NA	Stingless bees	Low	Rainforests and fragmented patches	Reported	0–10,000 m	0.44	−0.28	—

^a^ Crop pollinator dependence levels from Siopa et al. (2024).

^b^ Agricultural intensity of the study sites was categorised into ‘high’, ‘low’ or ‘both’ if the study contained both sites with high and low agricultural intensity.

^c^ The distance measure of primary studies was classified as either ‘reported’ (distances directly provided in the original research) or ‘estimated’ (distances derived from satellite imagery). More details on these variables can be found in the methods section (data items).

### 3.3 Risk of Bias Assessment

**FIGURE 3:**
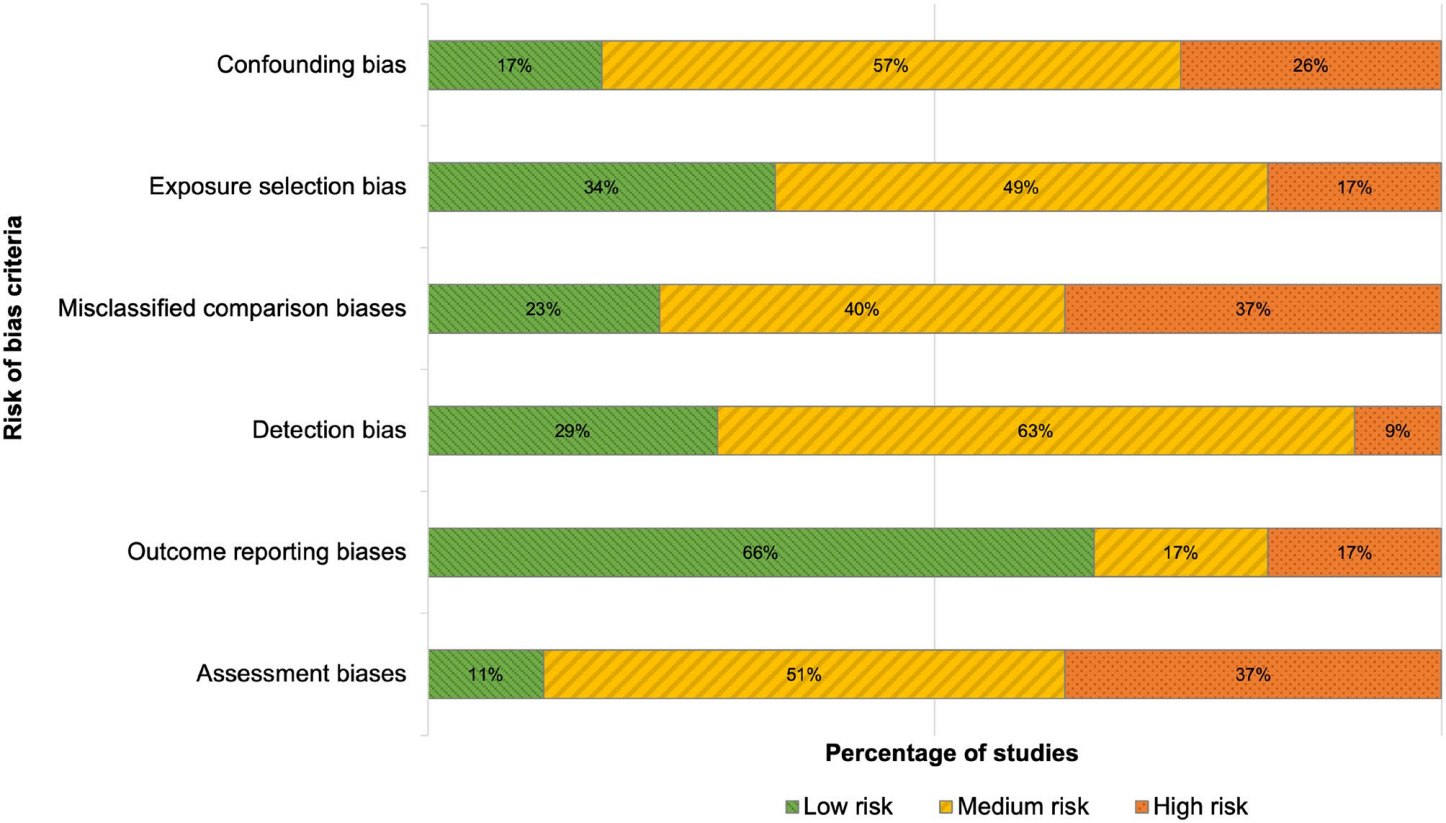
Outcome of the risk of bias assessment across the studies included in the meta‐analysis (*N* = 35).

As expected in observational field studies, all studies included in the systematic review and meta‐analysis exhibited a medium to high risk in at least one of the six domains assessed, particularly concerning risk of confounding bias, misclassified comparison bias and outcome assessment bias (Figure 3; Appendix 7 in the Supporting Information). Most studies did not account for all key confounding factors, such as the influence of agrochemical application or the size and quality of natural habitat. Furthermore, several studies lacked explicit justifications for spatial scales used to define isolation from natural habitat and minimum distances between study sites, making it difficult to assess potential non‐independence. Additionally, many studies had small sample sizes, which contributed to underpowered analyses and increased the risk of outcome assessment bias. However, the risk of bias assessment should be interpreted with caution, as its criteria were not fully adaptable to the heterogeneous ecological contexts and inevitably involved subjective judgement, such that these results provide qualitative rather than definitive insights into study quality.

### 3.4 Patterns in Pollinator Abundance With Increasing Distance to Natural Habitat

**FIGURE 4:**
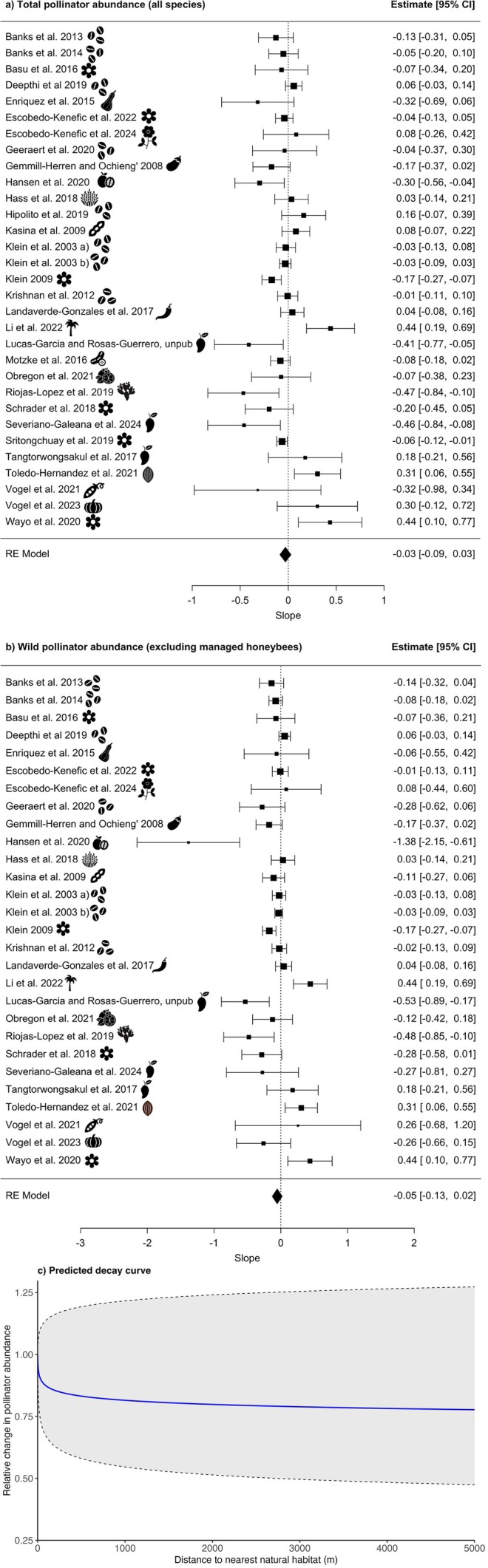
Relationship between pollinator abundance and distance to the nearest natural habitat: (a) forest plots showing the slopes (effect sizes) and 95% confidence intervals (CI) of pollinator abundance for all species (*N* = 31 studies); and (b) a subgroup analysis of the abundance of only wild pollinators (excluding managed honeybees; *N* = 28 studies). An icon representing the crop for each study is shown, for the crop names relating to symbols, please see Table 1. The size of each square is proportional to the weight of the study in the meta‐analysis, which is based on the inverse variance of its estimate. The dotted line represents a null effect (slope = 0); studies whose confidence intervals do not overlap this line indicate a statistically significant relationship between pollinator abundance and distance to the nearest natural habitat. (c) the decay curve showing the expected relative change in pollinator abundance (all species) with increasing distance to natural habitat, based on the estimated slope (−0.03) and 95% CIs (−0.09 to 0.03) from the meta‐analysis. The solid blue line represents the mean predicted abundance, while the shaded region and dashed lines indicate the 95% CI.

Effect measures representing the relationship between distance to the nearest natural habitat and pollinator abundance were calculable for 31 studies. The maximum distance to the nearest natural habitat varied across studies, ranging from 60 to 9937 m, with a mean of 2026 m and a median of 661 m. An overview of the individual data and model fits for the relationship between pollinator abundance and distance to the nearest natural habitat of each study can be found in Figure S3, and the study‐level effect sizes are presented in Table S8. When meta‐analytically aggregating the effect sizes from these models, there was no evidence for a consistent relationship between distance and pollinator abundance (slope: −0.03, 95% CI: −0.09 to 0.03, *p* = 0.32; Figure 4a, Table S9). Based on the slope of the meta‐analysis, the predicted decline in abundance at 1 km distance to natural habitat was 19%. Many studies had effect sizes close to zero, and high heterogeneity was observed between the studies (*I* ^2^ = 74.70%, *τ* ^2^ = 0.02, *τ* = 0.12, *Q* (df = 30) = 80.62, *p* < 0.0001; Table S10).

A subgroup analysis of the 28 studies for which we were able to restrict pollinator abundance to wild pollinators (i.e., exclude managed honeybees; study‐level effect sizes in Table S11) did not detect an effect either (slope: −0.05, 95% CI: −0.13 to 0.02, *p* = 0.16; Figure 4b). Similarly, we did not detect significant moderation of the effect by natural habitat type (QM (df = 2) = 1.32, *p* = 0.52; Tables S9 and S10) or relative agricultural intensity (QM (df = 3) = 1.35, *p* = 0.72; Tables S9 and S10). Residual heterogeneity remained high for both moderator analyses (natural habitat type: *I* ^2^ = 75.29%, *τ* ^2^ = 0.02, *τ* = 0.13, QE (df = 29) = 78.98, *p* < 0.001 and agricultural intensity: *I* ^2^ = 80.32%, *τ* ^2^ = 0.02, *τ* = 0.15, QE (df = 26) = 76.35, *p* < 0.001), suggesting substantial unaccounted variability across studies.

### 3.5 Patterns in Pollinator Species Richness With Increasing Distance to Natural Habitat

**FIGURE 5:**
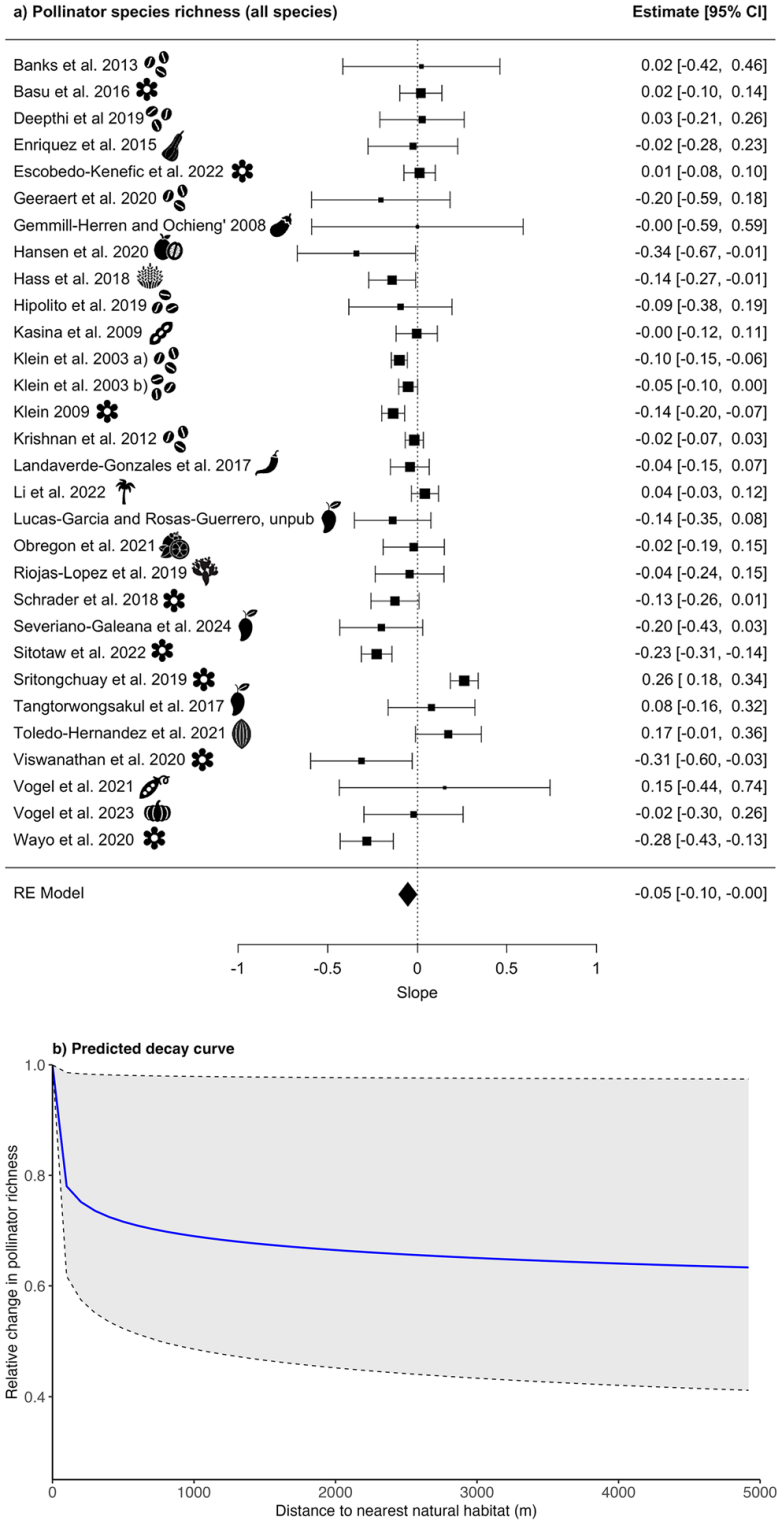
Relationship between pollinator species richness and distance to the nearest natural habitat: (a) forest plots showing the slopes (effect sizes) and 95 confidence intervals (CI) for the total pollinator species richness (*N* = 30studies). An icon representing the crop for each study is shown, for the crop names relating to symbols, please see Table 1. The size of each square is proportional to the weight of the study in the meta‐analysis, which is based on the inverse variance of its estimate. The dotted line represents a null effect (slope = 0); studies whose confidence intervals do not overlap this line indicate a statistically significant relationship between pollinator richness and distance to the nearest natural habitat; and (b) the decay curve of the predicted pollinator richness with increasing distance to natural habitat, based on the estimated slope (−0.05) and 95% CIs (−0.10 to −0.00) from the meta‐analysis. The solid blue line represents the mean predicted species richness, while the shaded region and dashed lines indicate the 95% CI.

Effect measures representing the relationship between distance to the nearest natural habitat and pollinator richness were calculable for 30 studies (see Figure S4, Table S12). Variation in the maximum distance to natural habitat across studies ranged from 60 to 9937 m, with a mean of 2112 m and a median of 808 m. When meta‐analytically aggregating the effect measures from these models, we detected a significant negative relationship between distance to the nearest natural habitat was associated with and pollinator richness (slope: −0.05, 95% CI: −0.10 to −0.00, *p* = 0.04; Figure 5a, Table S13). The predicted decline in species richness at 1 km distance to natural habitat was 31%. Similar to abundance, there was high heterogeneity in slope estimates between studies (*I* ^2^ = 79.49%, *τ* ^2^ = 0.01, *τ* = 0.11, Q (df = 29) = 131.35, *p* < 0.0001; Table S14).

We did not detect moderation of the effect by relative agricultural intensity (QM (df = 3) = 4.18, *p* = 0.24; Tables S13–S14) or habitat type (QM (df = 2) = 5.01, *p* = 0.08; Tables S13–S14), and residual heterogeneity remained high in both models (agricultural intensity: *I* ^2^ = 79.63%, *τ* ^2^ = 0.01, *τ* = 0.12, QE (df = 26) = 126.45, *p* < 0.001; habitat type: *I* ^2^ = 79.90%, *τ* ^2^ = 0.01, *τ* = 0.11, QE (df = 28) = 130.11, *p* < 0.001).

### 3.6 Patterns in Fruit Set With Increasing Distance to Natural Habitat

**FIGURE 6:**
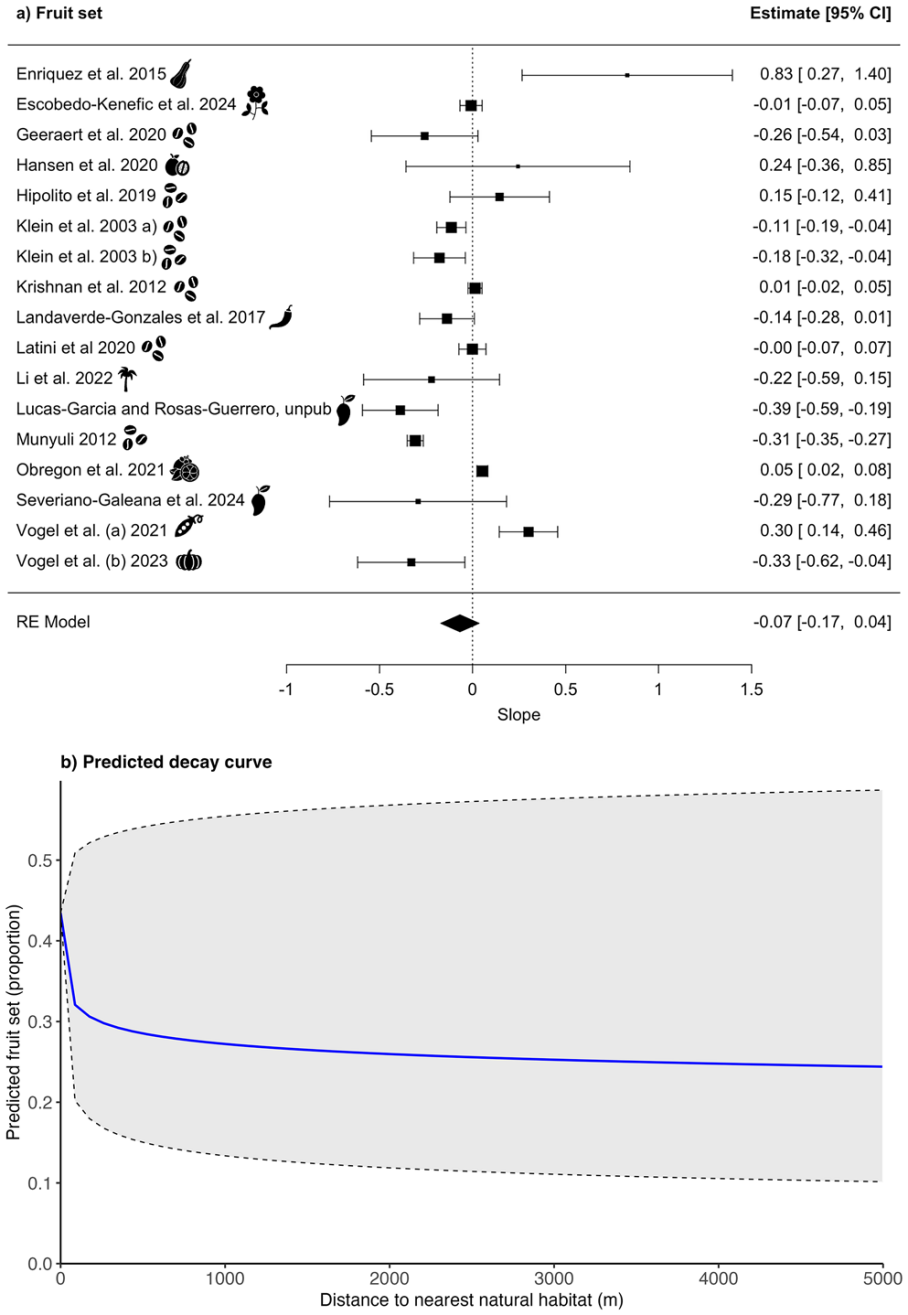
Relationship between fruit set and distance to the nearest natural habitat: (a) forest plot showing the slopes (effect sizes; *N* = 17 studies). An icon representing the crop for each study is shown, for the crop names relating to symbols, please see Table 1. The size of each square is proportional to the weight of the study in the meta‐analysis, which is based on the inverse variance of its estimate. The dotted line represents a null effect (slope = 0); studies whose confidence intervals do not overlap this line indicate a statistically significant relationship between pollinator abundance and distance to the nearest natural habitat. (b) the decay curve of fruit set with increasing distance to natural habitat based on estimated effect size, CIs, and proportion of fruit set at 0 m (slope = −0.07, CI = −0.17 to 0.04). The solid blue line represents the mean predicted abundance, while the shaded region and dashed lines indicate the 95% CI.

Effect measures representing the relationship between distance to the nearest natural habitat and fruit set were calculable for 17 studies (see Figure S5, Table S15). Across the studies, the maximum distances to natural habitat ranged from 90 to 8676 m, with a mean of 1557 m and a median of 500 m. When meta‐analytically aggregating the effect measures from these models, we detected no effect of distance to natural habitat (slope: −0.07, 95% CI: −0.17 to 0.04, *p* = 0.20; Figure 6, Table S16), and high heterogeneity across all studies (*I* ^2^ = 95.48%, *τ* ^2^ = 0.03, *τ* = 0.19, *Q* (df = 16) = 255.30, *p* < 0.0001, Table S17). Based on the slope from the meta‐analysis, the predicted decline in fruit set at 1 km distance to natural habitat was 37%.

In subgroup analyses, we did not detect any moderation of the effect by natural habitat type (QM (df = 2) = 1.66, *p* = 0.44; Table S17), agricultural intensity (QM (df = 3) = 2.57, *p* = 0.46; Table S17) or level of pollinator dependency of the target crop species (QM (df = 4) = 6.40, *p* = 0.17; Table S17).

### 3.7 Sensitivity Analyses

The sensitivity analyses revealed one influential study for the pollinator richness model (Sritongchuay et al. 2019) (Figure S8), and no influential studies for the abundance and fruit set models (Figures S6 and S10). Leave‐one‐out analysis indicated that excluding this study did not alter the overall direction or qualitative conclusion of the species richness meta‐analysis, instead slightly strengthening the negative pooled effect estimate and reducing heterogeneity (Table S19). For all three response variables (pollinator abundance, species richness, and fruit set), the Egger's regression test indicated no significant asymmetry (abundance: *p* = 0.62; richness: *p* = 0.57; fruit set: *p* = 0.47), and none of the funnel plots indicated clear asymmetry (Figures S7, S9, and S11). Thus, we found no evidence of publication bias.

Moderator analyses examining the effects of pollinator sampling methods (‘active’, ‘passive’ and ‘combined’) showed no significant effect on pollinator abundance (QM (df = 3) = 4.91, *p* = 0.18; Tables S9 and S10) or species richness (QM (df = 3) = 7.11, *p* = 0.07; Tables S13 and S14). In the species richness model, taxonomic resolution of species identification did not moderate the relationship between species richness and distance to natural habitat (QM (df = 2) = 4.53, *p* = 0.10; Tables S13 and S14). Moderator analyses on distance method showed that species richness declined more sharply when distances were reported rather than estimated (QM (df = 2) = 8.67, *p* = 0.01; slope: −0.08, CI: −0.14 to −0.03, *p* = 0.004; Tables S13 and S14), while no effects were found on abundance (QM (df = 2) = 0.99, *p* = 0.61; Tables S9 and S10) and fruit set (QM (df = 2) = 1.60, *p* = 0.45; Tables S16 and S17). We found a stronger negative effect for studies with a maximum distance between 750 and 3000 m (medium spatial scales) for pollinator richness (QM (df = 3) = 9.67, *p* = 0.02; slope: −0.12, CI: −0.19 to −0.04, *p* = 0.003; Tables S13 and S14) but not for pollinator abundance (QM (df = 3) = 5.24, *p* = 0.16; Tables S9 and S10) or fruit set (QM (df = 3) = 4.81, *p* = 0.19; Tables S16 and S17).

### 3.8 Certainty Assessment

The certainty of evidence was rated as very low for all three response variables, primarily due to concerns with study risk of bias, high heterogeneity among studies, indirectness of pollination proxies, and imprecision from small sample sizes in the fruit set meta‐analysis. Table 2 summarises the key results alongside their certainty ratings and plain language interpretations (see Table S21 for a more detailed description of the GRADE assessment).

**TABLE 2:** Summary of findings on the relationship between distance to natural habitat and pollination in TSFs, following Cochrane recommendations (Schünemann et al. 2023). The four symbols represent levels of certainty (very low, low, moderate and high), with a ‘⊕’ indicating retained certainty and a ‘⊝’ indicating a downgrade in certainty level.

Response variables	No of studies	Relative effect (95% CI) (distance to natural habitat)	Certainty of the evidence (GRADE)	Plain language interpretation
Pollinator abundance (count of pollinators)	Based on 701 data points from 31 studies	Slope: −0.03 (−0.09 to 0.03) *p* = 0.32 Distance range 0–9937 m (maximum distance median = 661 m, mean = 2026 m)	⊕⊝⊝⊝ very low^a^, ^b^, ^c^, ^d^	Increasing distance to natural habitat may not affect pollinator abundance in TSF, but there is low certainty in this conclusion.
Pollinator richness (count of unique pollinator species)	Based on 731 data points from 30 studies	Slope: −0.05 (−0.10 to −0.00) *p* = 0.04 Distance range 0–9937 m (maximum distance median = 808 m, mean = 2112 m)	⊕⊝⊝⊝ very low^a^, ^b^, ^c^	Increasing distance to natural habitat may reduce pollinator species diversity in TSF, but there is low certainty in this conclusion.
Fruit set (proportion of flowers developed into fruits)	Based on 405 data points from 17 studies	Slope: −0.07 (−0.17 to 0.04) *p* = 0.20 Distance 0–8676 m (maximum distance median = 500 m, mean = 1557 m)	⊕⊝⊝⊝ very low^a^, ^b^, ^c^, ^d^	Increasing distance to natural habitat may not influence fruit set of pollinator‐dependent crops in TSF, but there is low certainty in this conclusion.

^a^ Concerns with high risk of bias in studies.

^b^ High heterogeneity among studies.

^c^ Indirectness concerns due to variability in pollinator taxa (for pollinator abundance and richness) and spatial scales of distance to natural habitat.

^d^ Imprecision concerns due to wide confidence intervals.

## 4 Discussion

Smallholder farms make up 84% of all farms worldwide (Lowder et al. 2021) and are a key priority for reducing poverty and hunger while sustainably managing natural resources in the tropics (United Nations 2015). These farms rely heavily on biodiversity‐derived ecosystem services such as pollination for nutrition and food security (Tibesigwa et al. 2019; Timberlake et al. 2022; Mulungu et al. 2023). Understanding how to support pollination services provided by wild insects in TSF landscapes is therefore important, both for informing landscape conservation management strategies and for supporting farmers to sustain their livelihoods. Our systematic review and meta‐analysis, encompassing 35 studies, investigated the relationship between distance to natural habitat and pollination services in TSFs. We found no consistent decline in pollinator abundance (31 studies) or fruit set (17 studies) with increasing distance to natural habitat. Our results revealed weak evidence of a negative association between natural habitat proximity and pollinator species richness (30 studies), where the high heterogeneity in effect sizes across studies indicates these are highly context‐dependent and locally variable.

These findings stand in contrast to previous global syntheses that primarily focused on large‐scale farms and have shown consistent declines in pollinators and pollination services, particularly in the tropics (Ricketts et al. 2008; Garibaldi et al. 2011). Two possible explanations for these contrasting results emerge from our synthesis: firstly, TSF landscapes may provide resilience to and buffer against the negative effects of increasing distance to natural habitat on pollination services; and secondly, the complexities and methodological variation across studies may limit our ability to detect consistent patterns.

### 4.1 Smallholder Landscapes May Buffer Pollination Services Against Increasing Distance to Natural Habitat

The lack of a consistent decline in pollination services with distance to natural habitat in TSF landscapes may reflect the stark differences between tropical smallholder farming systems and more industrial agricultural landscapes. While previous syntheses reported clear declines in pollination services with increasing distance to natural habitat (Ricketts et al. 2008; Garibaldi et al. 2011), many of their underlying studies provided data from larger‐scale, commercially managed farms. In contrast, TSFs are characterised by small field sizes, relatively high crop diversity and flower‐rich herbaceous semi‐natural habitat patches, creating a high degree of landscape heterogeneity (Perfecto and Vandermeer 2010; Tscharntke et al. 2012). Our findings therefore align with the ecological contrast hypothesis, which predicts weaker local responses in heterogeneous farming landscapes (Kleijn et al. 2011; Marja et al. 2019). They also underscore that TSFs themselves could provide habitat for insect pollinators. Specifically, TSFs provide a pollinator‐friendly mosaic of vegetation and habitat types with diverse nesting sites, staggered floral resources across time and sheltering areas (e.g., Tamburini et al. 2020; von Königslöw et al. 2021; Astegiano et al. 2024; Marrero et al. 2024; Fijen et al. 2025). Furthermore, agroforestry practices that integrate multipurpose native trees and shrubs alongside crops are widespread in TSF landscapes (Nair et al. 2021), offering a variety of additional floral resources and specialised nesting sites that can support wild pollinators and enhance pollination services (Anders et al. 2023; Kingazi et al. 2024). At the same time, natural habitats are often not free from human influence (e.g., grazing, firewood collection), which further blurs the line between natural and managed areas. Consequently, the contrast between natural and cultivated habitats in tropical landscapes is less distinct than in temperate or more intensive agricultural systems. These landscapes may thus buffer effects of habitat loss, making distance to natural habitat a less significant factor than in more simplified landscapes.

We found no decline in the total number of pollinators with increasing distance to natural habitat, but a decline in pollinator species richness, suggesting that human‐modified TSF landscapes may favour fewer highly abundant species, whereas rarer, potentially more specialised wild pollinators decline with increasing distance to natural habitat. Functional redundancy among pollinators may help explain the lack of an effect on crop fruit set, assuming that the fruit set of many tropical crops is pollination limited. In some cases, dominant pollinator species can partially compensate for those pollinators that decline, maintaining pollination services to a certain extent (Yachi and Loreau 1999; Memmott et al. 2004). For example, crops in tropical Asia, South America and Africa increasingly rely on managed and feral Africanised honeybee colonies for pollination (Calfee et al. 2020; Phiri et al. 2022). Using honeybees for crop pollination comes with a suite of drawbacks, however, including high colony mortality, negative impacts on native, non‐managed pollinators (Aizen et al. 2020; Osterman et al. 2021), reduced pollination effectiveness (Klein et al. 2003a; Garibaldi et al. 2013) and lower resilience against environmental fluctuations compared to species‐rich wild pollinator communities (Dainese et al. 2019; Woodcock et al. 2019).

### 4.2 Methodological Limitations May Obscure Pollination Patterns With Increasing Distance to Natural Habitat in Tropical Smallholder Farms

Methodological limitations and variability of the included studies could be an alternative or complementary explanation for the weak overall effect observed, reflected by our ‘very low’ certainty of evidence assessments for all three outcomes (Table 2). Although the CEECAT risk of bias tool proved difficult to apply consistently—lacking sensitivity to contextual nuance and practical constraints of ecological field research—it nonetheless provided a structured format to qualitatively identify important methodological limitations in the evidence base. These included difficulties in controlling for major confounding factors, standardising spatial scales, and ensuring sufficiently large sample sizes, all of which can contribute to variability in effect sizes. These challenges are likely compounded by the fact that smallholder farming landscapes across the tropics are, by definition, highly diverse environments (FAO 2017), as is the case in our review (Table 1). As a result, detecting landscape‐scale patterns in pollination dynamics may be methodologically more complex in TSF landscapes compared to larger, more homogenised farming systems (Steward et al. 2014).

Theoretically, our subgroup analyses should have helped us identify drivers of heterogeneity, but lack of detailed data limited our ability to examine more nuanced patterns. For example, pollinator guilds differ in reliance on proximity to natural habitat due to functional traits such as body size, foraging range and sociality (Gathmann and Tscharntke 2002; Steffan‐Dewenter et al. 2002; Steffan‐Dewenter and Kuhn 2003), meaning that spatial scales at which distance to natural habitat is measured should align with the biology of the focal pollinator groups (e.g., Basu et al. 2016; Hass et al. 2018). However, limited availability of and access to detailed raw data prevented subgroup analyses beyond the exclusion of managed honeybees. Studies also varied widely in their definitions of natural habitat, with over half of the studies measuring distance to the nearest natural forest or forest fragments, and others focusing on shrublands, wetlands, agroforests, and other semi‐natural habitats. Although our moderator analysis detected no significant effects between ‘natural forest’ and ‘other’ habitat types, the variability within the ‘other’ category may have obscured effects of specific habitats. Moreover, additional characteristics such as habitat size, age, vegetation composition (e.g., native vs. non‐native) or forest canopy density are likely to influence pollinator responses (e.g., Moreaux et al. 2021). Additional confounding variables, including pesticide use (Basu et al. 2016; Obregon et al. 2021), seasonality (Banks et al. 2013; Banks et al. 2014) or local floral abundance (Schrader et al. 2018; Wayo et al. 2020), were reported only by a small subset of studies, with varying data resolution across studies, constraining cross‐study comparisons.

### 4.3 Future Research Directions

Our systematic review and meta‐analysis found uncertain evidence of strong relationships between proximity to natural habitat and pollination services in TSFs (Table 2). Given this uncertainty, we suggest two complementary directions for future research into the factors that maintain biodiversity and ecosystem services such as pollination in tropical smallholder farms. First, we encourage researchers to broaden their focus from (but not abandon) the proximity to natural habitat hypothesis. Distance to natural habitat alone may be too coarse to capture pollination dynamics in complex smallholder landscapes, emphasizing the importance of more nuanced metrics such as landscape heterogeneity and connectivity. To move beyond broad‐scale patterns, future research should prioritise context‐sensitive, community‐level studies in under‐represented areas. Secondly, we encourage concerted methodological and reporting improvements in this line of research. Our synthesis highlights the need for more methodological unification and standardization, alongside greater transparency in reporting methods (e.g., pollinator traits, natural habitat characteristics, farm management practices) and results (including open sharing of data and code). Advances in remote sensing and embedded monitoring now offer promising tools to support this (e.g., Darras et al. 2024). Making such improvements will increase the utility of primary research in this area, as well as evidence syntheses like our own (which may be extended to a direct comparison between tropical smallholder and larger‐scale farming). Collectively, we believe these actions will strengthen evidence‐based conservation and be valuable for informing landscape management strategies and priorities that balance agricultural productivity with biodiversity conservation.

## Author Contributions

**Ennia Bosshard:** conceptualisation, Data curation, Formal analysis, Funding acquisition, Investigation, Methodology, Project administration, Validation, Visualisation, Writing – Original draft, Writing – Review and Editing. **Mark E. Harrison:** conceptualisation, Investigation, Validation, Writing – Review and Editing, Supervision. **Frank van Veen:** conceptualisation, Writing – Review and Editing, Supervision. **Chris J. Kettle:** funding acquisition, Writing – Review and Editing, Supervision. **Nagaraja Badenahally Chikkarangappa:** resources. **John E. Banks:** resources. **Parthiba Basu:** resources. **Quebin Bosbely Casiá‐Ajché:** resources. **Bo Dalsgaard:** resources. **Aditi Dutta:** resources. **Eunice Enríquez:** resources. **Natalia Escobedo‐Kenefic:** resources, Writing – Review and Editing. **Hugo Eduardo Fierros‐López:** resources. **Barbara Gemmill‐Herren:** resources. **Jaboury Ghazoul:** resources, Writing – Review and Editing. **Katrine Hansen:** resources. **Annika L. Hass:** resources, Writing – Review and Editing. **Juliana Hipólito:** writing – Review and Editing, Resources. **Oliver Honnay:** resources, Writing – Review and Editing. **John Muo Kasina:** resources. **Alexandra‐Maria Klein:** resources, Writing – Review and Editing. **Iris Kormann Motzke:** resources. **Smitha Krishnan:** resources, Writing – Review and Editing. **Patricia Landaverde‐Gonzalez:** resources, Writing – Review and Editing. **Anderson Oliveira Latini:** resources, Writing – Review and Editing. **Kevin Li:** resources, Writing – Review and Editing. **Rodrigo Lucas‐García:** resources, Writing – Review and Editing. **Theodore Munyuli:** resources. **Deepthi Narasimhaiah:** resources. **Diana Obregon:** resources, Writing – Review and Editing. **J. Javier G. Quezada‐Euán:** resources, Writing – Review and Editing. **Mónica E. Riojas‐López:** resources. **Victor Rosas‐Guerrero:** resources, Writing – Review and Editing. **Julian Schrader:** resources, Writing – Review and Editing. **Fernando Severiano‐Galeana:** resources, Writing – Review and Editing. **Tegegne Molla Sitotaw:** resources. **Tuanjit Sritongchuay:** resources. **Pornpimon Tangtorwangsakul:** resources. **Manuel Toledo‐Hernandez:** resources. **Teja Tscharntke:** writing – Review and Editing, Resources. **Poornima Viswanathan:** resources. **Cassandra Vogel:** resources, Writing – Review and Editing. **Thomas C. Wanger:** writing – Review and Editing, Resources. **Kanuengnit Wayo:** resources. **Catrin Westphal:** resources, Writing – Review and Editing. **Matt Lloyd Jones:** conceptualisation, Investigation, Methodology, Validation, Writing – Review and Editing, Supervision. **Christopher N. Kaiser‐Bunbury:** conceptualisation, Methodology, Funding acquisition, Writing – Review and Editing, Supervision.

## Conflicts of Interest

The authors declare no conflicts of interest.

## Supporting information


**Data S1:** ele70229‐sup‐0001‐DataS1.docx.

## Data Availability

Raw data and R code for conducting the meta‐analysis are available via GitHub (https://github.com/enniabosshard/pollinatorhabitatTSF_SRMA/), and the version used for this manuscript is permanently archived at Zenodo (https://doi.org/10.5281/zenodo.15088749).
