# Supplementary material for: Proximity to Natural Habitat Is Not Consistently Associated With Pollination Services in Tropical Smallholder Farms: A Systematic Review and Meta‐Analysis
Source: Ecol Lett. 2025 Dec 3;28(12):e70229. doi: 10.1111/ele.70229 (PMC12675873; doi:10.1111/ele.70229)
Supplement: Supplementary file 1 — Data S1: ele70229‐sup‐0001‐DataS1.docx. [file ELE-28-0-s001.docx]

SUPPORTING INFORMATION

**Proximity to natural habitat is not consistently associated with pollination services in tropical smallholder farms: A systematic review and meta-analysis**

Ennia Bosshard, Mark E. Harrison, Frank van Veen, Chris J Kettle, Nagaraja Badenahally Chikkarangappa, John E Banks, Quebin Bosbely Casiá-Ajché, Bo Dalsgaard, Narasimhaiah Deepthi, Aditi Dutta, Eunice Enríquez, Natalia Escobedo-Kenefic, Hugo Eduardo Fierros-López, Barbara Gemmill-Herren, Jaboury Ghazoul, Katrine Hansen, Annika L Hass, Juliana Hipólito, Oliver Honnay, John Muo Kasina, Alexandra-Maria Klein, Iris Kormann Motzke, Smitha Krishnan, Patricia Landaverde, Anderson Oliveira Latini, Kevin Li, Rodrigo Lucas-Garcia, Theodore Munyuli, Diana Obregon, J. Javier G. Quezada-Euán, Mónica E. Riojas-López, Victor Rosas-Guerrero, Julian Schrader, Fernando Severiano-Galeana, Tegegne Molla Sitotaw, Tuanjit Sritongchuay, Pornpimon Tangtorwangsakul, Manuel Toledo-Hernandez, Teja Tscharntke, Poornima Viswanathan, Cassandra Vogel, Thomoas C. Wanger, Kanuengnit Wayo, Catrin Westphal, Matt Lloyd Jones*, Christopher N. Kaiser-Bunbury*

*shared last/senior authorshipf

Table of Contents

[1. Systematic literature review 5](#_Toc207627948)

[1.1. Inclusion and exclusion criteria 5](#_Toc207627949)

[1.2. Search strings and databases searched 7](#_Toc207627950)

[2. Preregistration deviation 8](#_Toc207627951)

[3. Prisma 2020 checklist 10](#_Toc207627952)

[4. Standardisation of isolation measurements to distance 14](#_Toc207627953)

[5. List of studies excluded with reason 18](#_Toc207627954)

[6. Study design types 26](#_Toc207627955)

[7. Risk of bias assessment 27](#_Toc207627956)

[8. Meta-analysis 30](#_Toc207627957)

[8.1. Pollinator abundance 30](#_Toc207627958)

[8.2. Pollinator richness 39](#_Toc207627959)

[8.3. Fruit set 46](#_Toc207627960)

[9. Sensitivity analyses 51](#_Toc207627961)

[9.1. Pollinator abundance 53](#_Toc207627962)

[9.2. Pollinator richness 56](#_Toc207627963)

[9.3. Fruit set 59](#_Toc207627964)

[10. Certainty assessment 62](#_Toc207627965)

[11. Funding statements for data-contributing authors 64](#_Toc207627966)

[12. References 66](#_Toc207627967)

**List of tables**

[**Table S1.** Overview of inclusion and exclusion criteria following the PECO-type systematic review criteria (Morgan et al. 2018). 5](#_Toc207627969)

[**Table S2.** Bibliographic searches conducted on 22^nd^ December 2024. 7](#_Toc207627970)

[**Table S3.** Pre-registration deviations table based on the template by Willroth and Atherton (2024). 8](#_Toc207627971)

[**Table S4.** Prisma checklist (Page et al. 2021) 10](#_Toc207627972)

[**Table S5.** Overview of studies for which distances were successfully derived using satellite imagery. 15](#_Toc207627973)

[**Table S6.** Overview of studies excluded with reason at the full-text stage (n = 122) during the systematic review 18](#_Toc207627974)

[**Table S7.** Overview of studies excluded because we were unable to obtain data for meta-analysis (n = 26) 24](#_Toc207627975)

[**Table S8.** Effect sizes and moderator variables for the meta-analysis on pollinator abundance (N = 31 studies) 33](#_Toc207627976)

[**Table S9.** Overview of the model outputs for the main abundance model, wild abundance model, two moderator models (habitat type and agricultural intensity), and the three sensitivity models (pollinator sampling method, distance measure, and maximum distance scales). Sample size (N) for each model or category is provided. Model outputs include the estimated effect sizes, standard errors (SE), z-values, p-values and 95% confidence intervals. Significance codes: * p < 0.05; ** p < 0.01. 35](#_Toc207627977)

[**Table S10.** Results of the heterogeneity analysis for the main model, wild abundance model, moderator model (agricultural intensity), and the three sensitivity models (pollinator sampling method, distance measure, and maximum distance scales). Heterogeneity measures: tau^2^ = estimated amount of residual heterogeneity; tau = square root of estimated tau^2^ value; I^2^ = residual heterogeneity / unaccounted variability; H^2^ = unaccounted variability / sampling variability; Q test = Test for Heterogeneity; For moderator analyses: QE = Test for residual Heterogeneity, QM = Test of Moderators 36](#_Toc207627978)

[**Table S11.** Effect sizes and moderator variables used for the meta-analysis on abundance of wild pollinators, excluding managed honeybees (N= 28 studies). 37](#_Toc207627979)

[**Table S12.** Overview of effect sizes and moderator variables for the pollinator richness meta-analysis (N= 30 studies) 42](#_Toc207627980)

[**Table S13.** Overview of the model outputs for the pollinator richness model, two moderator models (habitat type and agricultural intensity), and the four sensitivity models (pollinator sampling method, distance measure, maximum distance scales, and taxonomic resolution of species identification). Sample size (N) for each model or category is provided. Model outputs include the estimated effect sizes, standard errors (SE), z-values, p-values and 95% confidence intervals. Significance codes: * p < 0.05; ** p < 0.01. 44](#_Toc207627981)

[**Table S14.** Results of the heterogeneity analysis for the main model, wild richness model, two moderator models (habitat type and agricultural intensity), and the four sensitivity models (pollinator sampling method, distance measure, maximum distance scales, and taxonomic resolution of species identification). Heterogeneity measures: tau^2^ = estimated amount of residual heterogeneity; tau = square root of estimated tau^2^ value; I^2^ = residual heterogeneity / unaccounted variability; H^2^ = unaccounted variability / sampling variability; Q test = Test for Heterogeneity; For moderator analyses: QE = Test for residual Heterogeneity, QM = Test of Moderators 45](#_Toc207627982)

[**Table S15.** Overview of effect sizes and moderator variables for the fruit set meta-analysis (N = 17 studies). 48](#_Toc207627983)

[**Table S16.** Overview of the model outputs for the fruit set model, three moderator models (habitat type, agricultural intensity, and crop pollinator-dependency), and the two sensitivity models (distance measure, and maximum distance scales,). Sample size (N) for each model or category is provided. Model outputs include the estimated effect sizes, standard errors (SE), z-values, p-values and 95% confidence intervals. 49](#_Toc207627984)

[**Table S17.** Results of the heterogeneity analysis for the fruit set model, three moderator models (habitat type, agricultural intensity, and crop pollinator-dependency), and the two sensitivity models (distance measure, maximum distance scales). Heterogeneity measures: tau^2^ = estimated amount of residual heterogeneity; tau = square root of estimated tau^2^ value; I^2^ = residual heterogeneity / unaccounted variability; H^2^ = unaccounted variability / sampling variability; Q test = Test for Heterogeneity; For moderator analyses: QE = Test for residual Heterogeneity, QM = Test of Moderators 50](#_Toc207627985)

[**Table S18.** Leave-one-out sensitivity analysis for the meta-analysis on pollinator abundance (N = 31 studies). The table reports the effect size estimate (estimate), standard error (se), z-value (zval), and 95% confidence interval (ci.lb, ci.ub) after excluding each study. Heterogeneity measures include Cochran’s Q statistic (Q) and p-value (Qp), between-study variance (tau²), proportion of variation due to heterogeneity (I²), and total-to-sampling variability ratio (H²). 54](#_Toc207627986)

[**Table S19.** Leave-one-out sensitivity analysis for the meta-analysis on pollinator richness (N = 30 studies). The table reports the effect size estimate (estimate), standard error (se), z-value (zval), and 95% confidence interval (ci.lb, ci.ub) after excluding each study. Heterogeneity measures include Cochran’s Q statistic (Q) and p-value (Qp), between-study variance (tau²), proportion of variation due to heterogeneity (I²), and total-to-sampling variability ratio (H²). 57](#_Toc207627987)

[**Table S20.** Leave-one-out sensitivity analysis for the meta-analysis on fruit set (N= 17 studies). The table reports the effect size estimate (estimate), standard error (se), z-value (zval), and 95% confidence interval (ci.lb, ci.ub) after excluding each study. Heterogeneity measures include Cochran’s Q statistic (Q) and p-value (Qp), between-study variance (tau²), proportion of variation due to heterogeneity (I²), and total-to-sampling variability ratio (H²). 60](#_Toc207627988)

[**Table S21.** Overview of justification for GRADE assessment following the Cochrane guidelines (Schünemann et al. 2023). 62](#_Toc207627989)

[**Table S22.** Summary of the funding sources acknowledged by each data-contributing author for the original research from which they shared data for this systematic review and meta-analysis. 64](#_Toc207627990)

**List of figures**

[**Figure S1.** Decision tree for the ‘smallholder farms’ study inclusion criteria 6](#_Toc207627993)

[**Figure S2.** Examples of distances derived from satellite imagery for the dataset by Landaverde-Gonzalez et al. (2017) who studied farmers’ chilli fields across a gradient of forest loss. We derived distances to the nearest forest edge using historic satellite imagery as close as possible to the study’s data collection period (2010-2011). 17](#_Toc207627994)

[**Figure S3.** Data and model fits for the relationship between pollinator abundance and linear distance to the nearest natural habitat of each study using either GLMs or GLMMs with negative binomial error distribution (N = 31 studies; modelling approach indicated in the title of each plot). 30](#_Toc207627995)

[**Figure S4.** Data and model fits for the relationship between pollinator richness and linear distance to the nearest natural habitat of each study using either GLMs or GLMMs with negative binomial error distribution (N= 30 studies; modelling approach indicated in the title of each plot). 39](#_Toc207627996)

[**Figure S5.** Data and model fits for the relationship between fruit set and distance to the nearest natural habitat of each study using beta regression models (GLMs for single-distance-per-site datasets and GLMMs for datasets with nested and paired study designs; N = 17 studies). 46](#_Toc207627997)

[**Figure S6.** **Left:** Influential study analysis for the pollinator abundance meta-analysis (N = 31 studies) using the influence() function from the *metafor* package in R. Each data point represents one study included in the meta-analysis, and red dots represent studies flagged as influential based on key influence diagnostics, such as studentised residuals, Cook’s distance, and covariance ratios metrics (Viechtbauer and Cheung 2010). **Right:** Normal Q-Q plot of residuals from the random-effects meta-analysis model. The plot compares the standardised residuals to a theoretical normal distribution. It indicates that the residuals conform to normality, suggesting that the model assumptions are appropriately met. 53](#_Toc207627998)

[**Figure S7.** Funnel plot for the pollinator abundance meta-analysis. A symmetric distribution of effect sizes suggests no strong evidence of publication bias. 55](#_Toc207627999)

[**Figure S8.** Left: Influential study analysis for the meta-analysis on pollinator species richness (N= 30 studies) using the influence() function from the *metafor* package in R. Each data point represents one study included in the meta-analysis, and red dots represent studies flagged as influential based on key influence diagnostics, such as studentised residuals, Cook’s distance, and covariance ratios metrics (Viechtbauer and Cheung 2010). **Right:** Normal Q-Q plot of residuals from the random-effects meta-analysis model. The plot compares the standardised residuals to a theoretical normal distribution. It indicates that the residuals conform to normality, suggesting that the model assumptions are appropriately met. 56](#_Toc207628000)

[**Figure S9.** Funnel plot for the pollinator species richness meta-analysis. 58](#_Toc207628001)

[**Figure S10.** Left: Influential study analysis for the meta-analysis on fruit set (N= 17 studies) using the influence() function from the *metafor* package in R. Each data point represents one study included in the meta-analysis, and red dots represent studies flagged as influential based on key influence diagnostics, such as studentised residuals, Cook’s distance, and covariance ratios metrics (Viechtbauer and Cheung 2010). **Right:** Normal Q-Q plot of residuals from the random-effects meta-analysis model. The plot compares the standardised residuals to a theoretical normal distribution. The plot indicates that the residuals conform to normality, suggesting that the model assumptions are appropriately met. 59](#_Toc207628002)

[**Figure S11.** Funnel plot for the fruit set meta-analysis. 61](#_Toc207628003)

# Systematic literature review

## Inclusion and exclusion criteria

**Table S1.** Overview of inclusion and exclusion criteria following the PECO-type systematic review criteria (Morgan et al. 2018).

| **PECO criteria** | **Include** | **Exclude** |
| --- | --- | --- |
| Population | Studies focused on insect pollinator and/or insect-pollinated crops in TSFs. | Studies focusing on other pollinators apart from insects, e.g. bats or birds. |
|  | Studies explicitly describing farms as ‘smallholder’, ‘small-scale’ or ‘subsistence’ farms, terms often used interchangeably (FAO 2017), and studies reporting farm sizes of ≤ 2ha, a frequently adopted size threshold (FAO 2017). We also considered farms ≤ 15ha as TSFs if they met at least two of the following characteristics: a) low external inputs, b) high crop diversity, and/or c) produce use mainly intended for household and local markets. These criteria were informed by existing literature (FAO 2017, Lowder et al. 2021) and adapted to reflect data availability. When classification was uncertain, we also consulted corresponding authors to confirm whether their study sites met our smallholder farm criteria. | Studies that did not observe pollination services within a smallholder farm (e.g. within ‘large-scale’, ‘commercial’ or ‘monoculture’ farms, or farms >15ha) or within a forest, park, or other non-farm areas |
|  | Studies located in tropical regions, i.e. if they fell between the Tropics of Cancer (23°27) and Capricorn (23°27). | Studies conducted outside of tropical regions. |
| Exposure/  Comparator | Studies compared pollination variables in TSFs with varying degrees of isolation from natural habitat . We defined ‘natural habitat’ based on the classification used in the original studies, without imposing a standardised definition. What constituted natural habitat thus varied depending on how it was defined by the respective study authors (e.g., forests, grasslands, or other semi-natural areas). Isolation could be measured either via distance to nearest natural habitat; categories such as ‘near’ or ‘far’ (or similar), or as the proportion of natural habitat within a specific radius around the farm. | Studies examining effects of field margins, shade, tree cover, or other landscape features outside of natural habitat distance/proportion. |
| Outcome | Studies assessed at least one of three proxies associated with insect pollination in these farms; pollinator abundance (count of number of individual pollinators recorded within a defined time frame and area), pollinator species richness (count of pollinator species), or fruit/seed set of pollinator crops (proportion of flowers that successfully developed into fruits or seeds). | Studies that did not measure any of these response variables. |
| Study design | Empirical field studies | Predictive modelling studies, reviews and meta-analyses |
| Other | Studies published in English | Studies published in other languages |
|  | Studies report sufficient data to permit our meta-analysis, or make available upon request | Studies for which we were unable to retrieve sufficient raw data |

**Figure S1.** Decision tree for the ‘smallholder farms’ study inclusion criteria

- 1. Search strings and databases searched

**Table S2.** Bibliographic searches conducted on 22^nd^ December 2024.

| **Databases** | **Search scope** | **Search strings** | **N. of**  **hits** |
| --- | --- | --- | --- |
| Web of Science Core Collection:  Science Citation Index Expanded (SCI-EXPANDED) (1900–present);  Social Sciences Citation Index (SSCI) (1900–present);  Arts & Humanities Citation Index (AHCI) (1975–present); Conference Proceedings Citation Index – Science (CPCI-S) (1990–present); Conference Proceedings Citation Index – Social Science & Humanities (CPCI-SSH) (1990–present); Emerging Sources Citation Index (ESCI) (2015–present) | Topic (Searches title, abstract, keyword plus, and author keywords) | pollinat* AND (abundan* OR richness OR visit* OR divers* OR "fruit* NEAR set*" OR "seed* NEAR set*" ) AND (agricult* OR smallhold* OR farm* OR crop* OR garden* OR orchard* OR subsistence* OR homestead* ) AND (distan* OR isolat* OR proximit* OR adjacen* OR connect* OR far* OR close* OR near* OR landscape OR spatial* OR proport* ) AND (habitat* OR "natural vegetat*" OR forest* OR shrub* OR wood* OR grass* OR hedg*) AND (tropic* OR "Central America*" OR "South America*" OR Beliz* OR Bolivia* OR Brazil* OR Colombia* OR "Costa Rica*" OR Cuba* OR "Dominican Republic*" OR Ecuador* OR "El Salvador*" OR "French Guiana*" OR Guatemala* OR Guyana* OR Haiti* OR Hondura* OR Jamaica* OR Nicaragua* OR Panama* OR Paraguay* OR Peru* OR Surinam* OR "Trinidad and Tobago*" OR Venezuela* OR "Sub-Saharan Africa*" OR Angola* OR Benin* OR Botswana* OR "Burkina Faso*" OR Burundi* OR Cameroon* OR "Cape Verd*" OR "Central African Republic*" OR Chad* OR Comoro* OR Congo* OR "Democratic Republic of the Congo*" OR Djibouti* OR "Equatorial Guinea*" OR Eritrea* OR Eswatini* OR Ethiopia* OR Gabon* OR Gambia* OR Ghana* OR Guinea* OR Guinea-Bissau* OR "Ivory Coast*" OR Kenya* OR Lesoth* OR Liberia* OR Madagascar* OR Malawi* OR Mali* OR Mauritania* OR Mauriti* OR Mozambi* OR Namibia* OR Niger* OR Nigeria* OR Rwanda* OR "Sao Tome and Principe*" OR Senegal* OR Seychell* OR "Sierra Leon*" OR Somalia* OR "South Africa*" OR "South Sudan*" OR Tanzania* OR Togo* OR Uganda* OR Zambia* OR Zimbabwe* OR "South east Asia*" OR Bangladesh* OR Brunei* OR Cambodia* OR "East Timor*" OR India* OR Indonesia* OR Lao* OR Malaysia* OR Maldiv* OR Myanmar* OR Nepal* OR "Papua New Guinea*" OR Philippine* OR Singapore* OR "Sri Lanka*" OR Thailand* OR Vanuatu* OR Vietnam*) | 525 |
| Scopus | article title, abstract and keywords. |  | 464 |
| Ovid CAB abstracts | 1973 to 2024 week 51  mp. [mp=abstract, title, original title, broad terms, heading words, cabicodes words] |  | 784 |

# Preregistration deviation

**Table S3.** Pre-registration deviations table based on the template by Willroth and Atherton (2024).

| **#** | **Details** | | **Original Wording** | **Deviation Description** | **Reader Impact** |
| --- | --- | --- | --- | --- | --- |
| 1 | Type | Eligibility criteria | “Eligibility criteria for study inclusion were that (1) the study measured at least one metric associated with crop pollination services, namely pollinator richness, abundance, visitation rates, fruit or seed set in relation to isolation from natural vegetation; (2) the crops were pollinated by insects; (3) the study took place in a tropical country; and (4) the study was located on a small-scale farm, with the criteria “small-scale” being met if the farm was described in the original publication as “subsistence”, “smallholder” or “small-scale” farm or measured less than 15 hectares in total size.” | We refined the eligibility criteria to ensure consistency and clarity, incorporating additional specifications that had been implicitly applied but were not explicitly stated in the pre-registration. The updated eligibility criteria are outlined in the methods section 2.2. | This deviation arose from an oversight in not explicitly detailing the eligibility criteria when writing the preregistration. It does not affect readers’ interpretation of the findings, as the eligibility criteria remained consistent and there were no changes to the criteria for inclusion/exclusion of studies . |
|  | Reason | Typo/Error |  |  |  |
|  | Timing | During data collection |  |  |  |
| 2 | Type | Data collection procedure | “Between February and April 2022, a systematic search of the literature was performed using six different databases with the aim to be comprehensive. To search for the landscape effect on crop pollination services in tropical agroecosystems, the following Boolean search terms were used: (Pollinat* OR pollination service) AND (tropic*) AND (distance OR isolation OR proximity OR proportion OR (semi-) natural habitat OR forest) AND (yield OR fruit set OR crop OR produc*) AND (agricult* OR smallhold* OR farm* OR home garden OR subsistence). Additionally, all studies that were cited in previously published literature reviews on this topic were reviewed (Ricketts et al. 2008, Garibaldi et al. 2011, Kleijn et al. 2015, Moreaux et al. 2022). Collectively, these searches resulted in 97 potentially relevant studies that were further screened for eligibility.” | We initially deployed (and screened the results of) a simpler search strategy between February and April 2022, which we pre-registered in November 2022 (Bosshard et al. 2022), and intended to re-run as a straightforward search update near to submission in order to capture new publications. However, when MLJ joined the team in May 2024, he raised concerns about the comprehensiveness of the original search strategy and its reporting. Therefore, as part of our wider efforts to align the systematic review with current standards in evidence synthesis, instead of updating the original searches, we took the opportunity to design and run new searches in December 2024. Recognising the value they add to the systematic review process (Rethlefsen et al. 2015), a librarian/information specialist was consulted on the redesign process (though they did not have capacity to join the team as a co-author; see Acknowledgements). | The deviation is justifiable because the original searches were not conducted or reported to current systematic review standards, and were pre-registered after being performed and screened. The improved and updated literature search has made the study more systematic and allowed to include additional studies that were previously overlooked. This means that the analysis likely captures a more comprehensive and diverse dataset, potentially improving the generalisability and robustness of the results. |
|  | Reason | Typo/Error |  |  |  |
|  | Timing | During data collection |  |  |  |
| 3 | Type | Sample | “The screening for eligibility identified 25 studies that met my inclusion criteria. All articles lacked some information necessary for the statistical analysis. Original datasets were therefore requested from the corresponding authors of the 25 studies. Given a relatively low reply rate by the lead authors of the primary publications, several studies had to be excluded from this study.” | The improved searches described above under point #2 resulted in a larger number of potentially relevant studies than initially identified in the pre-registration. We updated the systematic review process to adhere more with best practice and employed a more rigorous and partial double-blinded screening process to identify suitable studies for the meta-analysis. | A more rigorous screening process should improve trust in the findings of the systematic review and meta-analysis results, since the included studies are identified according to a more systematic process. |
|  | Reason | New knowledge |  |  |  |
|  | Timing | After data access |  |  |  |
| 4 | Type | Analysis | “In the second stage, the aggregated data will be combined to produce the summary results. For the statistical analysis, Bayesian multilevel models will be used to account for variation in the heterogeneity parameters. Bayesian methods are ideally suited for meta-analyses which include a small number of studies due to improved tractability relative to frequentist alternatives (Meager 2019) and to avoid the common problems associated with null hypothesis testing (Gelman 2018).” | While we maintained a two-stage individual participant data (IPD)-style approach for the meta-analysis, we were unable to implement the Bayesian methods as initially planned. This was due to the team’s limited experience with Bayesian techniques. Instead, we adopted a more frequently used and well-documented frequentist approach using the ‘metafor’ package in R for the second stage of the analysis. This approach is methodologically similar to the originally-planned Bayesian approach. | This deviation may have minor implications for the findings of the meta-analysis. Bayesian methods, as originally planned, offer advantages such as improved handling of small sample sizes and more nuanced estimates of uncertainty. However, by instead using a frequentist approach with the well-documented ‘metafor’ package in R, we ensured the analysis was robust and accessible, given the team’s expertise. Additionally, the frequentist approach is more commonly used, well-documented, and easier for readers to understand and reproduce. Both methods are widely accepted in meta-analysis, but may differ in their assumptions and interpretive framework. |
|  | Reason | Plan not possible |  |  |  |
|  | Timing | After data access |  |  |  |

# Prisma 2020 checklist

**Table S4.** Prisma checklist (Page et al. 2021)

| **Section and Topic** | **Item #** | **Checklist item** | **Location where item is reported** |
| --- | --- | --- | --- |
| **TITLE** | | |  |
| Title | 1 | Identify the report as a systematic review. | Page 1 |
| **ABSTRACT** | | |  |
| Abstract | 2 | See the PRISMA 2020 for Abstracts checklist. | Page 5 |
| **INTRODUCTION** | | |  |
| Rationale | 3 | Describe the rationale for the review in the context of existing knowledge. | Pages 6–8 |
| Objectives | 4 | Provide an explicit statement of the objective(s) or question(s) the review addresses. | Pages 7–8 |
| **METHODS** | | |  |
| Eligibility criteria | 5 | Specify the inclusion and exclusion criteria for the review and how studies were grouped for the syntheses. | Pages 9–10;  Table S1 |
| Information sources | 6 | Specify all databases, registers, websites, organisations, reference lists and other sources searched or consulted to identify studies. Specify the date when each source was last searched or consulted. | Pages 10-11;Table S2 |
| Search strategy | 7 | Present the full search strategies for all databases, registers and websites, including any filters and limits used. | Pages 10-11;Table S2 |
| Selection process | 8 | Specify the methods used to decide whether a study met the inclusion criteria of the review, including how many reviewers screened each record and each report retrieved, whether they worked independently, and if applicable, details of automation tools used in the process. | Page 11 |
| Data collection process | 9 | Specify the methods used to collect data from reports, including how many reviewers collected data from each report, whether they worked independently, any processes for obtaining or confirming data from study investigators, and if applicable, details of automation tools used in the process. | Page 12 |
| Data items | 10a | List and define all outcomes for which data were sought. Specify whether all results that were compatible with each outcome domain in each study were sought (e.g. for all measures, time points, analyses), and if not, the methods used to decide which results to collect. | Pages 12–15 |
|  | 10b | List and define all other variables for which data were sought (e.g. participant and intervention characteristics, funding sources). Describe any assumptions made about any missing or unclear information. | Pages 12–15 |
| Study risk of bias assessment | 11 | Specify the methods used to assess risk of bias in the included studies, including details of the tool(s) used, how many reviewers assessed each study and whether they worked independently, and if applicable, details of automation tools used in the process. | Pages 15-16, Appendix 7 |
| Effect measures | 12 | Specify for each outcome the effect measure(s) (e.g. risk ratio, mean difference) used in the synthesis or presentation of results. | Pages 16–17 |
| Synthesis methods | 13a | Describe the processes used to decide which studies were eligible for each synthesis (e.g. tabulating the study intervention characteristics and comparing against the planned groups for each synthesis (item #5)). | Pages 16–17 |
|  | 13b | Describe any methods required to prepare the data for presentation or synthesis, such as handling of missing summary statistics, or data conversions. | Pages12–15;  Appendix 4 |
|  | 13c | Describe any methods used to tabulate or visually display results of individual studies and syntheses. | Page 17 |
|  | 13d | Describe any methods used to synthesize results and provide a rationale for the choice(s). If meta-analysis was performed, describe the model(s), method(s) to identify the presence and extent of statistical heterogeneity, and software package(s) used. | Pages 17–18 |
|  | 13e | Describe any methods used to explore possible causes of heterogeneity among study results (e.g. subgroup analysis, meta-regression). | Pages 17–18 |
|  | 13f | Describe any sensitivity analyses conducted to assess robustness of the synthesized results. | Page 19 |
| Reporting bias assessment | 14 | Describe any methods used to assess risk of bias due to missing results in a synthesis (arising from reporting biases). | Page 19 |
| Certainty assessment | 15 | Describe any methods used to assess certainty (or confidence) in the body of evidence for an outcome. | Page 20 |
| **RESULTS** | | |  |
| Study selection | 16a | Describe the results of the search and selection process, from the number of records identified in the search to the number of studies included in the review, ideally using a flow diagram. | Pages 20-21; Figure 1 |
|  | 16b | Cite studies that might appear to meet the inclusion criteria, but which were excluded, and explain why they were excluded. | Table S7 |
| Study characteristics | 17 | Cite each included study and present its characteristics. | Pages 24–26; Table 1 |
| Risk of bias in studies | 18 | Present assessments of risk of bias for each included study. | Page 27; Figure 3 |
| Results of individual studies | 19 | For all outcomes, present, for each study: (a) summary statistics for each group (where appropriate) and (b) an effect estimate and its precision (e.g. confidence/credible interval), ideally using structured tables or plots. | Table 1; Figures 4-6 |
| Results of syntheses | 20a | For each synthesis, briefly summarise the characteristics and risk of bias among contributing studies. | Pages 28–33 |
|  | 20b | Present results of all statistical syntheses conducted. If meta-analysis was done, present for each the summary estimate and its precision (e.g. confidence/credible interval) and measures of statistical heterogeneity. If comparing groups, describe the direction of the effect. | Pages 28–33 |
|  | 20c | Present results of all investigations of possible causes of heterogeneity among study results. | Pages 28–33 |
|  | 20d | Present results of all sensitivity analyses conducted to assess the robustness of the synthesized results. | Pages 33–34 |
| Reporting biases | 21 | Present assessments of risk of bias due to missing results (arising from reporting biases) for each synthesis assessed. | - |
| Certainty of evidence | 22 | Present assessments of certainty (or confidence) in the body of evidence for each outcome assessed. | Page 34; Table 2 |
| **DISCUSSION** | | |  |
| Discussion | 23a | Provide a general interpretation of the results in the context of other evidence. | Pages 36–38 |
|  | 23b | Discuss any limitations of the evidence included in the review. | Pages 38–40 |
|  | 23c | Discuss any limitations of the review processes used. | Pages 38–40 |
|  | 23d | Discuss implications of the results for practice, policy, and future research. | Pages 40-41 |
| **OTHER INFORMATION** | | |  |
| Registration and protocol | 24a | Provide registration information for the review, including register name and registration number, or state that the review was not registered. | Page 9 |
|  | 24b | Indicate where the review protocol can be accessed, or state that a protocol was not prepared. | Page 9 |
|  | 24c | Describe and explain any amendments to information provided at registration or in the protocol. | Page 9; Table S4 |
| Support | 25 | Describe sources of financial or non-financial support for the review, and the role of the funders or sponsors in the review. | Page 41; Table S22 |
| Competing interests | 26 | Declare any competing interests of review authors. | Page 42 |
| Availability of data, code and other materials | 27 | Report which of the following are publicly available and where they can be found: template data collection forms; data extracted from included studies; data used for all analyses; analytic code; any other materials used in the review. | Page 42 |

# Standardisation of isolation measurements to distance

To standardise measurements of isolation from natural habitat, we converted them to distance from nearest natural habitat in metres. Of the total 45 studies considered for inclusion, 24 reported distance as a measure of isolation, and hence only required converting to standardised units of distance (metres). Among the remaining 21 studies, 19 measured isolation using proportional area and two studies used near/far categorisation. For these studies, we attempted to use GPS locations provided by the corresponding authors (in reports or upon request) to derive the distances from nearest natural habitat via satellite imagery using the circle option in the ruler function and historical view in Google Earth Pro, estimated as close to the time of the study as possible. We followed the decisions of primary studies as to what constitutes ‘natural habitat’, which included a wide range of land cover types such as forest, agroforest, grassland and shrubland.

Whilst it was not feasible to derive accurate distances for 10 studies from satellite imagery due to challenges in reliably distinguishing natural habitats from other land covers (Table S7), distances for were successfully derived and confirmed by the corresponding authors of the original studies for the remaining 11 studies (Table S5). This made an additional 11 studies eligible for inclusion in the systematic review and meta-analysis, but also potentially introduced imprecision as a result of the less direct method of measuring distance (compared to field measurements). To assess the potential impact of this potential imprecision, we included a sensitivity analysis, adding the method for deriving the distance measure as a moderating variable (see main text).

**Table S5.** Overview of studies for which distances were successfully derived using satellite imagery.

| **Authors and year** | **Reported isolation measure** | **Estimated isolation measure** | **Year of data collection** | **Date range of available/used satellite imagery** |
| --- | --- | --- | --- | --- |
| Banks et al. (2013) and Banks et al. (2014) | Farms were classified as either ‘‘adjacent’’ (directly next to) or ‘‘isolated’’ (>100 m) from any forest habitat at least 0.5 ha in size). | Distance to nearest forest fragment | 2010 | 2010 and 2013 |
| Escobedo-Kenefic et al. (2022) | Proportion of each land use, classified as i) forest, ii) crops, iii) seminatural vegetation and iv) human settlements within a1km radius buffer. | Distance to nearest forest or stablished natural vegetation fragment | 2020 | 2020 and 2025 |
| Geeraert et al. (2020) | Proportion of tree cover within a 300m buffer around each plot, whereas this consisted mainly of coffee production forests, a few small wood lots for timber production and large single trees. | Distance to nearest forest fragment | 2018 | 2020 |
| Landaverde-Gonzalez et al. (2017) | Percentage of three specific land cover types at increasing radii (ranging from 200 – 1000m) from the centre of each sampling site: (i) forest, natural vegetation comprising trees with a height >10 m (Forest); (ii) fallow land, traditional domestic gardens surrounding dwellings, and pastures (FGP) which are an integral component of the milpa agricultural system; and (iii) cropland, mixed cropping systems at a distance from permanent settlements and with traditional and culturally important maize, beans and Habanero chilli  (all annuals) with some citrus and avocado (perennials) (Crops). | Distance to nearest shrubland or forest fragment | 2010-2011 | 2010-2014 |
| Obregon et al. (2021) | Proportion of primary and secondary forest within a 500m radius | Distance to nearest forest patch | 2018 | 2012 and 2018 |
| Schrader et al. (2018) | Categorised: pairs of home gardens surrounded by woody habitats vs. isolated from woody habitats | Distance to nearest forest patch | 2014 | 2014 |
| Sritongchuay et al. (2019) | Categorised fruit orchards “near” (<1km) and “far” (>7km) from the closest forest edge | Distance to nearest forest | 2012-2013 | 2013, 2014 and 2016 |
| Tangtorwongsakul et al. (2018) | Proportion of semi-natural habitat (e.g. grassland, pasture, old field, scrub) within 1km radius | Distance to nearest semi-natural habitat aligning with location of Figure 1 in the original publication | 2015 | 2015 |
| Vogel et al. (2021) | proportions of semi-natural habitat (mainly composed of shrubland and forest) within a 1km radius | Distance to nearest patch of forest or shrubland | 2019 | 2017 - 2019 |
| Vogel et al. (2023) | Shrubland cover within a 1km radius | Distance to nearest shrubland patch | 2019 | 2017-2023 |


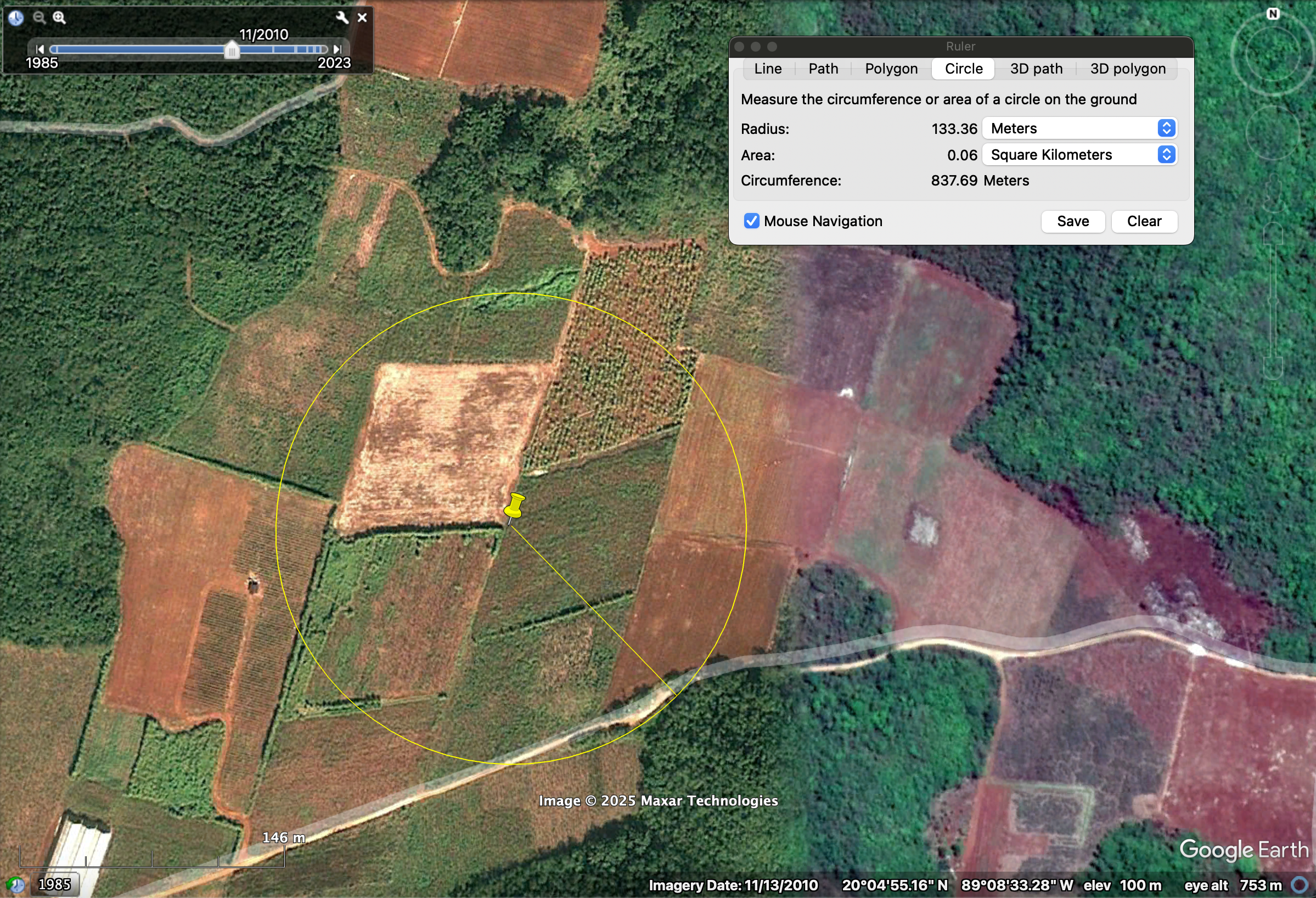

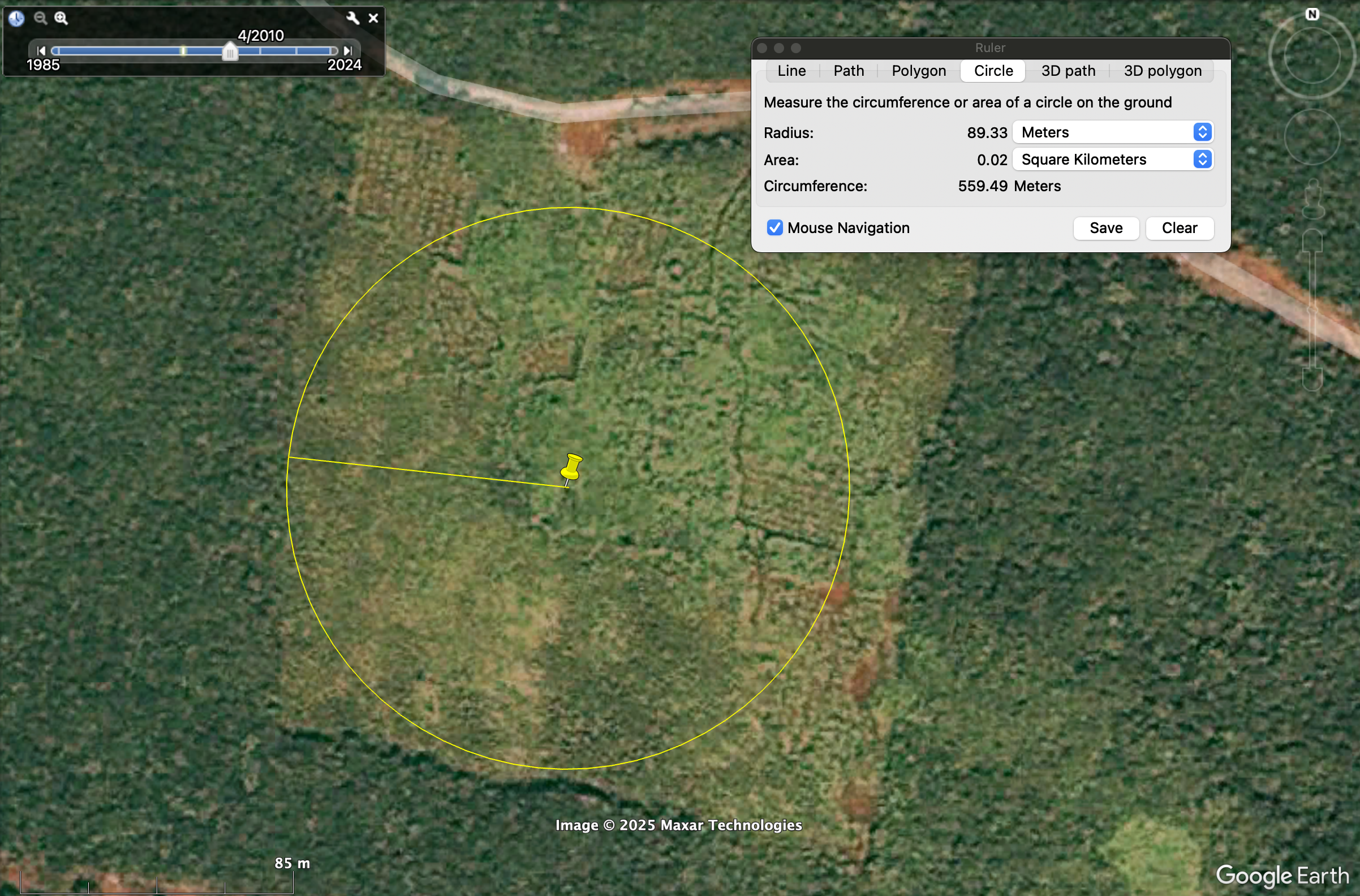


**Figure S2.** Examples of distances derived from satellite imagery for the dataset by Landaverde-Gonzalez et al. (2017) who studied farmers’ chilli fields across a gradient of forest loss. We derived distances to the nearest forest edge using historic satellite imagery as close as possible to the study’s data collection period (2010-2011).

# List of studies excluded with reason

A total of 148 studies were excluded from the meta-analysis. Of these, 122 studies did not meet our eligibility criteria (Table S6), and a further 26 studies were excluded because raw data could not be obtained (Table S7). The breakdown of exclusion reasons is as follows:

**No/wrong population (71):**

- Not insect pollinators (1)
- Not on smallholder farm (57)
- Not in tropics (13)

**No/wrong exposure/comparator (23):**

- No isolation measure (23)

**No/wrong outcome (7):**

- Different response variable (7)

**Wrong study design (4):**

- Not empirical field study (4)

**Full text not in English (8)**

**Duplicate (7)**

- Duplicate article (3)
- Same datasets already included in the meta-analysis (4)

**Retracted article (2)**

**Unable to retrieve raw data (26)**

- Unable to derive distances from satellite imagery (10)
- Unable to derive raw data from corresponding authors (16)

**Table S6.** Overview of studies excluded with reason at the full-text stage (n = 122) during the systematic review

| **#** | **Authors and year** | **Title** | **Exclusion reason** |
| --- | --- | --- | --- |
| 1 | Vansynghel et al. (2022) | Quantifying services and disservices provided by insects and vertebrates in cacao agroforestry landscapes. | Crops not pollinated by insects (n= 1) |
| 2 | Gemmill-Herren and Ochieng' 2008 | Role of native bees and natural habitats in eggplant (Solanum melongena) pollination in Kenya | Duplicate (n= 3) |
| 3 | Klein et al. (2003) | Pollination of Coffea canephora in relation to local and regional agroforestry management |  |
| 4 | Delgado-Carrillo et al. (2024) | Pollination services to crops of watermelon Citrullus lanatus and green tomato Physalis ixocarpa in the coastal region of Jalisco, Mexico. |  |
| 5 | Krishnan et al. (2018) | Pollinator services in coffee agroforests of the Western Ghats (*same dataset as Krishnan et al 2012*) | Same datasets already included in meta-analysis (n = 4) |
| 6 | Munyuli (2011) | Factors governing flower visitation patterns and quality of pollination services delivered by social and solitary bee species to coffee in central Uganda. *(same dataset as Munyuli 2012)* |  |
| 7 | (Klein et al. 2004) | Landscape context affects bee diversity and pollination in European and tropical agroecosystems |  |
| 8 | Landaverde-Gonzalez et al. (2021) | The effect of landscape on Cucurbita pepo-pollinator interaction networks varies depending on plants' genetic diversity. |  |
| 9 | Susilawati et al. (2017) | Effect of natural habitat on diversity and abundance of flower-visiting insects in cucumber fields. | Full text not in English  (n = 8) |
| 10 | Sepulveda-Cano et al. (2017) | Effect of the spatial arrangement of agroecosystem on bee (Hymenoptera: Apoidea) diversity in potato Solanum tuberosum crops of Antioquia, Colombia. |  |
| 11 | Cepeda-Valencia et al. (2014) | The structure matters: bees visitors of coffee flowers and agroecological main structure (MAS). |  |
| 12 | Flores et al. (2012) | The importance of natural habitats in the surroundings of dwarf cashew Anacardium occidentale plantation for its reproductive success. |  |
| 13 | Bravo M. et al. (2011) | Factors affecting the abundance of cocoa-pollinating midges in agroforestry systems. |  |
| 14 | Calle et al. (2010) | The production of passion fruit in Colombia: Perspectives for habitat conservation through pollinaiton services |  |
| 15 | Benevides et al. (2009) | Yellow passion fruit Passiflora edulisflavicarpa Deg. Passifloraceae) floral visitors in cultivated areas within different distances from forest remnants in north Rio de Janeiro state. |  |
| 16 | Chacon-Ortiz et al. (2023) | Evaluation of the productivity of coffee, pollinated by bees in a Venezuelan Andean agroecosystem. |  |
| 17 | Armas-Quiñonez et al. (2020) | Bee diversity in secondary forests and coffee plantations in a transition between foothills and highlands in the Guatemalan Pacific Coast | Not comparing farms with different levels of isolation from natural habitat / no isolation measure (n = 23) |
| 18 | Arnold et al. (2021) | Beneficial insects are associated with botanically rich margins with trees on small farms |  |
| 19 | Machado et al. (2024) | Optimizing coffee production: increased floral visitation and bean quality at plantation edges with wild pollinators and natural vegetation. |  |
| 20 | Maharani et al. (2024) | The diversity and ecological roles of insects and arachnids in Arabica coffee (<ovid:i>Coffea arabica</ovid:i>) plantation in Palasari, Bandung regency. |  |
| 21 | Schwarz et al. (2024) | Effects of habitat biotic features on hymenopteran diversity in East Africa. |  |
| 22 | Das et al. (2023) | Diversity of bees in two crops in an agroforestry ecosystem in Kangsabati South Forest Division, Purulia, West Bengal, India. |  |
| 23 | Vaca-Uribe et al. (2021) | Plant richness and blooming cover affect abundance of flower visitors and network structure in Colombian orchards. |  |
| 24 | Rizali et al. (2018) | Does Landscape Complexity and Semi-Natural Habitat Structure Affect Diversity of Flower-Visiting Insects in Cucumber Fields? |  |
| 25 | Gikungu et al. (2011) | Bee diversity along a forest regeneration gradient in Western Kenya |  |
| 26 | Hagen et al. (2010) | Agricultural surroundings support flower-visitor networks in an Afrotropical rain forest |  |
| 27 | Lasway et al. (2022) | Agricultural intensification with seasonal fallow land promotes high bee diversity in Afrotropical drylands |  |
| 28 | Schüepp et al. (2012) | High Bee and Wasp Diversity in a Heterogeneous Tropical Farming System Compared to Protected Forest |  |
| 29 | Serralta-Batun et al. (2024) | Taxonomic and Functional Diversity of Bees in Traditional Agroecosystems and Tropical Forest Patches on the Yucatan Peninsula |  |
| 30 | Seifert et al. (2022) | Biodiversity and Ecosystem Functions Across an Afro-Tropical Forest Biodiversity Hotspot |  |
| 31 | Simba et al. (2018) | Indirect interactions between crops and natural vegetation through flower visitors: the importance of temporal as well as spatial spillover. |  |
| 32 | Stein et al. (2018) | Impact of human disturbance on bee pollinator communities in savanna and agricultural sites in Burkina Faso, West Africa. |  |
| 33 | Ngongolo et al. (2015) | Floral Visitors and Pollinators of Sesame from Kichi Forest to the Adjacent Local Communities' Farms |  |
| 34 | Samnegard et al. (2014) | Dominance of the semi-wild honeybee as coffee pollinator across a gradient of shade-tree structure in Ethiopia. |  |
| 35 | Munyuli 2014 | Influence of functional traits on foraging behaviour and pollination efficiency of wild social and solitary bees visiting coffee Coffea canephora flowers in Uganda. |  |
| 36 | Jha et al. (2010) | Impacts of coffee agroforestry management on tropical bee communities. |  |
| 37 | Kingazi et al. 2024 | Tropical agroforestry supports insect pollinators and improves bean yield |  |
| 38 | Vides-Borrell et al. (2019) | Polycultures, pastures and monocultures: effects of land use intensity on wild bee diversity in tropical landscapes of southeastern Mexico |  |
| 39 | Young (1986) | Habitat differences in cocoa tree flowering, fruit-set, and pollinator availability in Costa Rica |  |
| 40 | Marrero et al. (2024) | Landscape heterogeneity affects pollen transport by pollinators in agroecosystems. | not in tropics (n = 13) |
| 41 | Ramirez-Mejia et al. (2023) | hummingbirds, honeybees, and wild insect pollinators affect yield and berry quality of blueberries depending on cultivar and farm's spatial context. |  |
| 42 | Hauber et al. (2022) | Mixed effect of habitat fragmentation on pollinator visitation rates but not on seed production in renosterveld of South Africa. |  |
| 43 | Monasterolo et al. (2022) | Native pollinators increase fruit set while honeybees decrease the quality of mandarins in family farms. |  |
| 44 | Mazzei et al. (2021) | Seminatural habitats and their proximity to the crop enhances canola Brassica napus pollination and reproductive parameters in Argentina. |  |
| 45 | Cunningham-Minnick et al. (2020) | Bee communities and pollination services in adjacent crop fields following flower removal in an invasive forest shrub. |  |
| 46 | Huais et al. (2020) | Forest fragments influence pollination and yield of soybean crops in Chaco landscapes. |  |
| 47 | Monasterolo et al. (2015) | Soybean crops may benefit from forest pollinators. |  |
| 48 | Greenleaf et al. (2006) | Wild bees enhance honey bees' pollination of hybrid sunflower |  |
| 49 | Astegiano et al. 2024 | Diversifying agroecological systems: Plant-pollinator network organisation and landscape heterogeneity matter |  |
| 50 | Rossi Rotondi et al. (2023) | Floral species evenness is the major driver of wild bee communities in urban gardens. |  |
| 51 | Grass et al. (2014) | Natural habitat loss and exotic plants reduce the functional diversity of flower visitors in a heterogeneous subtropical landscape |  |
| 52 | (Gonzalez et al. 2017) | Natural vegetation cover in the landscape and edge effects: differential responses of insect orders in a fragmented forest |  |
| 53 | Mulwa et al. (2022) | Influence of proximity to and type of foraging habitat on value of insect pollination in the tropics, with applications to Kenya | not observational / empirical field study  ( n = 4) |
| 54 | Samnegard 2021 | Why is arabica coffee visited by so few non-Apis bees in its native range? |  |
| 55 | (Outhwaite et al. 2022) | Availability and proximity of natural habitat influence cropland biodiversity in forest biomes globally |  |
| 56 | (Priess et al. 2007) | Linking deforestation scenarios to pollination services and economic returns in coffee agroforestry systems |  |
| 57 | Blanche et al. (2006) | Proximity to rainforest enhances pollination and fruit set in orchards. | not on smallholder farms  ( n = 57) |
| 58 | Blanche et al. (2005) | Rain forest provides pollinating beetles for atemoya crops. |  |
| 59 | Geslin et al. (2016) | Spatiotemporal changes in flying insect abundance and their functional diversity as a function of distance to natural habitats in a mass flowering crop. |  |
| 60 | Saturni et al. (2016) | Landscape structure influences bee community and coffee pollination at different spatial scales. |  |
| 61 | Hohlenwerger et al. (2024) | Coffee pollination and pest control are affected by edge diversity at local scales but multiscalar approaches and disservices can not be ignored |  |
| 62 | Anders et al. (2024) | Complementary effects of pollination and biocontrol services enable ecological intensification in macadamia orchards |  |
| 63 | Campbell et al. (2023) | Forest conservation maximises acai palm pollination services and yield in the Brazilian Amazon |  |
| 64 | Reiss-Woolever et al. (2023) | Habitat heterogeneity supports day-flying Lepidoptera in oil palm plantations |  |
| 65 | De Aguiar et al. (2015) | Changes in Orchid Bee Communities Across Forest-Agroecosystem Boundaries in Brazilian Atlantic Forest Landscapes |  |
| 66 | Masiga et al. (2014) | Do French beans Phaseolus vulgaris grown in proximity to Mt Kenya forest in Kenya experience pollination deficit? |  |
| 67 | de Sousa et al. (2022) | Natural habitat cover and fragmentation per se influence orchid-bee species richness in agricultural landscapes in the Brazilian Cerrado |  |
| 68 | Laha et al. (2020) | Exploring the importance of floral resources and functional trait compatibility for maintaining bee fauna in tropical agricultural landscapes |  |
| 69 | Livingston et al. (2013) | Conservation value and permeability of neotropical oil palm landscapes for orchid bees |  |
| 70 | Nery et al. (2018) | Bee diversity responses to forest and open areas in heterogeneous Atlantic Forest |  |
| 71 | Rosa et al. (2015) | Permeability of matrices of agricultural crops to Euglossina bees (Hymenoptera, Apidae) in the Atlantic Rain Forest |  |
| 72 | Olson et al. (2021) | Wild Bee Pollinators Foraging in Peanut and Cotton Adjacent to Native Wildflower Strips |  |
| 73 | Widhiono et al. (2016) | Insect pollinator diversity along a habitat quality gradient on Mount Slamet, Central Java, Indonesia |  |
| 74 | Bartelli et al. (2023) | Mixed-species system and native vegetation cover shape bee community in tomato crops |  |
| 75 | Anders et al. (2023) | Smart orchard design improves crop pollination. |  |
| 76 | Mohd-Azlan et al. (2023) | The filtering effect of oil palm plantations on potential insect pollinator assemblages from remnant forest patches. |  |
| 78 | Tommasi et al. (2022) | DNA metabarcoding unveils the effects of habitat fragmentation on pollinator diversity, plant-pollinator interactions, and pollination efficiency in Maldive islands. |  |
| 79 | Power et al. (2022) | Even small forest patches increase bee visits to flowers in an oil palm plantation landscape |  |
| 80 | Esquivel et al. (2021) | Crop and semi-natural habitat configuration affects diversity and abundance of native bees (Hymenoptera: Anthophila) in a large-field cotton agroecosystem. |  |
| 81 | Egonyu et al. (2021) | Insect flower-visitors of African oil palm Elaeis guineensis at different sites and distances from natural vegetation in Uganda. |  |
| 82 | Escobedo-Kenefic et al. (2020) | Disentangling the effects of local resources, landscape heterogeneity and climatic seasonality on bee diversity and plant-pollinator networks in tropical highlands. |  |
| 83 | Montoya-Pfeiffer et al. (2020) | Bee pollinator functional responses and functional effects in restored tropical forests. |  |
| 84 | Cusser et al. (2019) | Small but critical: semi-natural habitat fragments promote bee abundance in cotton agroecosystems across both Brazil and the United States. |  |
| 85 | Ramos et al. (2018) | Crop fertilization affects pollination service provision - common bean as a case study. |  |
| 86 | Halinski et al. (2018) | Influence of wild bee diversity on canola crop yields. |  |
| 87 | Grass et al. (2018) | Pollination limitation despite managed honeybees in South African macadamia orchards. |  |
| 88 | Lichtenberg et al. (2017) | Foraging traits modulate stingless bee community disassembly under forest loss. |  |
| 89 | Caudill et al. (2017) | Forest and trees: shade management, forest proximity and pollinator communities in southern Costa Rica coffee agriculture. |  |
| 90 | Franceschinelli et al. (2017) | Influence of landscape context on the abundance of native bee pollinators in tomato crops in Central Brazil. |  |
| 91 | Sritongchuay et al. (2016) | Effects of forest and cave proximity on fruit set of tree crops in tropical orchards in Southern Thailand. |  |
| 92 | Manju Devi et al. (2016) | Impact of habitat on insect pollinator diversity on coriander Coriandrum sativum bloom. |  |
| 93 | Berecha et al. (2015) | Fragmentation and management of Ethiopian moist evergreen forest drive compositional shifts of insect communities visiting wild arabica coffee flowers. |  |
| 94 | Moreira et al. (2015) | Spatial heterogeneity regulates plant-pollinator networks across multiple landscape scales. |  |
| 95 | Freitas et al. (2014) | Forest remnants enhance wild pollinator visits to cashew flowers and mitigate pollination deficit in NE Brazil. |  |
| 96 | Pires et al. (2014) | Importance of bee pollination for cotton production in conventional and organic farms in Brazil. |  |
| 97 | Witter et al. (2014) | The bee community and its relationship to canola seed production in homogenous agricultural areas. |  |
| 98 | Carvalheiro et al. (2011) | Natural and within-farmland biodiversity enhances crop productivity. |  |
| 99 | Wojcik (2011) | Resource abundance and distribution drive bee visitation within developing tropical urban landscapes. |  |
| 100 | Carvalheiro et al. (2010) | Pollination services decline with distance from natural habitat even in biodiversity-rich areas. |  |
| 101 | Brosi et al. (2009) | The complex responses of social stingless bees (Apidae: Meliponini) to tropical deforestation. |  |
| 102 | Chacoff et al. (2008) | Proximity to forest edge does not affect crop production despite pollen limitation |  |
| 103 | Brosi et al. (2007) | Bee community shifts with landscape context in a tropical countryside. |  |
| 104 | Chacoff and Aizen (2006) | Edge effects on flower-visiting insects in grapefruit plantations bordering premontane subtropical forest. |  |
| 105 | Ricketts (2004) | Tropical forest fragments enhance pollinator activity in nearby coffee crops. |  |
| 106 | Finger et al. (2021) | Arthropods at the interface between monoculture and native forest. |  |
| 107 | Gonzalez-Chaves et al. (2020) | Forest proximity rather than local forest cover affects bee diversity and coffee pollination services. |  |
| 108 | Ferreira et al. (2022) | Critical role of native forest and savannah habitats in retaining neotropical pollinator diversity in highly mechanized agricultural landscapes. |  |
| 109 | Tarigan et al. (2021) | Agroforestry inside oil palm plantation for enhancing biodiversity-based ecosystem functions. |  |
| 110 | Munoz et al. (2021) | Native flowering shrubs promote beneficial insects in avocado orchards. |  |
| 111 | Almeida et al. (2020) | Contribution of the Cerrado as habitat for sunflower pollinating bees. |  |
| 112 | Adjaloo et al. (2012) | Pollinator status of bees in cocoa agro-ecological system. |  |
| 113 | Hoehn et al. (2008) | Functional group diversity of bee pollinators increases crop yield. |  |
| 114 | Farfan et al. (2023) | The effect of landscape composition on stingless bee Melipona fasciculata honey productivity in a wetland ecosystem of Eastern Amazon, Brazil. | Different response variable (neither pollinator abundance, richness or fruit set) (n = 7) |
| 115 | Munyuli (2012) | Butterfly diversity from farmlands of central Uganda. |  |
| 116 | (González-Chaves et al. 2023) | Evidence of time-lag in the provision of ecosystem services by tropical regenerating forests to coffee yields |  |
| 117 | (Jeronimo and Varassin 2023) | Like an “espresso” but not like a “cappuccino”: landscape metrics are useful for predicting coffee production at the farm level but not at the municipality level |  |
| 118 | (Lima and Mariano Neto 2014) | Extinction thresholds for Sapotaceae due to forest cover in Atlantic Forest landscapes |  |
| 119 | Balachandran et al. (2017) | Pollinator diversity and foraging dynamics on monsoon crop of cucurbits in a traditional landscape of South Indian west coast |  |
| 120 | Medeiros et al. (2019) | Landscape structure shapes the diversity of beneficial insects in coffee producing landscapes. |  |
| 121 | Munyuli (2013) | Climatic, regional land-use intensity, landscape, and local variables predicting best the occurrence and distribution of bee community diversity in various farmland habitats in Uganda | Retracted article (n = 2) |
| 122 | Munyuli et al. (2013) | Patterns of bee diversity in mosaic agricultural landscapes of central Uganda: implication of pollination services conservation for food security. |  |

**Table S7.** Overview of studies excluded because we were unable to obtain data for meta-analysis (n = 26)

| 1 | Carneiro et al. (2021) | The Interplay Between Thematic Resolution, Forest Cover, and Heterogeneity for Explaining Euglossini Bees Community in an Agricultural Landscape | Unable to derive distances from satellite imagery ( n = 10) |
| --- | --- | --- | --- |
| 2 | Boakye et al. (2024) | Relationships between flower-visiting insects and forest cover in cocoa-growing landscapes in Ghana |  |
| 3 | Tommasi et al. (2021) | Impact of land use intensification and local features on plants and pollinators in sub-Saharan smallholder farms. |  |
| 4 | Assuncao et al. (2022) | Landscape conservation and local interactions with non-crop plants aid in structuring bee assemblages in organic tropical agroecosystems. |  |
| 5 | Otieno et al. (2015) | Local and landscape effects on bee functional guilds in pigeon pea crops in Kenya |  |
| 6 | Struelens et al. (2021) | Combined effects of landscape composition and pesticide use on herbivore and pollinator functions in smallholder farms |  |
| 7 | Marcacci et al. (2022) | Functional diversity of farmland bees across rural-urban landscapes in a tropical megacity |  |
| 8 | Simla et al. (2022) | Effect of landscape composition and invasive plants on pollination networks of smallholder orchards in northeastern Thailand |  |
| 9 | Campbell et al. (2018) | Anthropogenic disturbance of tropical forests threatens pollination services to acai palm in the Amazon river delta. |  |
| 10 | (Ferreira et al. 2024) | Landscape forest cover and regional context shape the conservation value of shaded cocoa agroforests for bees and social wasps |  |
| 11 | Delaney et al. (2020) | Local-scale tree and shrub diversity improves pollination services to shea trees in tropical west African parklands. | Unable to derive raw data (n = 16) |
| 12 | Karnchananiyom et al. (2023) | Local and landscape context affects bee communities in mixed fruit orchards in southern Thailand. |  |
| 13 | Cely-Santos et al. (2019) | Local and landscape habitat influences on bee diversity in agricultural landscapes in Anolaima, Colombia |  |
| 14 | Buchori et al. (2019) | Natural habitat fragments obscured the distance effect on maintaining the diversity of insect pollinators and crop productivity in tropical agricultural landscapes |  |
| 15 | Silva et al. (2019) | Isolation from natural habitat reduces yield and quality of passion fruit |  |
| 16 | Bravo-Monroy et al. (2015) | Ecological and social drivers of coffee pollination in Santander, Colombia |  |
| 17 | Arnob Chatterjee et al. (2020) | Predicted thresholds for natural vegetation cover to safeguard pollinator services in agricultural landscapes. |  |
| 18 | Delgado-Carrillo et al. (2024) | Pollination services to crops of watermelon (Citrullus lanatus) and green tomato (Physalis ixocarpa) in the coastal region of Jalisco, Mexico |  |
| 19 | Samnegard et al. (2016) | A heterogeneous landscape does not guarantee high crop pollination. |  |
| 20 | Samnegard et al. (2015) | Turnover in bee species composition and functional trait distributions between seasons in a tropical agricultural landscape. |  |
| 21 | Otieno et al. (2011) | Local management and landscape drivers of pollination and biological control services in a Kenyan agro-ecosystem. |  |
| 22 | Boreux et al. (2013) | Impact of forest fragments on bee visits and fruit set in rain-fed and irrigated coffee agro-forests. |  |
| 23 | Sritongchuay et al. (2017) | Effects of forest proximity on fruit set and visitor body size of Sandoricum koetjape (Burm.f.) Merr. in Southern Thailand |  |
| 24 | Martins and Johnson (2009) | Distance and quality of natural habitat influence hawkmoth pollination of cultivated papaya |  |
| 25 | Vansynghel et al. (2022) | Cacao flower visitation: lowpollen deposition, low fruit set and dominance of herbivores |  |
| 26 | Chiawo et al. (2017) | Bee diversity and floral resources along a disturbance gradient in Kaya Muhaka forest and surrounding farmlands of coastal Kenya. |  |

# Study design types

We grouped the studies included in our meta-analysis into three broad study designs: (A) *single-distance-per-site*, (B) *nested distances*, and (C) *paired sites* (Fig S3). Study-level effect sizes were estimated using slightly different approaches depending on the study design, with site identity included as a random effect for nested and paired designs. When repeat measures were taken at the same site-distance combination, values were aggregated to a single data point (cumulative abundance and richness; mean for fruit set). Table 1 in the main manuscript provides an overview of which studies employed each design.

1. **Single-distance per site design (N=27):**

Each site was sampled at a single distance from the nearest natural habitat. Repeat measures were aggregated to one value per site. For example, a study with 24 farms contributed 24 aggregated observations of pollinator variables. The dataset from Li et al. (2022) had a slightly different unit of analysis as the study focused on Arthropods, where all data was collected on a 1.5 ha smallholder farm and the distance to the nearest natural habitat was measured for each individual palm tree. We treated this dataset as a single-distance per site design.

1. **Nested distances (N = 6):**

Multiple distances were sampled within each site, usually along transects (e.g., 0, 100, 250, 500 m). Repeat measures at the same distance were aggregated, and site identity was included as a random effect to account for non-independence. The dataset from Escobedo-Kenefic et al. (2024) had farms nested within broader locations; the number of data points equalled the number sites multiplied by the number of nested distances per site. For the Gemmill-Herren and Ochieng (2008) dataset, the richness response had identical observations within sites, causing singularity issues in GLMMs; a GLM was used instead.

1. **Paired sites:**

Farms were sampled in matched near-far pairs relative to natural habitat, with one distance per farm. Repeat measures were aggregated per farm. Pair ID or study location was included as a random effect to preserve the matched design while allowing robust estimation of landscape effects.

# Risk of bias assessment

**Study parameters for risk of bias assessment**

Prior to conducting the RoB assessment following the [CEE Critical Appraisal Tool – Environmental Evidence](https://environmentalevidence.org/cee-critical-appraisal-tool/) (Konno et al. 2021), we defined the review scope in terms of question, population, exposure and comparator group, outcome, as well as all potential confounding factors across all individual studies. This helps to ensure that the framework for conducting the RoB assessment is well-defined, and that the assessment can be carried out systematically with minimal subjective bias.

**Review question:** How does increasing distance from natural vegetation affect insect pollinator abundance, richness, and fruit set of crops in tropical small-scale farms?

**Population:** The insect pollinator (and for fruit set; the crop/plant) communities located in the smallholder farms across varying distances to the nearest natural habitat.

**Exposure/comparator:** Exposure to natural habitat, with degree of exposure decreasing with increasing distance to nearest natural habitat

**Outcome:** Response variables are insect pollinator abundance, richness, and fruit set

**List of all potential confounding factors:**

- Natural habitat type
- Local farm management (pesticide use)
- Farm size
- Floral resource availability
- Surrounding land cover heterogeneity
- Season (rainy vs wet season)
- Weather (e.g. observations only on sunny days)
- Time of the day for insect observations
- Managed honeybees

Specifically for fruit set (additional):

- Soil factors (water and nutrient availability)
- Crop pollinator-dependency

**Justification and explanation of risk classification approach**

The risk of bias for each individual study was assessed using six criteria for observational studies from the CEECAT tool, following the checklist of version 3.0. However, applying the tool presented several challenges. Many criteria were difficult to assess due to limited reporting in the primary studies, and the tool was not always well aligned with our raw data meta-analytic approach, leading to ambiguity in several judgements. Together with some inherent subjectivity in interpreting certain criteria, this limited the tool’s flexibility and precluded a formal sensitivity analysis.

1. **Confounding bias**: This criterion evaluates the risk that the study’s results are influenced by confounding factors that were not adequately controlled for or addressed. Given the complexities of ecological field studies, it is often challenging to control for, or reduce the impact of, all potential confounders. Studies were categorised as medium risk of bias if they attempted to account for at least half of the critical confounding variables listed above. Studies that addressed fewer key confounders were classified as high risk. Risk levels were downgraded if authors justified non-inclusion of confounders, e.g. study design or statistical reasons for not doing so. This approach acknowledges the inherent difficulties in controlling for confounders in ecological research while recognising efforts made by researchers to mitigate this bias.
2. **Risk of exposure selection bias**: This criterion assesses the risk that the selection of study sites could introduce bias by failing to ensure that the exposure groups (e.g., farms near vs. far from forest) are comparable. Risk was assessed based on the study site selection criteria, e.g., farms with similar environmental context such as elevation and climate, land management practices (pest control, use of agrochemicals, monoculture vs diverse crop composition/farming systems) and farm sizes. As in the context of the research question and the ecological field studies, it is generally likely that the researchers are aware of the study site classification, especially the ones where farms near vs far from forest were compared, this introduced a risk of bias for many of the studies.
3. **Risk of misclassified comparison bias**: This criterion evaluates the risk of measurement bias for the predictor variable, i.e. risk that inaccuracies or inconsistencies in how exposure/comparison (i.e., varying distance to nearest natural habitat) is classified could lead to biased results. We assigned studies low risk if they provided clear definitions and justified spatial scales for the distance to natural habitat aligned with the focal pollinator taxa (e.g., 500m to 2km for bee studies). Conversely, studies that lacked clarity in their classifications or used scales that seemed inappropriate for the focal pollinator taxa were classified as medium to high risk. Minimum distances between sites/farms, here 1km, was taken into account as an indication of risk of non-independence.
4. **Risk of detection bias**: This criterion focuses on the potential measurement bias for the response variables, specifically whether the methods used to assess outcome measures like pollinator abundance and visitation rates were consistent and well-defined. Studies were assigned medium to high risk if they did not clearly define what constitutes a 'pollinator' or did not use a standardised protocol across different sites and distances from natural habitats. Also, we considered whether studies abided by standardised/best practice approach for pollinator identification and classification of different pollinator groups. While it is likely that researchers in the field collecting data were likely aware of the exposure (proximity to natural habitat), we evaluated the likelihood that the lab and/or the process of identification of pollinators was done blinded. Studies that implemented a clear and consistent methodology were considered to have a lower risk of detection bias.
5. **Risk of outcome reporting bias**: This criterion evaluates the risk that a study selectively reports outcomes, particularly focusing on significant results while potentially omitting non-significant findings. High risk of outcome reporting bias was assigned if there was evidence that significant results were selectively reported while non-significant findings were omitted.
6. **Risk of outcome assessment bias**: This criterion evaluates the risk that statistical methods used could introduce bias in the findings. Many studies showed at least a medium risk of bias due to the potential influence of researchers' awareness of site classifications on their assessments. Studies that used relatively small, likely underpowered sample sizes were classified as high risk, reflecting concerns about the validity of their inferential conclusions.

# Meta-analysis

## Pollinator abundance

**Figure S3.** Data and model fits for the relationship between pollinator abundance and linear distance to the nearest natural habitat of each study using either GLMs or GLMMs with negative binomial error distribution (N = 31 studies; modelling approach indicated in the title of each plot).


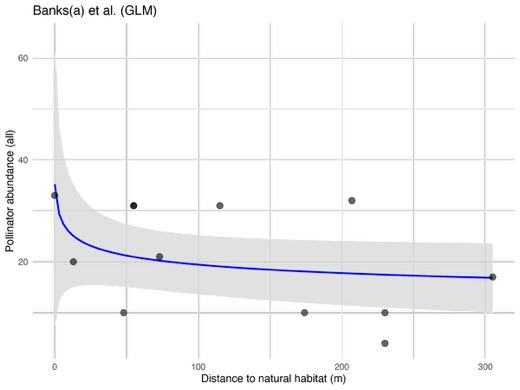

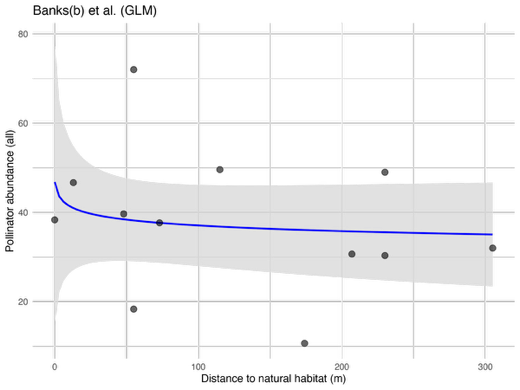

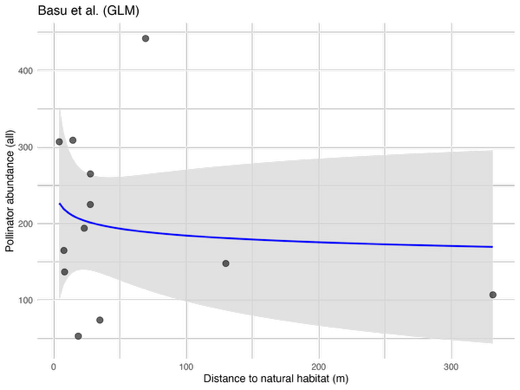

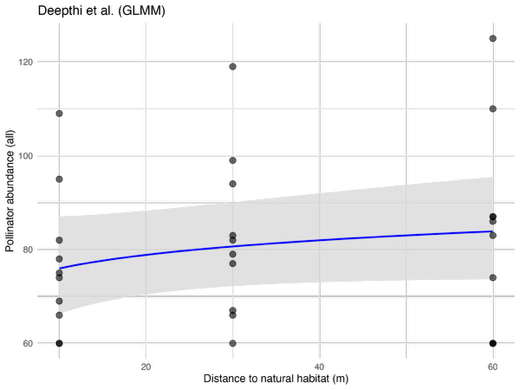

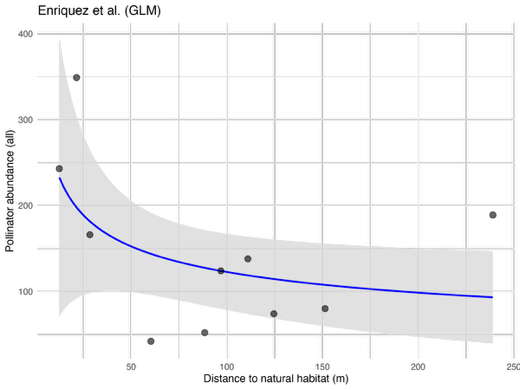

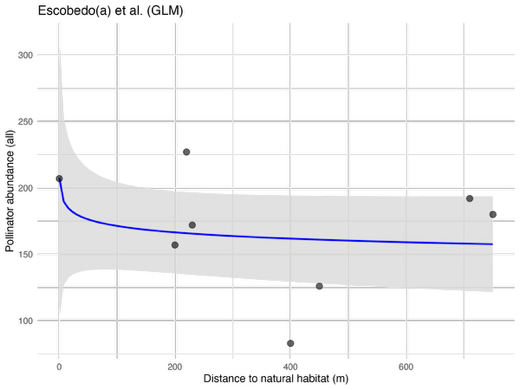

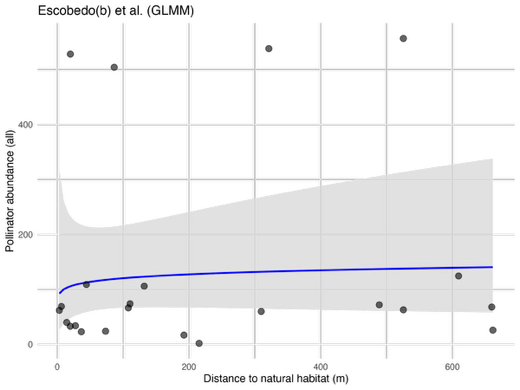

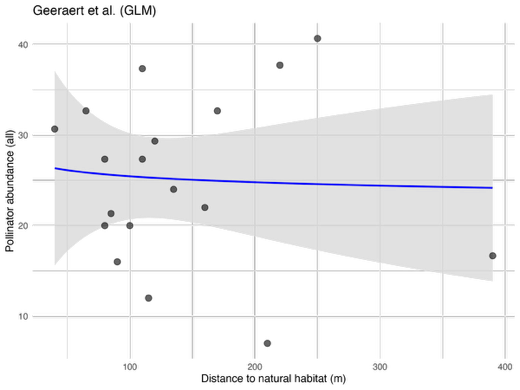

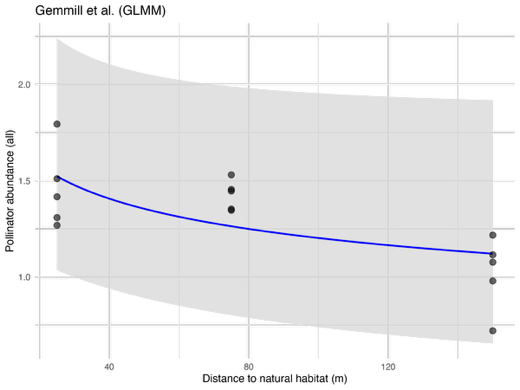

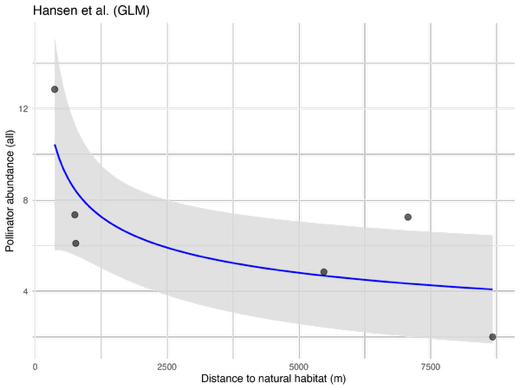

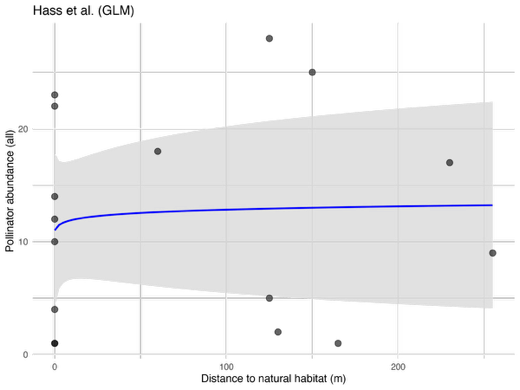

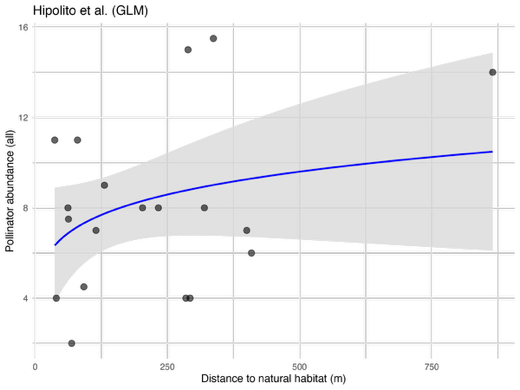

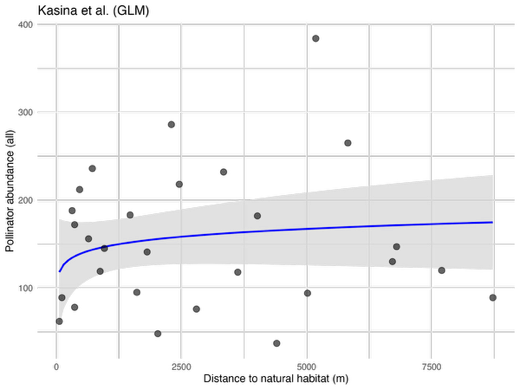

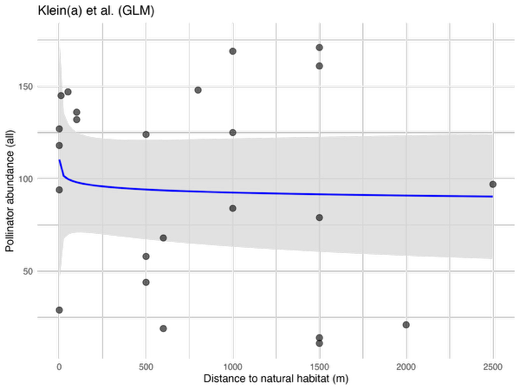

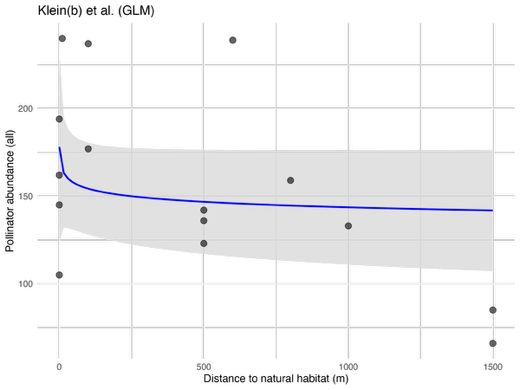

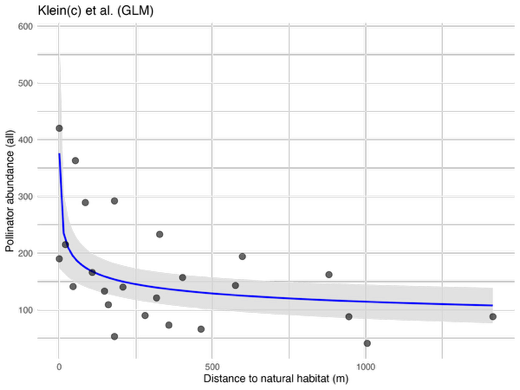

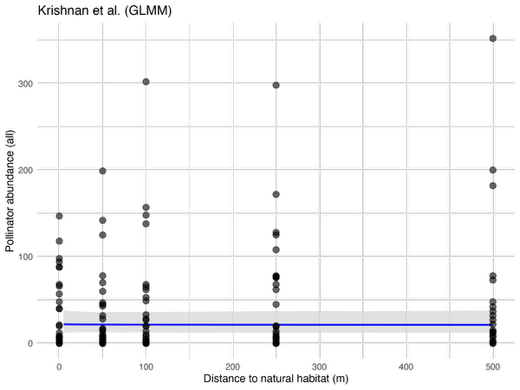

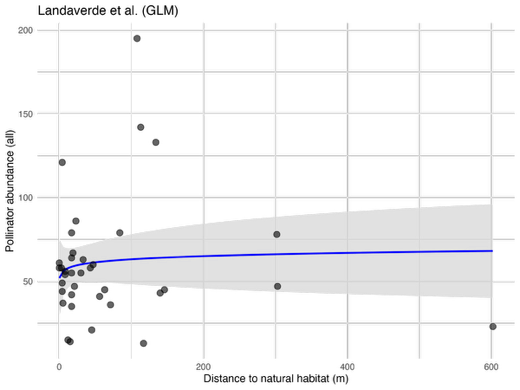

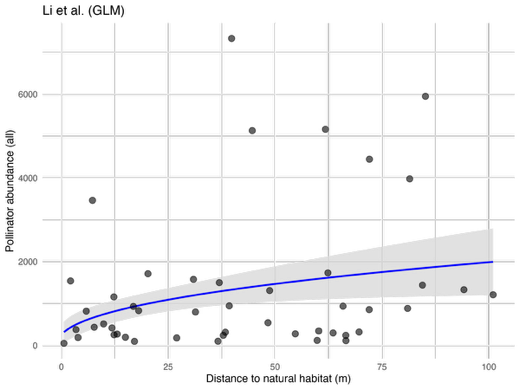

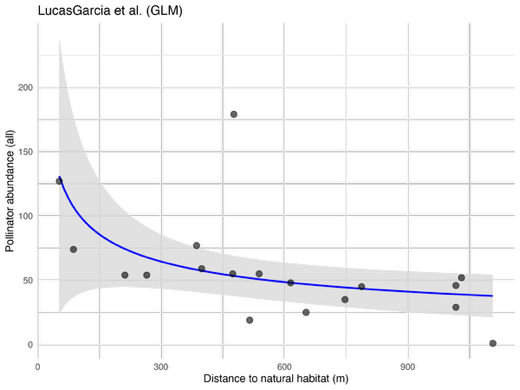

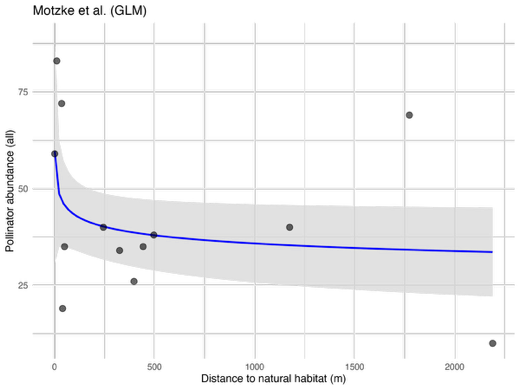

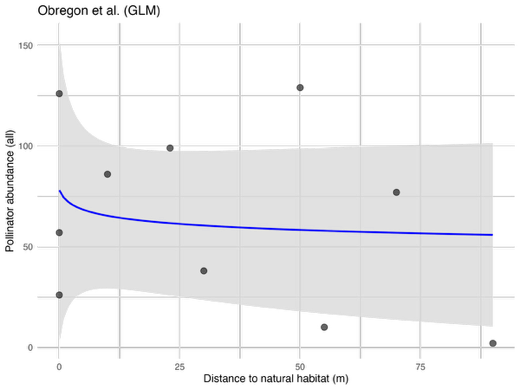

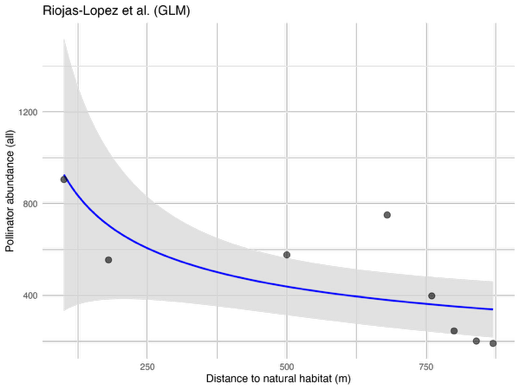

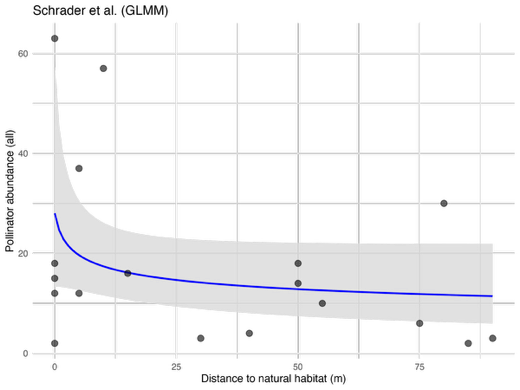

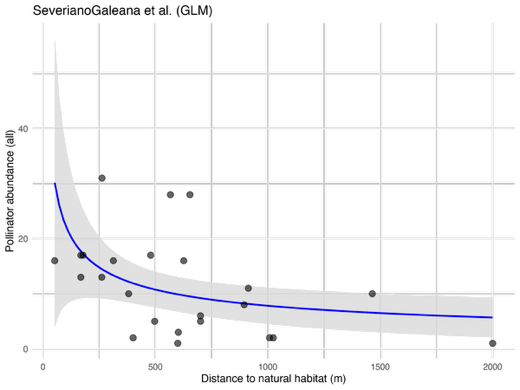

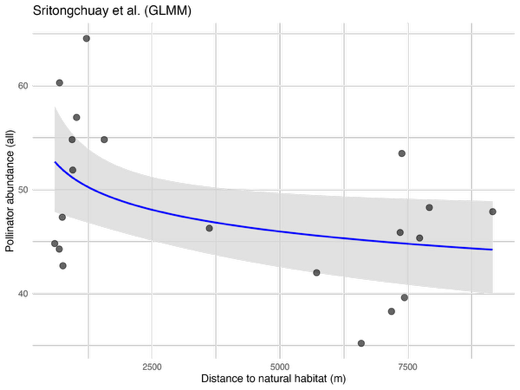

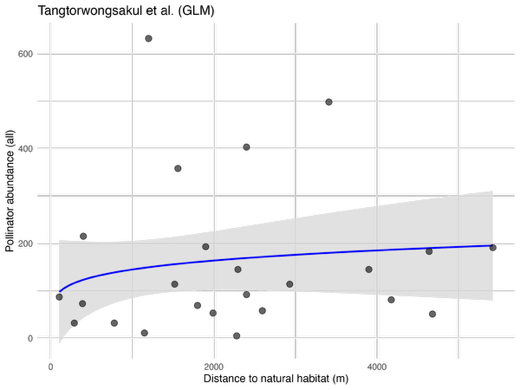

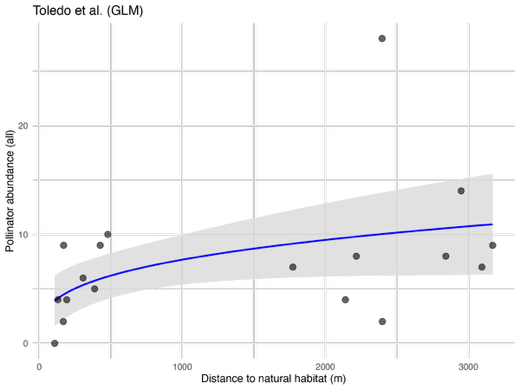

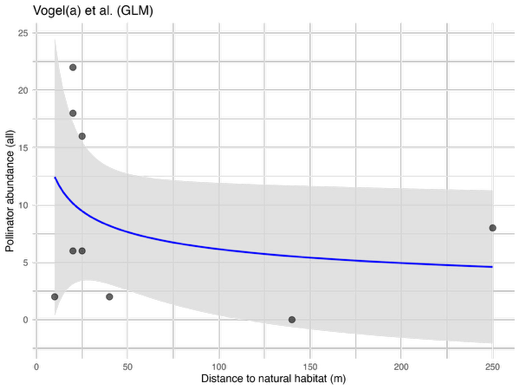

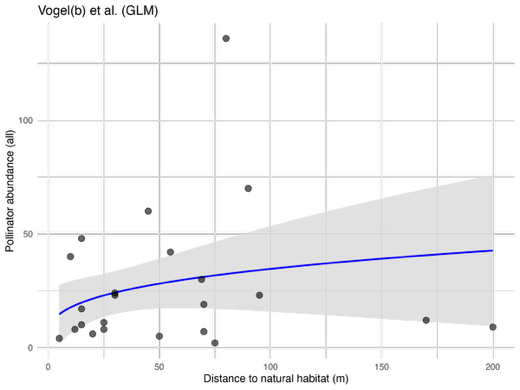

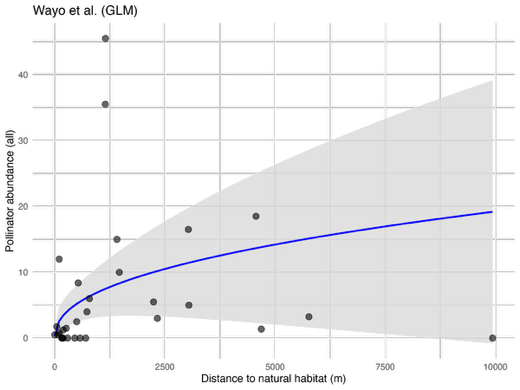


**Table S8.** Effect sizes and moderator variables for the meta-analysis on pollinator abundance (N = 31 studies)

| **Authors** | **Slope** | **StdError** | **PValue** | **Agr Intensity** | **Sites** | **Design** | **Habitat** | **Pollinator** | **Method** | **Distance Measure** | **Max Distance** |
| --- | --- | --- | --- | --- | --- | --- | --- | --- | --- | --- | --- |
| Banks et al. 2013 | -0.13 | 0.09 | 0.16 | high | 12 | single distance per site | natural forest | Hymenoptera | active | estimated | 305 |
| Banks et al. 2014 | -0.05 | 0.08 | 0.52 | high | 12 | single distance per site | natural forest | Hymenoptera | active | estimated | 305 |
| Basu et al. 2016 | -0.07 | 0.14 | 0.62 | both | 12 | single distance per site | other | Bees | passive | reported | 331 |
| Deepthi et al 2019 | 0.06 | 0.04 | 0.19 | high | 10 | nested design | natural forest | Bees | active | reported | 60 |
| Enriquez et al. 2015 | -0.32 | 0.19 | 0.10 | low | 10 | single distance per site | other | Bees | active | reported | 239 |
| Escobedo-Kenefic et al. 2022 | -0.04 | 0.05 | 0.37 | high | 8 | single distance per site | other | Insecta | active | estimated | 750 |
| Escobedo-Kenefic et al. 2024 | 0.08 | 0.17 | 0.64 | both | 6 | nested design | natural forest | Insecta | active | reported | 661 |
| Geeraert et al. 2020 | -0.04 | 0.17 | 0.82 | low | 15 | single distance per site | other | Bees | passive | estimated | 390 |
| Gemmill-Herren and Ochieng' 2008 | -0.17 | 0.10 | 0.08 | high | 15 | nested design | natural forest | Bees | active | reported | 150 |
| Hansen et al. 2020 | -0.30 | 0.13 | 0.02 | high | 6 | single distance per site | natural forest | Insecta | active | reported | 8676 |
| Hass et al. 2018 | 0.03 | 0.09 | 0.71 | high | 16 | single distance per site | other | Bees | active | reported | 255 |
| Hipolito et al. 2019 | 0.16 | 0.12 | 0.17 | both | 19 | single distance per site | other | Insecta | active | reported | 865 |
| Kasina et al. 2009 | 0.08 | 0.07 | 0.29 | low | 28 | single distance per site | natural forest | Bees | active | reported | 8721 |
| Klein et al. 2003 a) | -0.03 | 0.05 | 0.62 | low | 24 | single distance per site | natural forest | Bees | active | reported | 2500 |
| Klein et al. 2003 b) | -0.03 | 0.03 | 0.30 | low | 15 | single distance per site | natural forest | Bees | active | reported | 1500 |
| Klein 2009 | -0.17 | 0.05 | 0.00 | low | 24 | single distance per site | natural forest | Bees | active | reported | 1415 |
| Krishnan et al. 2012 | -0.01 | 0.05 | 0.91 | NA | 35 | nested design | natural forest | Bees | active | reported | 500 |
| Landaverde-Gonzales et al. 2017 | 0.04 | 0.06 | 0.49 | high | 37 | single distance per site | other | Bees | mixed | estimated | 602 |
| Li et al. 2022 | 0.44 | 0.13 | 0.00 | high | 47 | single distance per site | natural forest | Arthropods | passive | reported | 101 |
| Lucas-Garcia and Rosas-Guerrero, unpub | -0.41 | 0.18 | 0.02 | high | 18 | single distance per site | natural forest | Insecta | active | reported | 1107 |
| Motzke et al. 2016 | -0.08 | 0.05 | 0.12 | NA | 12 | single distance per site | natural forest | Bees | active | reported | 2190 |
| Obregon et al. | -0.07 | 0.16 | 0.64 | high | 10 | single distance per site | natural forest | Bees | active | estimated | 90 |
| Riojas-Lopez et al. 2019 | -0.47 | 0.19 | 0.01 | low | 8 | single distance per site | other | Bees | passive | reported | 870 |
| Schrader et al. 2018 | -0.20 | 0.13 | 0.12 | low | 10 | paired sites | other | Bees | active | estimated | 90 |
| Severiano-Galeana et al. 2024 | -0.46 | 0.19 | 0.02 | high | 24 | single distance per site | natural forest | Insecta | active | reported | 2000 |
| Sritongchuay et al. 2019 | -0.06 | 0.03 | 0.02 | low | 20 | paired sites | natural forest | Insecta | active | estimated | 9160 |
| Tangtorwongsakul et al. 2017 | 0.18 | 0.20 | 0.37 | high | 24 | single distance per site | other | Bees | mixed | estimated | 5420 |
| Toledo-Hernandez et al. 2021 | 0.31 | 0.12 | 0.01 | low | 18 | single distance per site | other | Diptera | passive | reported | 3169 |
| Vogel et al. 2021 | -0.32 | 0.34 | 0.34 | low | 9 | single distance per site | other | Bees | active | estimated | 250 |
| Vogel et al. 2023 | 0.30 | 0.21 | 0.16 | low | 24 | single distance per site | other | Insecta | active | estimated | 200 |
| Wayo et al. 2020 | 0.44 | 0.17 | 0.01 | low | 30 | single distance per site | natural forest | Stingless Bees | active | reported | 9937 |

**Table S9.** Overview of the model outputs for the main abundance model, wild abundance model, two moderator models (habitat type and agricultural intensity), and the three sensitivity models (pollinator sampling method, distance measure, and maximum distance scales). Sample size (N) for each model or category is provided. Model outputs include the estimated effect sizes, standard errors (SE), z-values, p-values and 95% confidence intervals. Significance codes: * p < 0.05; ** p < 0.01.

| **model** | **N** | **category** | **estimate** | **se** | **zval** | **pval** | **ci.lb** | **ci.ub** |
| --- | --- | --- | --- | --- | --- | --- | --- | --- |
| **Main model:**  rma(yi = Slope, vi = Variance, data = abundance_es) | 31 | - | -0.0296 | 0.0296 | -1.0023 | 0.3162 | -0.0876 | 0.0283 |
| **Wild abundance model:**  rma(yi = Slope, vi = Variance, data = results_wild) | 28 | - | -0.0536 | 0.0384 | -1.398 | 0.1621 | -0.1288 | 0.0216 |
| **Moderator model 1**  rma(Slope, Variance, mods = ~ 0 + Habitat, data=abundance_es) | 18 | natural forest | -0.0425 | 0.0371 | -1.1449 | 0.2523 | -0.1152 | 0.0303 |
|  | 13 | other | -0.005 | 0.0517 | -0.0962 | 0.9233 | -0.1064 | 0.0964 |
| **Moderator model 2**  rma(Slope, Variance, mods = ~ 0 + AgrIntensity, data=abundance_es) | 3 | both | 0.0616 | 0.1208 | 0.5103 | 0.6098 | -0.1751 | 0.2983 |
|  | 13 | high | -0.0481 | 0.0526 | -0.9148 | 0.3603 | -0.1512 | 0.055 |
|  | 13 | low | -0.0276 | 0.0545 | -0.5063 | 0.6126 | -0.1343 | 0.0792 |
| **Sensitivity model 1**  rma(Slope, Variance, mods = ~ 0 + Method, data=abundance_es) | 24 | active | -0.0537 | 0.0302 | -1.7803 | 0.075 | -0.1129 | 0.0054 . |
|  | 2 | mixed | 0.0745 | 0.1097 | 0.6789 | 0.4972 | -0.1405 | 0.2895 |
|  | 5 | passive | 0.0929 | 0.082 | 1.1326 | 0.2574 | -0.0678 | 0.2536 |
| **Sensitivity model 2**  rma(Slope, Variance, mods = ~ 0 + DistanceMeasure, data=abundance_es) | 11 | estimated | -0.0391 | 0.053 | -0.7376 | 0.4608 | -0.143 | 0.0648 |
|  | 20 | reported | -0.0252 | 0.0377 | -0.6671 | 0.5047 | -0.0991 | 0.0488 |
| **Sensitivity model 3**  rma(Slope, Variance, mods = ~ 0 + DistanceCategory, data=abundance_es) | 16 | small (<750m) | -0.013 | 0.044 | -0.2948 | 0.7682 | -0.0992 | 0.0733 |
|  | 9 | medium  (750m-3000m) | -0.1089 | 0.0528 | -2.0623 | 0.0392 | -0.2124 | -0.0054 * |
|  | 6 | large (>3000m) | 0.0663 | 0.0699 | 0.9489 | 0.3427 | -0.0706 | 0.2032 |

**Table S10.** Results of the heterogeneity analysis for the main model, wild abundance model, moderator model (agricultural intensity), and the three sensitivity models (pollinator sampling method, distance measure, and maximum distance scales). Heterogeneity measures: tau^2^ = estimated amount of residual heterogeneity; tau = square root of estimated tau^2^ value; I^2^ = residual heterogeneity / unaccounted variability; H^2^ = unaccounted variability / sampling variability; Q test = Test for Heterogeneity; For moderator analyses: QE = Test for residual Heterogeneity, QM = Test of Moderators

| **model** | **tau^2^** | **tau** | **I^2^** | **H^2^** | **Q statistic** | **p-val** |
| --- | --- | --- | --- | --- | --- | --- |
| **Main model:**  rma(yi = Slope, vi = Variance, data = abundance_es) | 0.0151 (SE = 0.0065) | 0.1231 | 74.70% | 3.95 | Q(df = 30) = 80.6203 | < .0001 |
| **Wild abundance model:**  rma(yi = Slope, vi = Variance, data = results_wild) | 0.0242 (SE = 0.0103) | 0.1554 | 78.64% | 4.68 | Q(df = 27) = 81.1340 | < .0001 |
| **Moderator model 1**  rma(Slope, Variance, mods = ~ 0 + Habitat, data=abundance_es) | 0.0161 (SE = 0.0069) | 0.1267 | 75.29% | 4.05 | QE(df = 29) = 78.9812  QM(df = 2) = 1.3200 | < .0001  0.5169 |
| **Moderator model 2**  rma(Slope, Variance, mods = ~ 0 + AgrIntensity, data=abundance_es) | 0.0235 (SE = 0.0100) | 0.1534 | 80.32% | 5.08 | QE(df = 26) = 76.3475  QM(df = 3) = 1.3536 | < .0001  0.7164 |
| **Sensitivity model 1**  rma(Slope, Variance, mods = ~ 0 + Method, data=abundance_es) | 0.0120 (SE = 0.0057) | 0.1096 | 70.28% | 3.37 | QE(df = 28) = 71.8652  QM(df = 3) = 4.9133 | < .0001  0.1783 |
| **Sensitivity model 2**  rma(Slope, Variance, mods = ~ 0 + DistanceMeasure, data=abundance_es) | 0.0170 (SE = 0.0072) | 0.1304 | 75.13% | 4.02 | QE(df = 29) = 79.7930  QM(df = 2) = 0.9890 | < .0001  0.6099 |
| **Sensitivity model 3**  rma(Slope, Variance, mods = ~ 0 + DistanceCategory, data=abundance_es) | 0.0165 (SE = 0.0073) | 0.1285 | 72.77% | 3.67 | QE(df = 28) = 75.0445  QM(df = 3) = 5.2402 | < .0001  0.1550 |

**Table S11.** Effect sizes and moderator variables used for the meta-analysis on abundance of wild pollinators, excluding managed honeybees (N= 28 studies).

| **Authors** | **Slope** | **StdError** | **PValue** | **Agr Intensity** | **Sites** | **Design** | **Habitat** | **Pollinator** | **Method** | **Distance Measure** | **Max Distance** |
| --- | --- | --- | --- | --- | --- | --- | --- | --- | --- | --- | --- |
| Banks et al. 2013 | -0.14 | 0.09 | 0.13 | high | 12 | single distance per site | natural forest | Hymenoptera | active | estimated | 305 |
| Banks et al. 2014 | -0.08 | 0.05 | 0.14 | high | 12 | single distance per site | natural forest | Hymenoptera | active | estimated | 305 |
| Basu et al. 2016 | -0.07 | 0.15 | 0.61 | both | 12 | single distance per site | other | Bees | passive | reported | 331 |
| Deepthi et al 2019 | 0.06 | 0.04 | 0.19 | high | 10 | nested design | natural forest | Bees | active | reported | 60 |
| Enriquez et al. 2015 | -0.06 | 0.25 | 0.80 | low | 10 | single distance per site | other | Bees | active | reported | 239 |
| Escobedo-Kenefic et al. 2022 | -0.01 | 0.06 | 0.91 | high | 8 | single distance per site | other | Insecta | active | estimated | 750 |
| Escobedo-Kenefic et al. 2024 | 0.08 | 0.27 | 0.76 | both | 6 | nested design | natural forest | Insecta | active | reported | 661 |
| Geeraert et al. 2020 | -0.28 | 0.17 | 0.11 | low | 15 | single distance per site | other | Bees | passive | estimated | 390 |
| Gemmill-Herren and Ochieng' 2008 | -0.17 | 0.10 | 0.08 | high | 15 | nested design | natural forest | Bees | active | reported | 150 |
| Hansen et al. 2020 | -1.38 | 0.39 | 0.00 | high | 6 | single distance per site | natural forest | Insecta | active | reported | 8676 |
| Hass et al. 2018 | 0.03 | 0.09 | 0.71 | high | 16 | single distance per site | other | Bees | active | reported | 255 |
| Kasina et al. 2009 | -0.11 | 0.08 | 0.20 | low | 28 | single distance per site | natural forest | Bees | active | reported | 8721 |
| Klein et al. 2003 a) | -0.03 | 0.05 | 0.62 | low | 24 | single distance per site | natural forest | Bees | active | reported | 2500 |
| Klein et al. 2003 b) | -0.03 | 0.03 | 0.30 | low | 15 | single distance per site | natural forest | Bees | active | reported | 1500 |
| Klein 2009 | -0.17 | 0.05 | 0.00 | low | 24 | single distance per site | natural forest | Bees | active | reported | 1415 |
| Krishnan et al. 2012 | -0.02 | 0.06 | 0.69 | NA | 35 | nested design | natural forest | Bees | active | reported | 500 |
| Landaverde-Gonzales et al. 2017 | 0.04 | 0.06 | 0.49 | high | 37 | single distance per site | other | Bees | mixed | estimated | 602 |
| Li et al. 2022 | 0.44 | 0.13 | 0.00 | high | 47 | single distance per site | natural forest | Arthropods | passive | reported | 101 |
| Lucas-Garcia and Rosas-Guerrero, unpub | -0.53 | 0.18 | 0.00 | high | 18 | single distance per site | natural forest | Insecta | active | reported | 1107 |
| Obregon et al. | -0.12 | 0.15 | 0.42 | high | 10 | single distance per site | natural forest | Bees | active | estimated | 90 |
| Riojas-Lopez et al. 2019 | -0.48 | 0.19 | 0.01 | low | 8 | single distance per site | other | Bees | passive | reported | 870 |
| Schrader et al. 2018 | -0.28 | 0.15 | 0.06 | low | 10 | paired sites | other | Bees | active | estimated | 90 |
| Severiano-Galeana et al. 2024 | -0.27 | 0.28 | 0.32 | high | 24 | single distance per site | natural forest | Insecta | active | reported | 2000 |
| Tangtorwongsakul et al. 2017 | 0.18 | 0.20 | 0.37 | high | 24 | single distance per site | other | Bees | mixed | estimated | 5420 |
| Toledo-Hernandez et al. 2021 | 0.31 | 0.12 | 0.01 | low | 18 | single distance per site | other | Diptera | passive | reported | 3169 |
| Vogel et al. 2021 | 0.26 | 0.48 | 0.59 | low | 9 | single distance per site | other | Bees | active | estimated | 250 |
| Vogel et al. 2023 | -0.26 | 0.21 | 0.21 | low | 24 | single distance per site | other | Insecta | active | estimated | 200 |
| Wayo et al. 2020 | 0.44 | 0.17 | 0.01 | low | 30 | single distance per site | natural forest | Stingless Bees | active | reported | 9937 |

## Pollinator richness

**Figure S4.** Data and model fits for the relationship between pollinator richness and linear distance to the nearest natural habitat of each study using either GLMs or GLMMs with negative binomial error distribution (N= 30 studies; modelling approach indicated in the title of each plot).


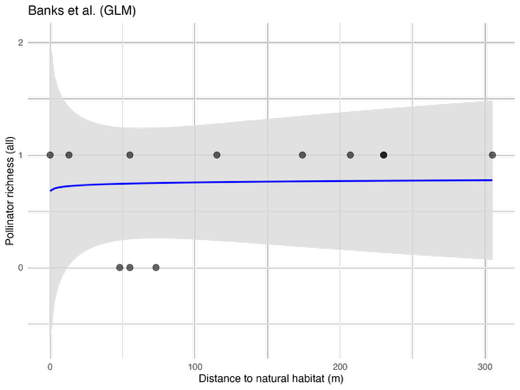

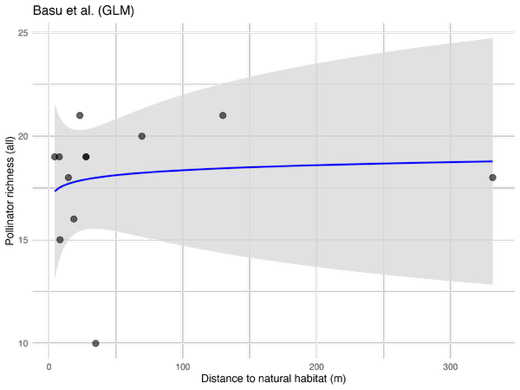

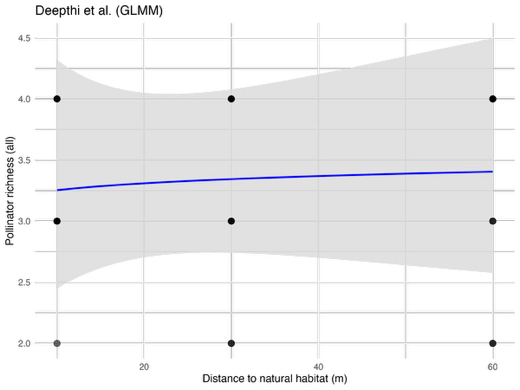

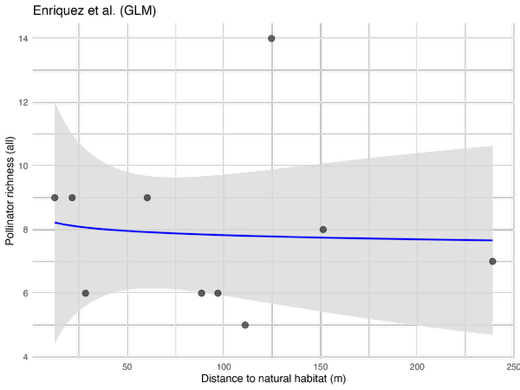

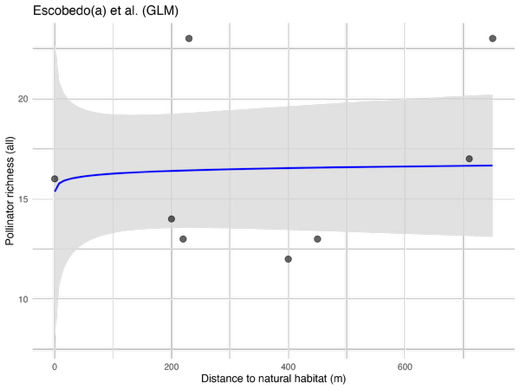

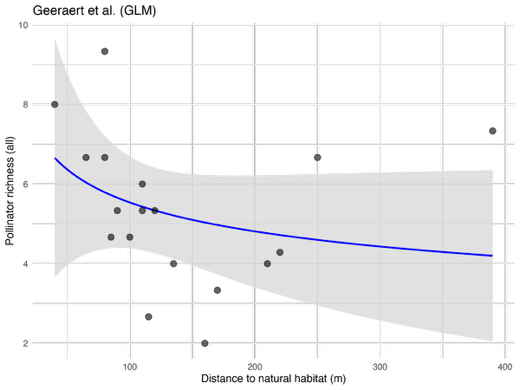

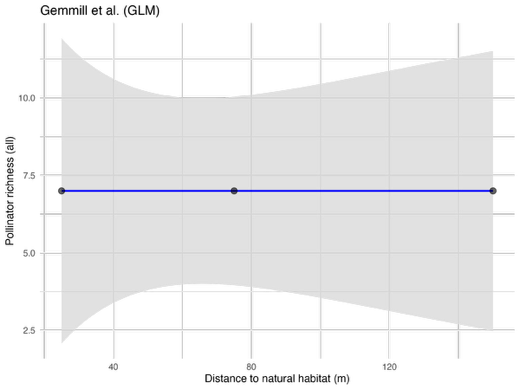

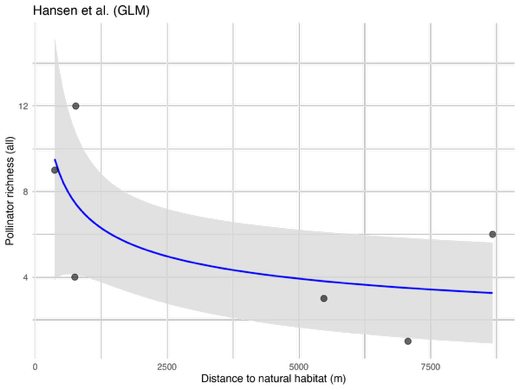

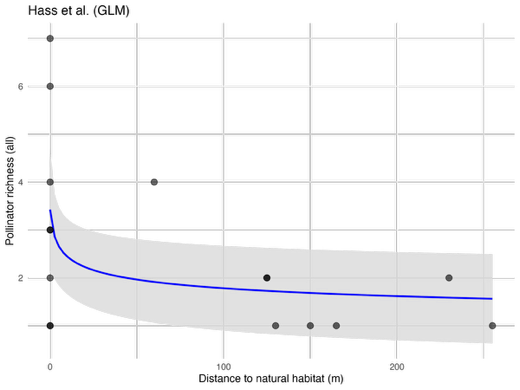

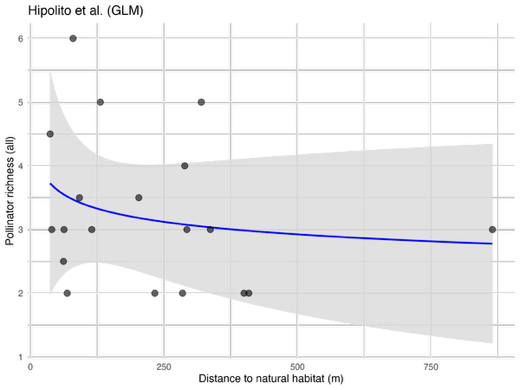

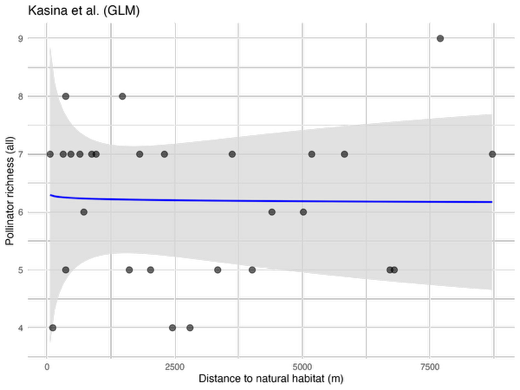

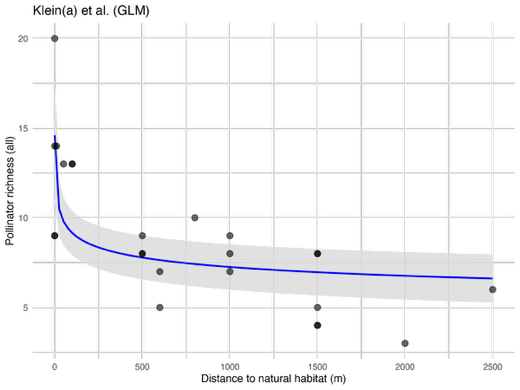

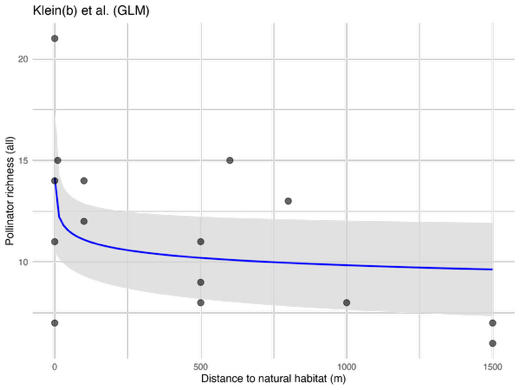

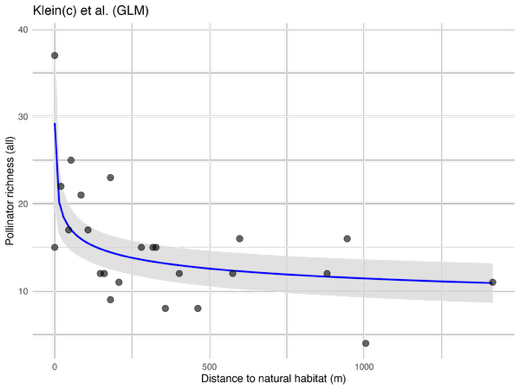

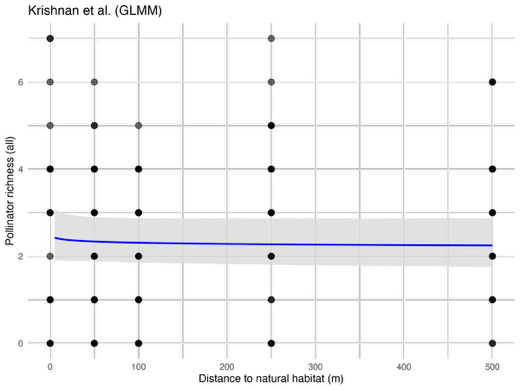

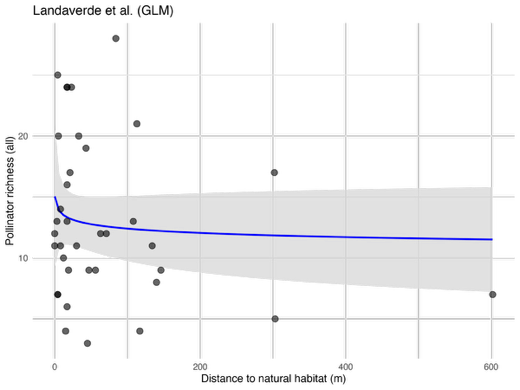

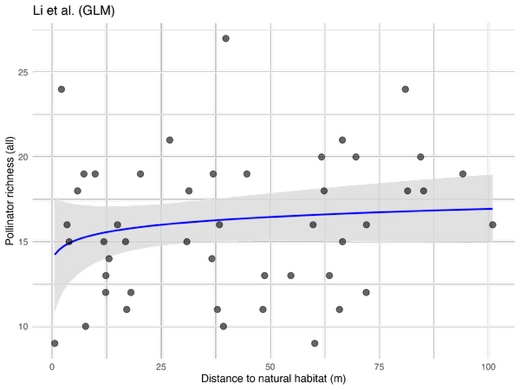

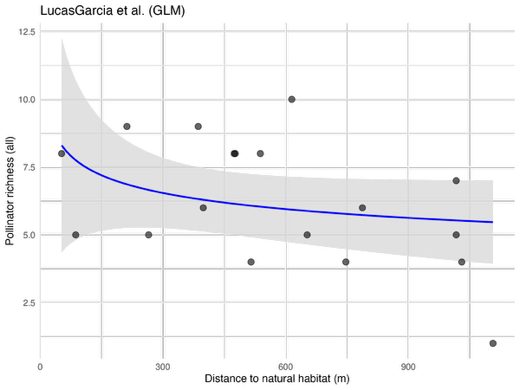

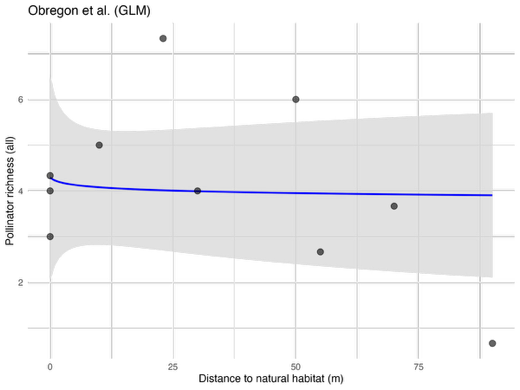

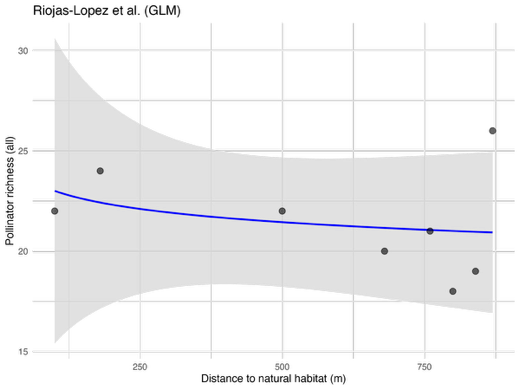

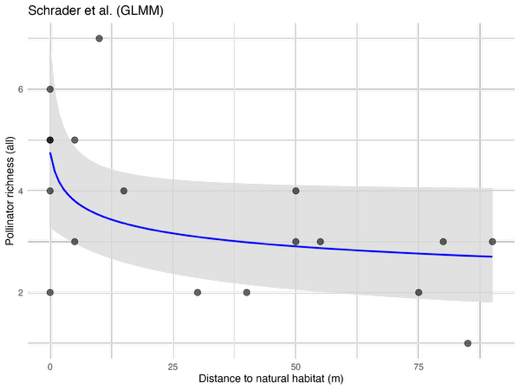

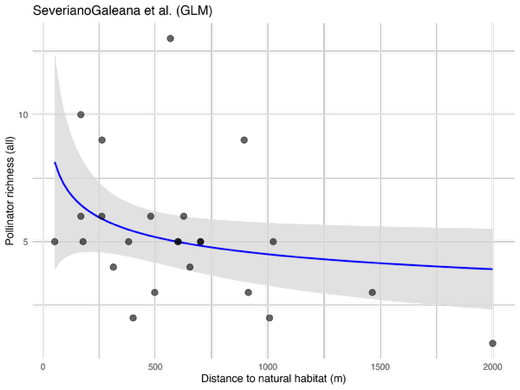

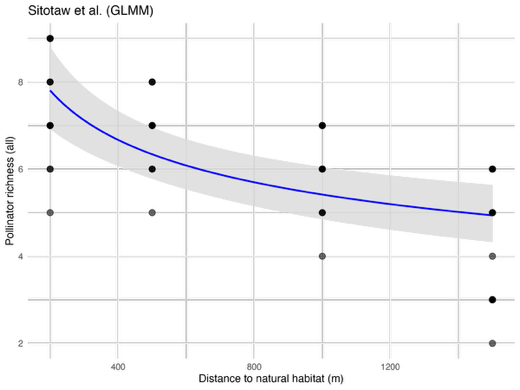

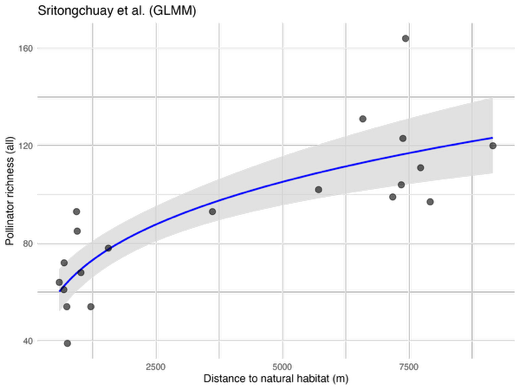

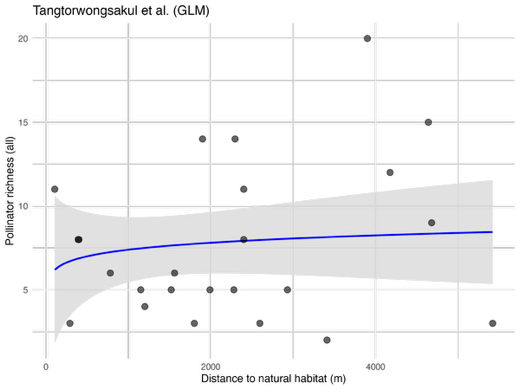

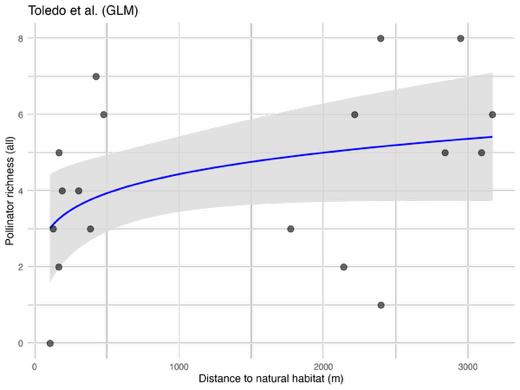

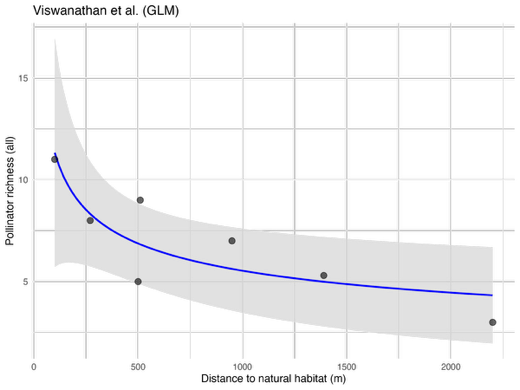

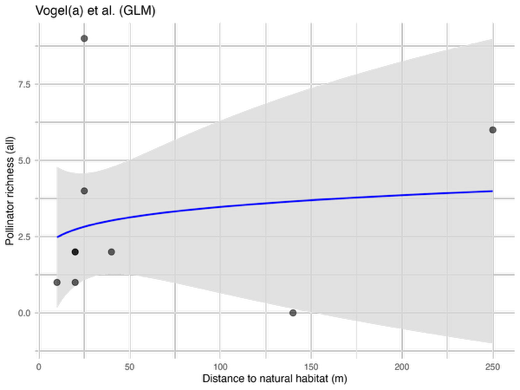

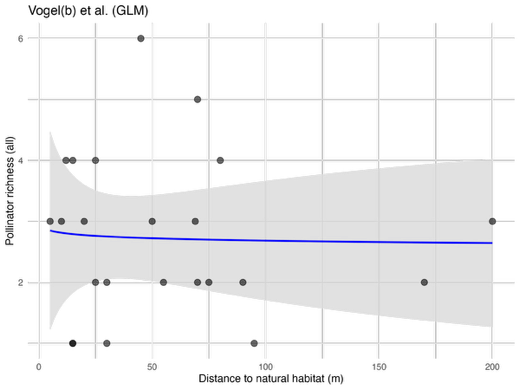

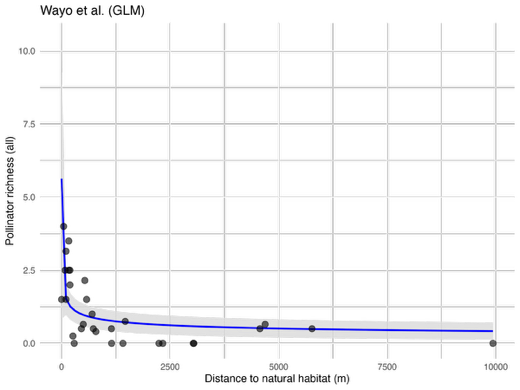


**Table S12.** Overview of effect sizes and moderator variables for the pollinator richness meta-analysis (N= 30 studies)

| **Authors** | **Slope** | **StdError** | **PValue** | **Agr Intensity** | **Sites** | **Design** | **Habitat** | **Pollinator** | **Method** | **Distance Measure** | **Max Distance** |
| --- | --- | --- | --- | --- | --- | --- | --- | --- | --- | --- | --- |
| Banks et al. 2013 | 0.02 | 0.22 | 0.92 | high | 12 | single distance per site | natural forest | Hymenoptera | passive | estimated | 305 |
| Basu et al. 2016 | 0.02 | 0.06 | 0.75 | both | 12 | single distance per site | other | Bees | passive | reported | 331 |
| Deepthi et al 2019 | 0.03 | 0.12 | 0.83 | high | 10 | nested design | natural forest | Bees | active | reported | 60 |
| Enriquez et al. 2015 | -0.02 | 0.13 | 0.85 | low | 10 | single distance per site | other | Bees | active | reported | 239 |
| Escobedo-Kenefic et al. 2022 | 0.01 | 0.04 | 0.79 | high | 8 | single distance per site | other | Insecta | active | estimated | 750 |
| Geeraert et al. 2020 | -0.20 | 0.20 | 0.30 | low | 15 | single distance per site | other | Bees | passive | estimated | 390 |
| Gemmill-Herren and Ochieng' 2008 | 0.00 | 0.30 | 1.00 | high | 25 | single distance per site | natural forest | Bees | active | reported | 150 |
| Hansen et al. 2020 | -0.34 | 0.17 | 0.04 | high | 6 | single distance per site | natural forest | Insecta | active | reported | 8676 |
| Hass et al. 2018 | -0.14 | 0.07 | 0.03 | high | 16 | single distance per site | other | Bees | active | reported | 255 |
| Hipolito et al. 2019 | -0.09 | 0.15 | 0.52 | both | 19 | single distance per site | other | Insecta | active | reported | 865 |
| Kasina et al. 2009 | 0.00 | 0.06 | 0.95 | low | 28 | single distance per site | natural forest | Bees | active | reported | 8721 |
| Klein et al. 2003 a) | -0.10 | 0.02 | 0.00 | low | 24 | single distance per site | natural forest | Bees | active | reported | 2500 |
| Klein et al. 2003 b) | -0.05 | 0.03 | 0.05 | low | 15 | single distance per site | natural forest | Bees | active | reported | 1500 |
| Klein 2009 | -0.14 | 0.03 | 0.00 | low | 24 | single distance per site | natural forest | Bees | active | reported | 1415 |
| Krishnan et al. 2012 | -0.02 | 0.03 | 0.51 | NA | 35 | nested design | natural forest | Bees | active | reported | 500 |
| Landaverde-Gonzales et al. 2017 | -0.04 | 0.06 | 0.45 | high | 37 | single distance per site | other | Bees | mixed | estimated | 602 |
| Li et al. 2022 | 0.04 | 0.04 | 0.28 | high | 47 | single distance per site | natural forest | Arthropods | passive | reported | 101 |
| Lucas-Garcia and Rosas-Guerrero, unpub | -0.14 | 0.11 | 0.21 | high | 18 | single distance per site | natural forest | Insecta | active | reported | 1107 |
| Obregon et al. 2021 | -0.02 | 0.09 | 0.81 | high | 10 | single distance per site | natural forest | Bees | active | estimated | 90 |
| Riojas-Lopez et al. 2019 | -0.04 | 0.10 | 0.66 | low | 8 | single distance per site | other | Bees | passive | reported | 870 |
| Schrader et al. 2018 | -0.13 | 0.07 | 0.07 | low | 10 | paired sites | other | Bees | active | estimated | 90 |
| Severiano-Galeana et al. 2024 | -0.20 | 0.12 | 0.09 | high | 24 | single distance per site | natural forest | Insecta | active | reported | 2000 |
| Sitotaw et al. 2022 | -0.23 | 0.04 | 0.00 | low | 72 | nested design | natural forest | Insecta | active | reported | 1500 |
| Sritongchuay et al. 2019 | 0.26 | 0.04 | 0.00 | low | 20 | paired sites | natural forest | Insecta | active | estimated | 9160 |
| Tangtorwongsakul et al. 2017 | 0.08 | 0.12 | 0.53 | high | 24 | single distance per site | other | Insecta | mixed | estimated | 5420 |
| Toledo-Hernandez et al. 2021 | 0.17 | 0.09 | 0.07 | low | 18 | single distance per site | other | Diptera | passive | reported | 3169 |
| Viswanathan et al. 2020 | -0.31 | 0.14 | 0.03 | both | 7 | single distance per site | natural forest | Insecta | active | reported | 2200 |
| Vogel et al. 2021 | 0.15 | 0.30 | 0.61 | low | 9 | single distance per site | other | Bees | active | estimated | 250 |
| Vogel et al. 2023 | -0.02 | 0.14 | 0.88 | low | 24 | single distance per site | other | Insecta | active | estimated | 200 |
| Wayo et al. 2020 | -0.28 | 0.08 | 0.00 | low | 30 | single distance per site | natural forest | Stingless Bees | active | reported | 9937 |

**Table S13.** Overview of the model outputs for the pollinator richness model, two moderator models (habitat type and agricultural intensity), and the four sensitivity models (pollinator sampling method, distance measure, maximum distance scales, and taxonomic resolution of species identification). Sample size (N) for each model or category is provided. Model outputs include the estimated effect sizes, standard errors (SE), z-values, p-values and 95% confidence intervals. Significance codes: * p < 0.05; ** p < 0.01.

| **model** | **N** | **category** | **estimate** | **se** | **zval** | **pval** | **ci.lb** | **ci.ub** |
| --- | --- | --- | --- | --- | --- | --- | --- | --- |
| **Main model:**  rma(yi = Slope, vi = Variance, data = richness_es) | 30 |  | -0.0537 | 0.0258 | -2.0785 | 0.0377 | -0.1044 | -0.0031* |
| **Moderator model 1**  rma(Slope, Variance, mods = ~ 0 + Habitat, data=richness_es) | 17 | natural forest | -0.0724 | 0.0336 | -2.1575 | 0.031 | -0.1382 | -0.0066* |
|  | 13 | other | -0.025 | 0.0419 | -0.5963 | 0.551 | -0.1071 | 0.0572 |
| **Moderator model 2**  rma(Slope, Variance, mods = ~ 0 + AgrIntensity, data=richness_es) | 3 | both | -0.0924 | 0.0939 | -0.9846 | 0.3248 | -0.2764 | 0.0916 |
|  | 12 | high | -0.0507 | 0.0457 | -1.1077 | 0.268 | -0.1403 | 0.039 |
|  | 14 | low | -0.0545 | 0.0387 | -1.4084 | 0.159 | -0.1303 | 0.0213 |
| **Sensitivity model 1**  rma(Slope, Variance, mods = ~ 0 + Method, data=richness_es) | 22 | active | -0.0778 | 0.0296 | -2.6302 | 0.0085 | -0.1358 | -0.0198** |
|  | 2 | mixed | 0.0006 | 0.0974 | 0.006 | 0.9952 | -0.1903 | 0.1915 |
|  | 6 | passive | 0.0263 | 0.0604 | 0.435 | 0.6635 | -0.092 | 0.1445 |
| **Sensitivity model 2**  rma(Slope, Variance, mods = ~ 0 + DistanceMeasure, data=richness_es) | 10 | estimated | 0.0223 | 0.0451 | 0.495 | 0.6206 | -0.066 | 0.1107 |
|  | 20 | reported | -0.0839 | 0.0289 | -2.9032 | 0.0037 | -0.1405 | -0.0273** |
| **Sensitivity model 3**  rma(Slope, Variance, mods = ~ 0 + DistanceCategory, data=richness_es) | 13 | small (<750m) | -0.0318 | 0.0377 | -0.8435 | 0.3989 | -0.1057 | 0.0421 |
|  | 10 | medium  (750m-3000m) | -0.1154 | 0.039 | -2.9627 | 0.003 | -0.1918 | -0.0391** |
|  | 6 | Large (>3000m) | 0.0231 | 0.0537 | 0.4301 | 0.6671 | -0.0821 | 0.1283 |
| **Sensitivity model 4**  rma(Slope, Variance, mods = ~ 0 + TaxonomicResolution, data=richness_es) | 22 | morphospecies | -0.0633 | 0.031 | -2.0403 | 0.0413 | -0.1241 | -0.0025* |
|  | 8 | other | -0.03 | 0.0496 | -0.6059 | 0.5446 | -0.1272 | 0.0671 |

**Table S14.** Results of the heterogeneity analysis for the main model, wild richness model, two moderator models (habitat type and agricultural intensity), and the four sensitivity models (pollinator sampling method, distance measure, maximum distance scales, and taxonomic resolution of species identification). Heterogeneity measures: tau^2^ = estimated amount of residual heterogeneity; tau = square root of estimated tau^2^ value; I^2^ = residual heterogeneity / unaccounted variability; H^2^ = unaccounted variability / sampling variability; Q test = Test for Heterogeneity; For moderator analyses: QE = Test for residual Heterogeneity, QM = Test of Moderators

| **model** | **tau^^2^** | **tau** | **I^2^** | **H^2^** | **Q statistic** | **p-val** |
| --- | --- | --- | --- | --- | --- | --- |
| **Main model:** rma(yi = Slope, vi = Variance, data = richness_es) | 0.0118 (SE = 0.0049) | 0.1088 | 79.49% | 4.88 | Q(df = 29) = 131.3537 | < .0001 |
| **Moderator model 1**  rma(Slope, Variance, mods = ~ 0 + Habitat, data=richness_es) | 0.0123 (SE = 0.0051) | 0.1109 | 79.90% | 4.97 | QE(df = 28) = 130.1064  QM(df = 2) = 5.0106 | < .0001  0.0817 |
| **Moderator model 2**  rma(Slope, Variance, mods = ~ 0 + AgrIntensity, data=richness_es) | 0.0140 (SE = 0.0060) | 0.1183 | 79.63% | 4.91 | QE(df = 26) = 126.4499  QM(df = 3) = 4.1799 | < .0001  0.2427 |
| **Sensitivity model 1**  rma(Slope, Variance, mods = ~ 0 + Method, data=richness_es) | 0.0116 (SE = 0.0050) | 0.1075 | 78.74% | 4.70 | QE(df = 27) = 121.2037  QM(df = 3) = 7.1072 | < .0001  0.0686 |
| **Sensitivity model 2**  rma(Slope, Variance, mods = ~ 0 + DistanceMeasure, data=richness_es) | 0.0100 (SE = 0.0044) | 0.0998 | 76.06% | 4.18 | QE(df = 28) = 99.7528  QM(df = 2) = 8.6735 | < .0001  0.0131 |
| **Sensitivity model 3**  rma(Slope, Variance, mods = ~ 0 + DistanceCategory, data=richness_es) | 0.0098 (SE = 0.0044) | 0.0989 | 74.39% | 3.91 | QE(df = 27) = 85.6750  QM(df = 3) = 9.6741 | < .0001  0.0215 |
| **Sensitivity model 4**  rma(Slope, Variance, mods = ~ 0 + TaxonomicResolution, data=richness_es) | 0.0124 (SE = 0.0052) | 0.1115 | 79.75% | 4.94 | QE(df = 28) = 131.0284  QM(df = 2) = 4.5299 | < .0001  0.1038 |

## Fruit set

**Figure S5.** Data and model fits for the relationship between fruit set and distance to the nearest natural habitat of each study using beta regression models (GLMs for single-distance-per-site datasets and GLMMs for datasets with nested and paired study designs; N = 17 studies).


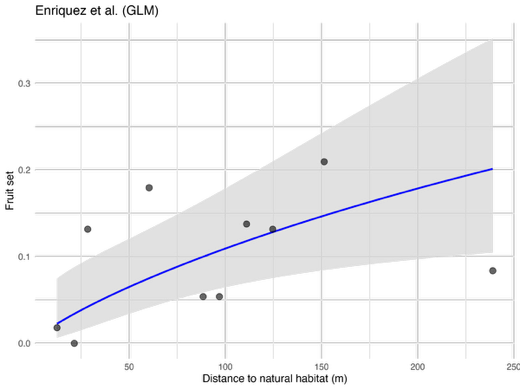

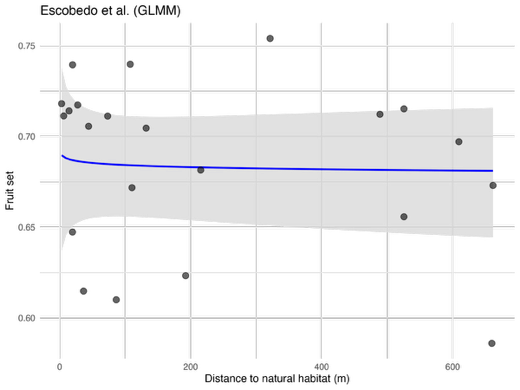

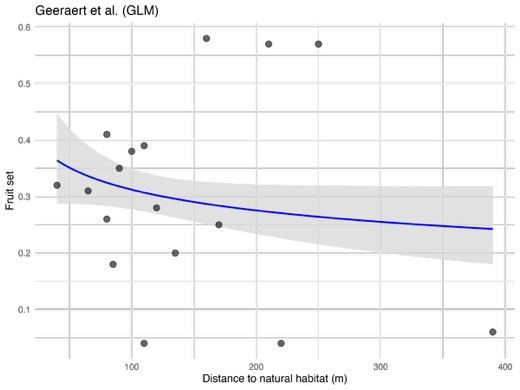

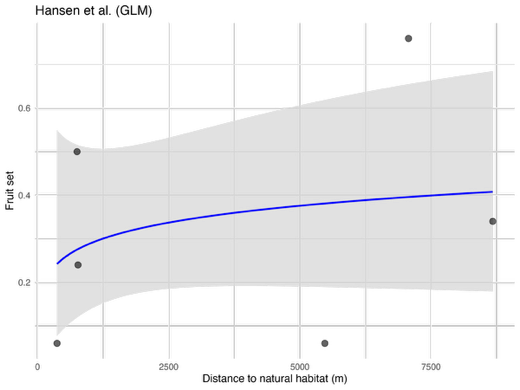

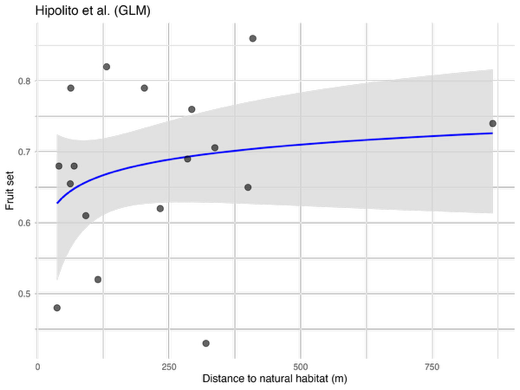

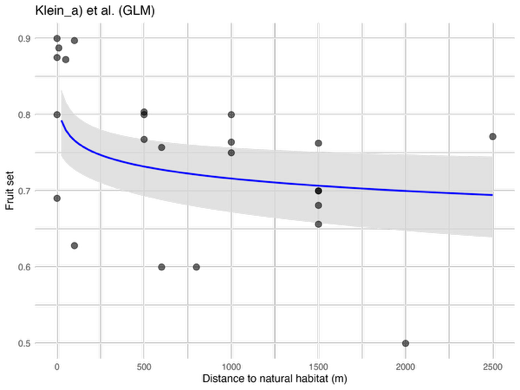

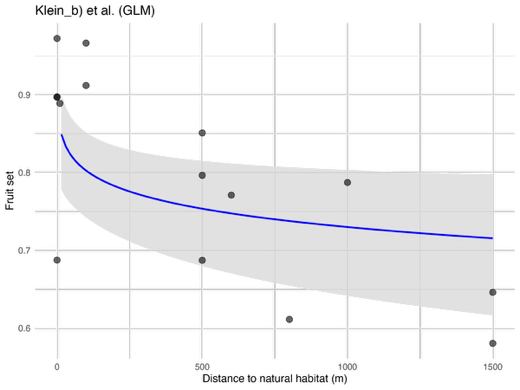

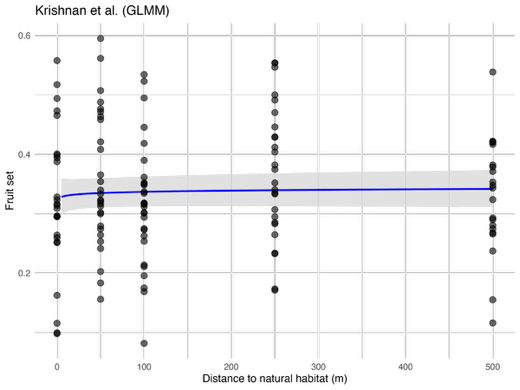

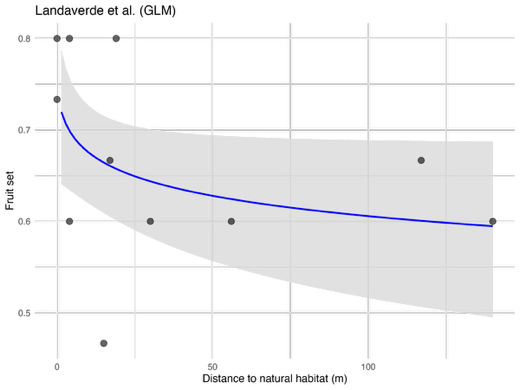

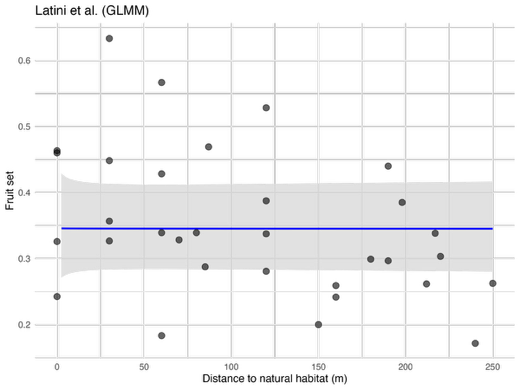

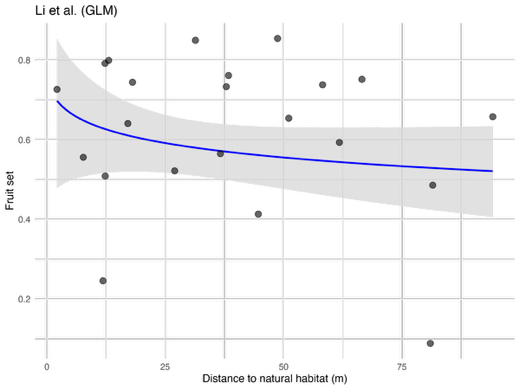

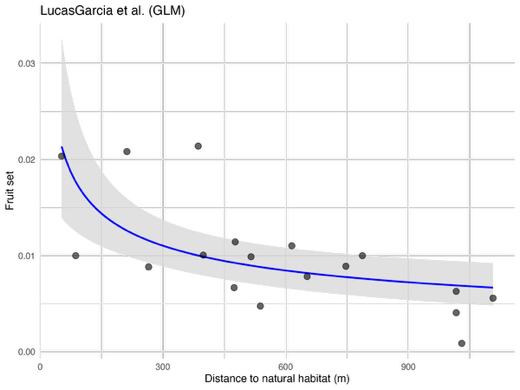

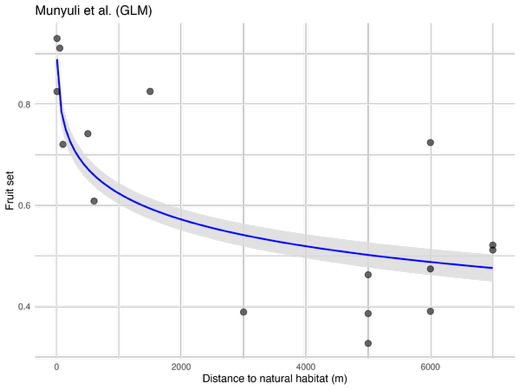

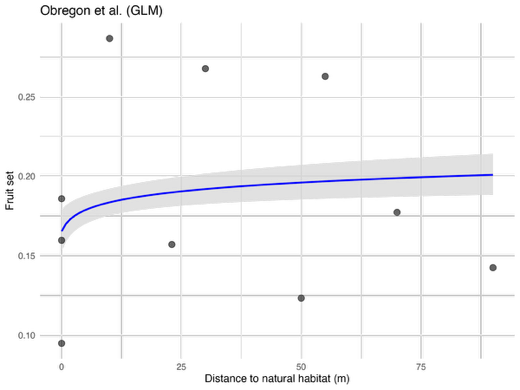

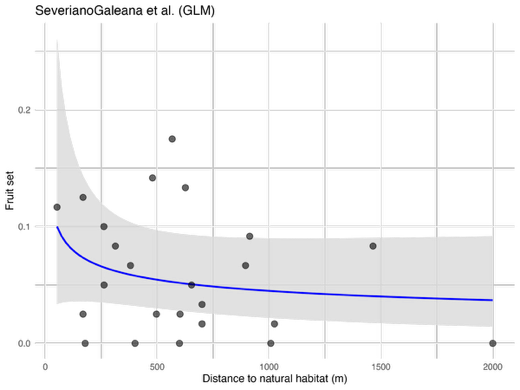

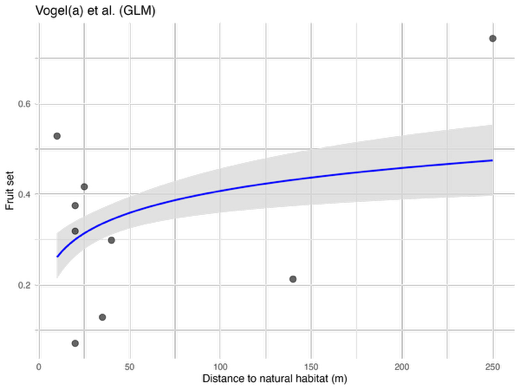

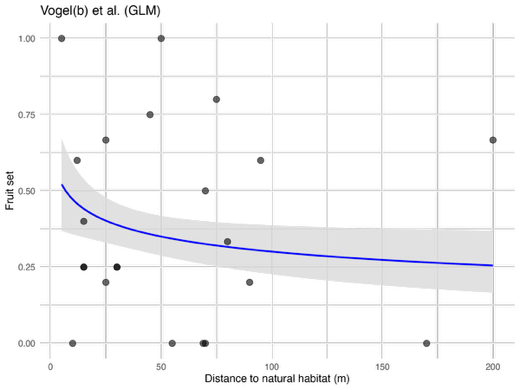


**Table S15.** Overview of effect sizes and moderator variables for the fruit set meta-analysis (N = 17 studies).

| **Authors** | **Slope** | **StdError** | **PValue** | **Crop** | **Poll Dependency** | **Agr Intensity** | **Sites** | **Design** | **Habitat** | **Distance Measure** | **Max Distance** |
| --- | --- | --- | --- | --- | --- | --- | --- | --- | --- | --- | --- |
| Enriquez et al. 2015 | 0.83 | 0.29 | 0.00 | squash | Essential (90-100%) | low | 10 | single distance per site | other | reported | 239 |
| Escobedo-Kenefic et al. 2024 | -0.01 | 0.03 | 0.80 | Brassica rapa (rape, rape seed) | Modest (10-40%) | both | 6 | nested design | natural forest | reported | 661 |
| Geeraert et al. 2020 | -0.26 | 0.15 | 0.08 | coffee | Modest (10-40%) | low | 18 | single distance per site | other | estimated | 390 |
| Hansen et al. 2020 | 0.24 | 0.31 | 0.43 | guava | Little (0-10%) | high | 6 | single distance per site | natural forest | reported | 8676 |
| Hipolito et al. 2019 | 0.15 | 0.14 | 0.29 | coffee arabica | Modest (10-40%) | both | 19 | single distance per site | other | reported | 865 |
| Klein et al. 2003 a) | -0.11 | 0.04 | 0.00 | coffee arabica | Modest (10-40%) | low | 24 | single distance per site | natural forest | reported | 2500 |
| Klein et al. 2003 b) | -0.18 | 0.07 | 0.01 | coffee canephora | Great (40-90%) | low | 15 | single distance per site | natural forest | reported | 1500 |
| Krishnan et al. 2012 | 0.01 | 0.02 | 0.48 | coffee | Great (40-90%) | NA | 35 | nested design | natural forest | reported | 500 |
| Landaverde-Gonzales et al. 2017 | -0.14 | 0.08 | 0.07 | chilli | Great (40-90%) | high | 11 | single distance per site | other | estimated | 140 |
| Latini et al 2020 | 0.00 | 0.04 | 0.99 | coffee | Modest (10-40%) | both | 8 | nested design | natural forest | reported | 250 |
| Li et al. 2022 | -0.22 | 0.19 | 0.24 | oil palm | Little (0-10%) | high | 22 | single distance per site | natural forest | reported | 94 |
| Lucas-Garcia and Rosas-Guerrero, unpub | -0.39 | 0.10 | 0.00 | mango | Great (40-90%) | high | 18 | single distance per site | natural forest | reported | 1107 |
| Munyuli 2012 | -0.31 | 0.02 | 0.00 | coffee | Great (40-90%) | low | 16 | single distance per site | other | reported | 7000 |
| Obregon et al. 2021 | 0.05 | 0.02 | 0.00 | lulo' or 'naranjilla' | Essential (90-100%) | high | 10 | single distance per site | natural forest | estimated | 90 |
| Severiano-Galeana et al. 2024 | -0.29 | 0.24 | 0.23 | mango | Great (40-90%) | high | 24 | single distance per site | natural forest | reported | 2000 |
| Vogel et al. (a) 2021 | 0.30 | 0.08 | 0.00 | pigeonpea | Modest (10-40%) | low | 9 | single distance per site | other | estimated | 250 |
| Vogel et al. (b) 2023 | -0.33 | 0.15 | 0.03 | pumpkin | Essential (90-100%) | low | 24 | single distance per site | other | estimated | 200 |

**Table S16.** Overview of the model outputs for the fruit set model, three moderator models (habitat type, agricultural intensity, and crop pollinator-dependency), and the two sensitivity models (distance measure, and maximum distance scales,). Sample size (N) for each model or category is provided. Model outputs include the estimated effect sizes, standard errors (SE), z-values, p-values and 95% confidence intervals.

| **model** | **N** | **category** | **estimate** | **se** | **zval** | **pval** | **ci.lb** | **ci.ub** |
| --- | --- | --- | --- | --- | --- | --- | --- | --- |
| **Main model:**  rma(yi = Slope, vi = StdError^2, data = fruitset_es) | 17 |  | -0.068 | 0.053 | -0.171 | 0.035 | -0.446 | 0.311 |
| **Moderator model 1**  rma(Slope, Variance, mods = ~ 0 + AgrIntensity, data=fruitset_es) | 3 | both | 0.0354 | 0.1284 | 0.276 | 0.7825 | -0.2162 | 0.2871 |
|  | 6 | high | -0.1385 | 0.1048 | -1.3215 | 0.1863 | -0.3438 | 0.0669 |
|  | 7 | low | -0.0776 | 0.0899 | -0.8633 | 0.388 | -0.2538 | 0.0986 |
| **Moderator model 2**  rma(Slope, Variance, mods = ~ 0 + Habitat, data=fruitset_es) | 10 | natural forest | -0.087 | 0.0711 | -1.2224 | 0.2215 | -0.2264 | 0.0525 |
|  | 7 | other | -0.0359 | 0.0886 | -0.4055 | 0.6851 | -0.2096 | 0.1378 |
| **Moderator model 3**  rma(Slope, Variance, mods = ~ 0 + PollDependency, data=fruitset_es) | 3 | Essential (90-100%) | 0.0448 | 0.1287 | 0.3482 | 0.7277 | -0.2075 | 0.2971 |
|  | 6 | Great (40-90%) | -0.199 | 0.0802 | -2.4811 | 0.0131 | -0.3563 | -0.0418* |
|  | 2 | Little (0-10%) | -0.061 | 0.2072 | -0.2945 | 0.7684 | -0.4671 | 0.3451 |
|  | 6 | Modest (10-40%) | 0.0145 | 0.0789 | 0.1834 | 0.8545 | -0.1402 | 0.1691 |
| **Sensitivity model 1**  rma(Slope, Variance, mods = ~ 0 + DistanceMeasure, data=fruitset_es) | 5 | estimated | -0.046 | 0.097 | -0.4739 | 0.6356 | -0.2361 | 0.1442 |
|  | 12 | reported | -0.077 | 0.0657 | -1.1732 | 0.2407 | -0.2057 | 0.0517 |
| **Sensitivity model 2**  rma(Slope, Variance, mods = ~ 0 + DistanceCategory, data=fruitset_es) | 10 | Small (<750m) | -0.0045 | 0.0621 | -0.0729 | 0.9419 | -0.1262 | 0.1172 |
|  | 5 | Medium (750m-3000m) | -0.1588 | 0.0909 | -1.7471 | 0.0806 | -0.3368 | 0.0193 . |
|  | 2 | Large (>3000m) | -0.203 | 0.1532 | -1.3255 | 0.185 | -0.5033 | 0.0972 |

**Table S17.** Results of the heterogeneity analysis for the fruit set model, three moderator models (habitat type, agricultural intensity, and crop pollinator-dependency), and the two sensitivity models (distance measure, maximum distance scales). Heterogeneity measures: tau^2^ = estimated amount of residual heterogeneity; tau = square root of estimated tau^2^ value; I^2^ = residual heterogeneity / unaccounted variability; H^2^ = unaccounted variability / sampling variability; Q test = Test for Heterogeneity; For moderator analyses: QE = Test for residual Heterogeneity, QM = Test of Moderators

| **model** | **tau^2^** | **tau** | **I^2^** | **H^2^** | **Q statistic** | **p-val** |
| --- | --- | --- | --- | --- | --- | --- |
| **Main model:** rma(yi = Slope, vi = StdError^2, data = fruitset_es) | 0.0345 (SE = 0.0159) | 0.1858 | 95.48% | 22.12 | Q(df = 16) = 255.3045 | < .0001 |
| **Moderator model 1**  rma(Slope, Variance, mods = ~ 0 + AgrIntensity, data=fruitset_es) | 0.0437 (SE = 0.0224) | 0.2091 | 91.55% | 11.84 | QE(df = 13) = 108.4437  QM(df = 3) = 2.5679 | < .0001  0.4631 |
| **Moderator model 2**  rma(Slope, Variance, mods = ~ 0 + Habitat, data=fruitset_es) | 0.0394 (SE = 0.0185) | 0.1985 | 95.37% | 21.58 | QE(df = 15) = 122.8284  QM(df = 2) = 1.6588 | < .0001  0.4363 |
| **Moderator model 3**  rma(Slope, Variance, mods = ~ 0 + PollDependency, data=fruitset_es) | 0.0306 (SE = 0.0158) | 0.1749 | 92.30% | 12.99 | QE(df = 13) = 171.1500  QM(df = 4) = 6.3974 | < .0001  0.1714 |
| **Sensitivity model 1**  rma(Slope, Variance, mods = ~ 0 + DistanceMeasure, data=fruitset_es) | 0.0375 (SE = 0.0177) | 0.1937 | 94.33% | 17.63 | QE(df = 15) = 193.9618  QM(df = 2) = 1.6010 | < .0001  0.4491 |
| **Sensitivity model 2**  rma(Slope, Variance, mods = ~ 0 + DistanceCategory, data=fruitset_es) | 0.0285 (SE = 0.0148) | 0.1689 | 93.37% | 15.08 | QE(df = 14) = 55.3663  QM(df = 3) = 4.8145 | < .0001  0.1859 |

# Sensitivity analyses

Alongside conducting sensitivity analyses to test whether the presence of outliers or influential studies may affect the meta-analysis, we also conducted multiple sensitivity analyses to test for the potential effects of: 1) method of sampling pollinators; 2) method of measuring distance to nearest natural habitat; 3) scale of maximum distances considered in each study, and 4) taxonomic resolution of species identification (only for the pollinator richness analysis). We had initially also planned a sensitivity analysis of study-level risk of bias on the meta-analytic results, however, this was not conducted due to challenges with applying the risk of bias assessment tool and we only report the qualitative outcomes of the risk of bias assessment.

For method of sampling pollinators, we used moderator analysis to test whether effect sizes varied based on the sampling approach: ‘active’ (such as timed observations of flower visitors either in plots or along transects, often by sweep netting), ‘passive’ (such as pan traps, sticky traps and glue traps) or ‘mixed’ (combining both approaches) sampling methods. This was because active and passive pollinator sampling methods provide distinct assessments of the insect community by capturing species with different traits and at different abundances (O'Connor et al. 2019, Thompson et al. 2021).

We also ran a separate moderator analysis to test whether effect sizes varied between studies that reported distance directly (‘reported’) and those where distances were derived from satellite imagery (‘estimated’) because they were not reported in the original publication. The main concern with ‘estimated’ distances is the potential for inaccuracies in identifying natural habitat without field validation or familiarity with the study area, as well as discrepancies between the timing of satellite imagery and field data collection and potential misalignment in georeferencing, all of which could introduce inaccuracies in the distance measurements and affect the robustness of the results. Moreover, we conducted a moderator analysis using a categorical variable based on the maximum distance scale investigated in each study. This allowed us to explore whether the effect of distance on pollination variables varied across different spatial scales. Based on known pollinator foraging ranges, which vary from several hundred meters for solitary bees to up to 3 km for honeybees (Steffan-Dewenter et al. 2002), we categorised the maximum distances as follows: ‘small’ (<750m), ‘medium’ (750–3000m), and ‘large’ (>3000m)

## Pollinator abundance


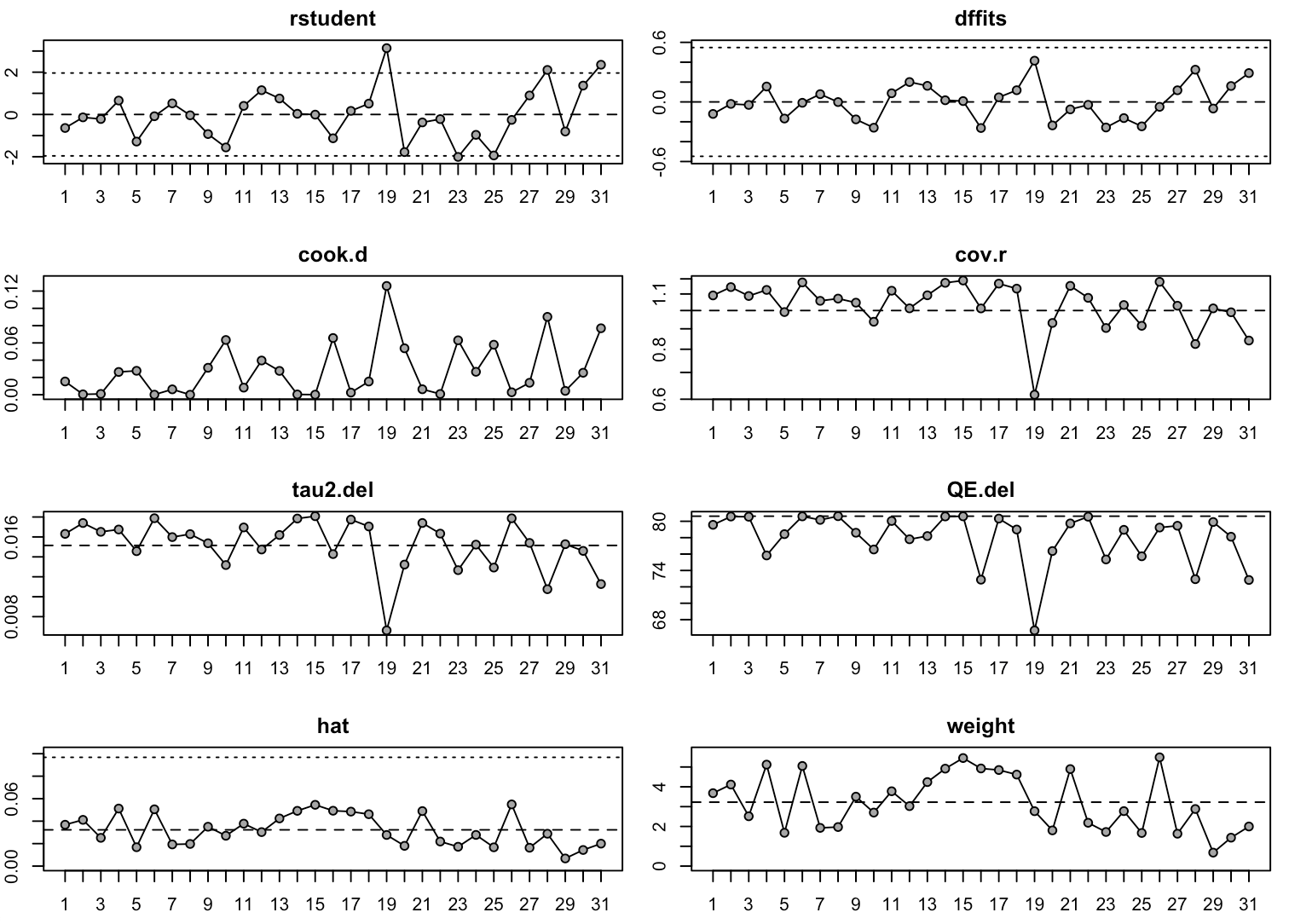

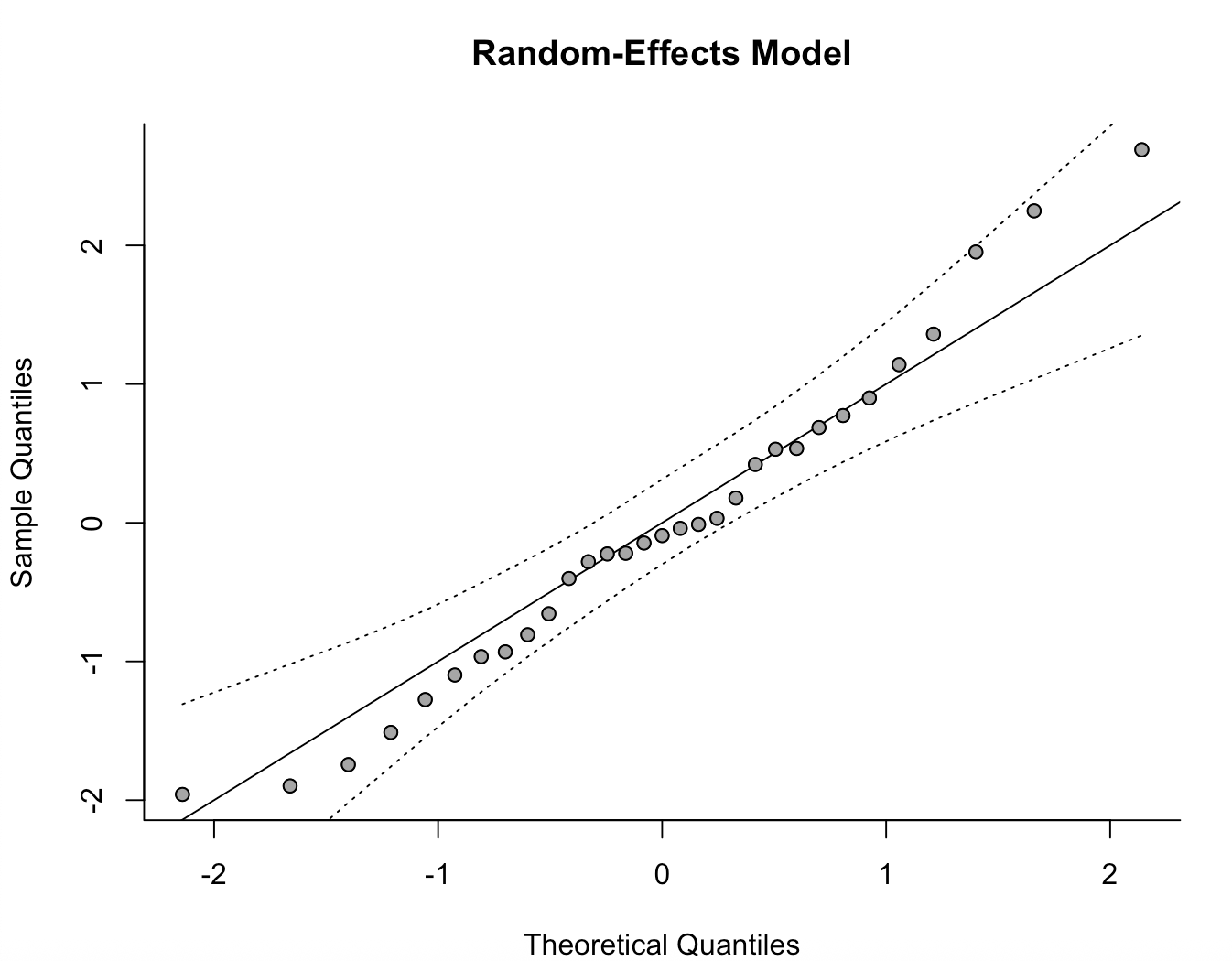


**Figure S6.** **Left:** Influential study analysis for the pollinator abundance meta-analysis (N = 31 studies) using the influence() function from the *metafor* package in R. Each data point represents one study included in the meta-analysis, and red dots represent studies flagged as influential based on key influence diagnostics, such as studentised residuals, Cook’s distance, and covariance ratios metrics (Viechtbauer and Cheung 2010). **Right:** Normal Q-Q plot of residuals from the random-effects meta-analysis model. The plot compares the standardised residuals to a theoretical normal distribution. It indicates that the residuals conform to normality, suggesting that the model assumptions are appropriately met.

**Table S18.** Leave-one-out sensitivity analysis for the meta-analysis on pollinator abundance (N = 31 studies). The table reports the effect size estimate (estimate), standard error (se), z-value (zval), and 95% confidence interval (ci.lb, ci.ub) after excluding each study. Heterogeneity measures include Cochran’s Q statistic (Q) and p-value (Qp), between-study variance (tau²), proportion of variation due to heterogeneity (I²), and total-to-sampling variability ratio (H²).

| **estimate** | **se** | **zval** | **pval** | **ci.lb** | **ci.ub** | **Q** | **Qp** | **tau2** | **I2** | **H2** |
| --- | --- | --- | --- | --- | --- | --- | --- | --- | --- | --- |
| -0.03 | 0.03 | -0.84 | 0.40 | -0.09 | 0.03 | 79.57 | 0.00 | 0.02 | 76.29 | 4.22 |
| -0.03 | 0.03 | -0.92 | 0.36 | -0.09 | 0.03 | 80.58 | 0.00 | 0.02 | 77.29 | 4.40 |
| -0.03 | 0.03 | -0.93 | 0.35 | -0.09 | 0.03 | 80.55 | 0.00 | 0.02 | 76.73 | 4.30 |
| -0.03 | 0.03 | -1.10 | 0.27 | -0.10 | 0.03 | 75.83 | 0.00 | 0.02 | 75.44 | 4.07 |
| -0.02 | 0.03 | -0.84 | 0.40 | -0.08 | 0.03 | 78.43 | 0.00 | 0.01 | 74.51 | 3.92 |
| -0.03 | 0.03 | -0.92 | 0.36 | -0.09 | 0.03 | 80.60 | 0.00 | 0.02 | 76.81 | 4.31 |
| -0.03 | 0.03 | -1.05 | 0.29 | -0.09 | 0.03 | 80.17 | 0.00 | 0.02 | 76.21 | 4.20 |
| -0.03 | 0.03 | -0.97 | 0.33 | -0.09 | 0.03 | 80.62 | 0.00 | 0.02 | 76.54 | 4.26 |
| -0.02 | 0.03 | -0.81 | 0.42 | -0.08 | 0.03 | 78.60 | 0.00 | 0.02 | 75.25 | 4.04 |
| -0.02 | 0.03 | -0.78 | 0.44 | -0.08 | 0.03 | 76.56 | 0.00 | 0.01 | 72.44 | 3.63 |
| -0.03 | 0.03 | -1.03 | 0.30 | -0.09 | 0.03 | 80.04 | 0.00 | 0.02 | 76.96 | 4.34 |
| -0.04 | 0.03 | -1.19 | 0.23 | -0.09 | 0.02 | 77.80 | 0.00 | 0.01 | 74.57 | 3.93 |
| -0.03 | 0.03 | -1.12 | 0.26 | -0.10 | 0.03 | 78.20 | 0.00 | 0.02 | 75.94 | 4.16 |
| -0.03 | 0.03 | -0.94 | 0.35 | -0.09 | 0.03 | 80.59 | 0.00 | 0.02 | 77.04 | 4.36 |
| -0.03 | 0.03 | -0.93 | 0.35 | -0.09 | 0.03 | 80.60 | 0.00 | 0.02 | 75.07 | 4.01 |
| -0.02 | 0.03 | -0.74 | 0.46 | -0.08 | 0.04 | 72.87 | 0.00 | 0.01 | 72.84 | 3.68 |
| -0.03 | 0.03 | -0.97 | 0.33 | -0.09 | 0.03 | 80.33 | 0.00 | 0.02 | 77.05 | 4.36 |
| -0.03 | 0.03 | -1.06 | 0.29 | -0.10 | 0.03 | 79.00 | 0.00 | 0.02 | 76.60 | 4.27 |
| -0.04 | 0.02 | -1.73 | 0.08 | -0.09 | 0.01 | 66.69 | 0.00 | 0.01 | 56.89 | 2.32 |
| -0.02 | 0.03 | -0.80 | 0.42 | -0.08 | 0.03 | 76.37 | 0.00 | 0.01 | 72.62 | 3.65 |
| -0.03 | 0.03 | -0.86 | 0.39 | -0.09 | 0.03 | 79.74 | 0.00 | 0.02 | 76.62 | 4.28 |
| -0.03 | 0.03 | -0.94 | 0.35 | -0.09 | 0.03 | 80.56 | 0.00 | 0.02 | 76.57 | 4.27 |
| -0.02 | 0.03 | -0.79 | 0.43 | -0.08 | 0.03 | 75.34 | 0.00 | 0.01 | 71.76 | 3.54 |
| -0.02 | 0.03 | -0.83 | 0.41 | -0.08 | 0.03 | 78.96 | 0.00 | 0.02 | 75.22 | 4.04 |
| -0.02 | 0.03 | -0.80 | 0.43 | -0.08 | 0.03 | 75.73 | 0.00 | 0.01 | 72.17 | 3.59 |
| -0.03 | 0.03 | -0.87 | 0.38 | -0.09 | 0.03 | 79.23 | 0.00 | 0.02 | 74.47 | 3.92 |
| -0.03 | 0.03 | -1.10 | 0.27 | -0.09 | 0.03 | 79.45 | 0.00 | 0.02 | 75.55 | 4.09 |
| -0.04 | 0.03 | -1.43 | 0.15 | -0.09 | 0.01 | 72.94 | 0.00 | 0.01 | 68.18 | 3.14 |
| -0.03 | 0.03 | -0.93 | 0.35 | -0.09 | 0.03 | 79.91 | 0.00 | 0.02 | 75.46 | 4.07 |
| -0.03 | 0.03 | -1.17 | 0.24 | -0.09 | 0.02 | 78.11 | 0.00 | 0.01 | 74.57 | 3.93 |
| -0.04 | 0.03 | -1.40 | 0.16 | -0.09 | 0.02 | 72.84 | 0.00 | 0.01 | 69.30 | 3.26 |

**Publication bias**

Egger’s regression test showed no evidence of small-study effects in the pollinator abundance meta-analysis (z = -0.50, p = 0.62, 95% CI: -0.12 to 0.11), suggesting no significant publication bias.


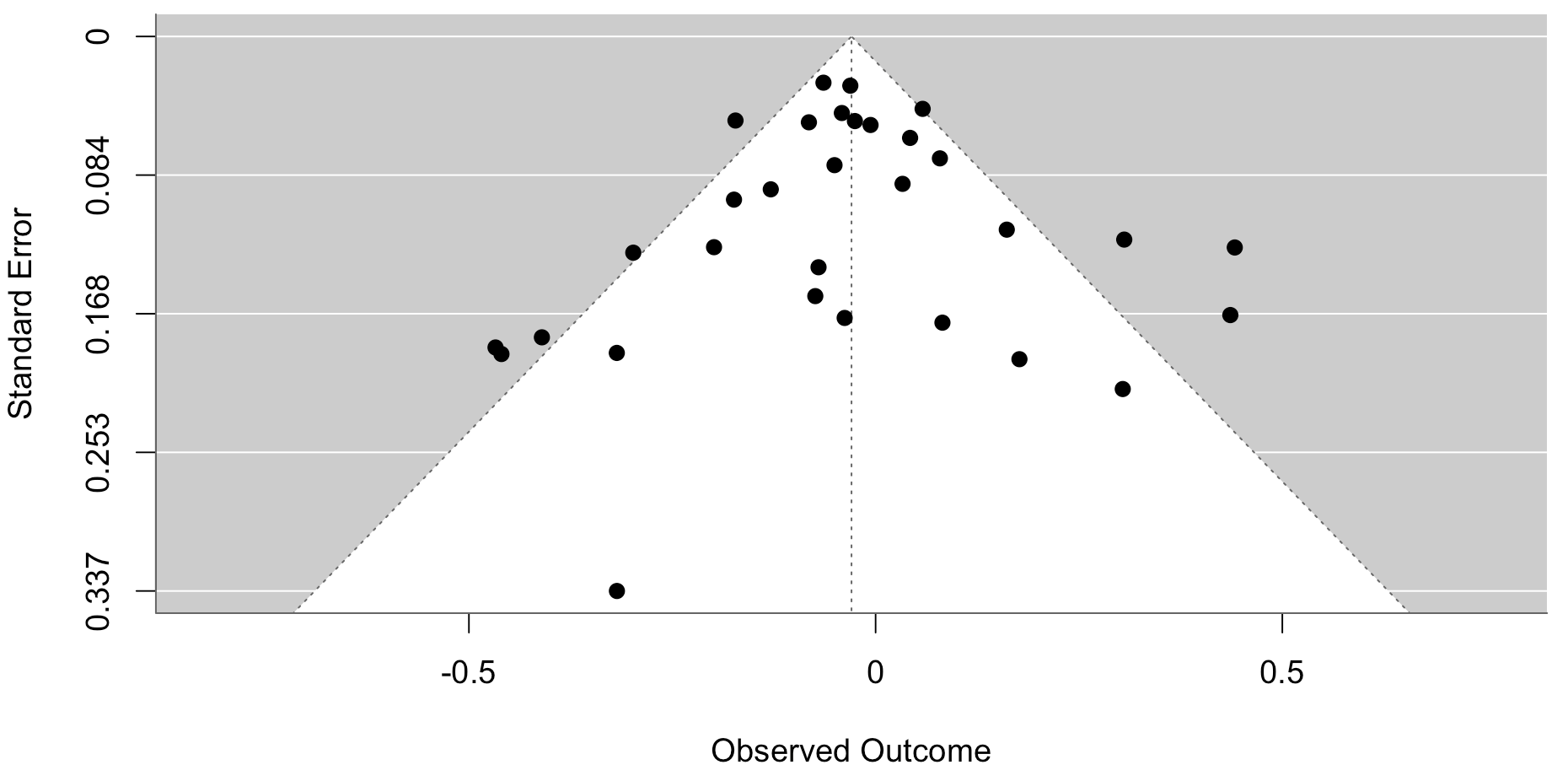


**Figure S7.** Funnel plot for the pollinator abundance meta-analysis. A symmetric distribution of effect sizes suggests no strong evidence of publication bias.

## Pollinator richness


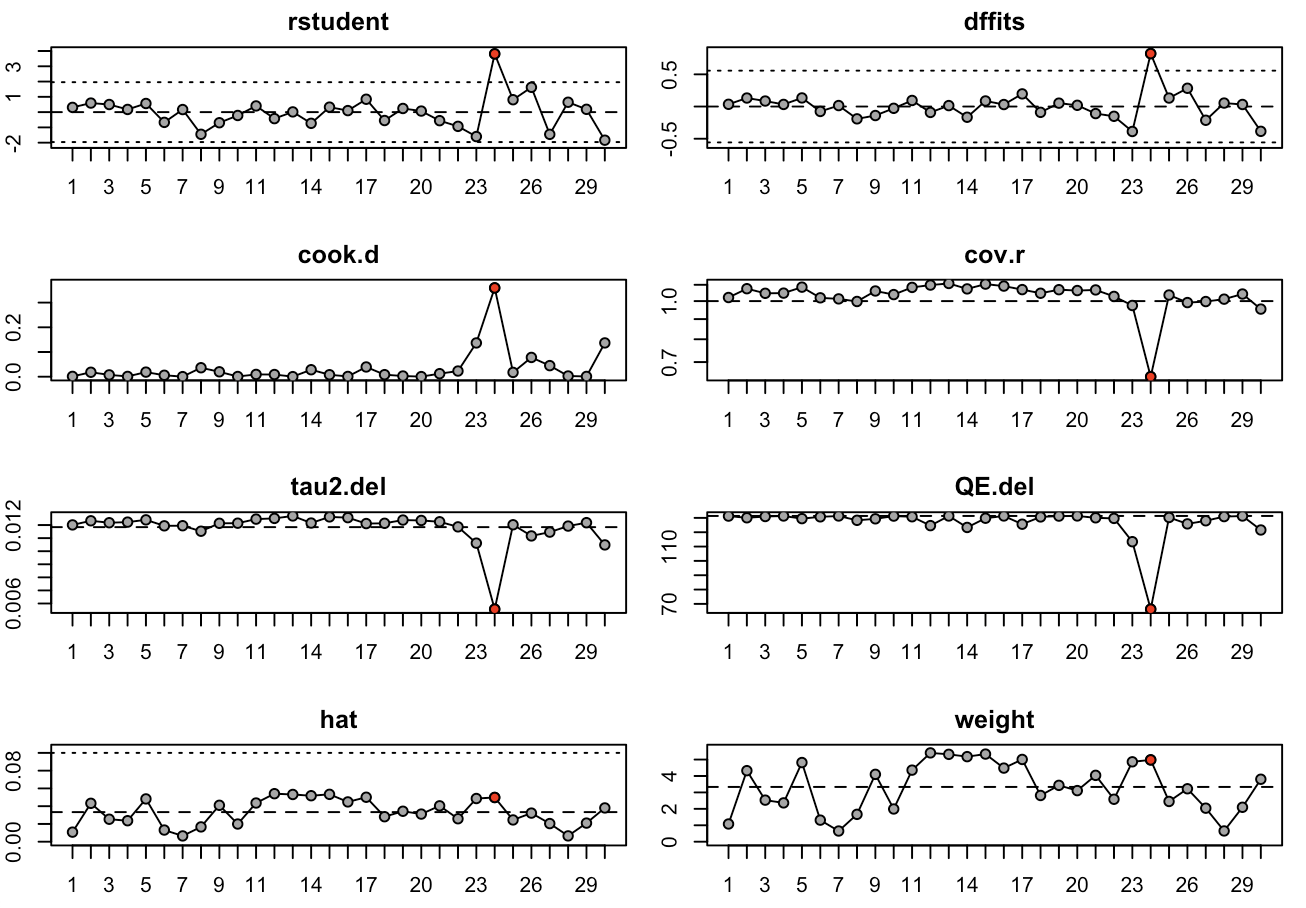

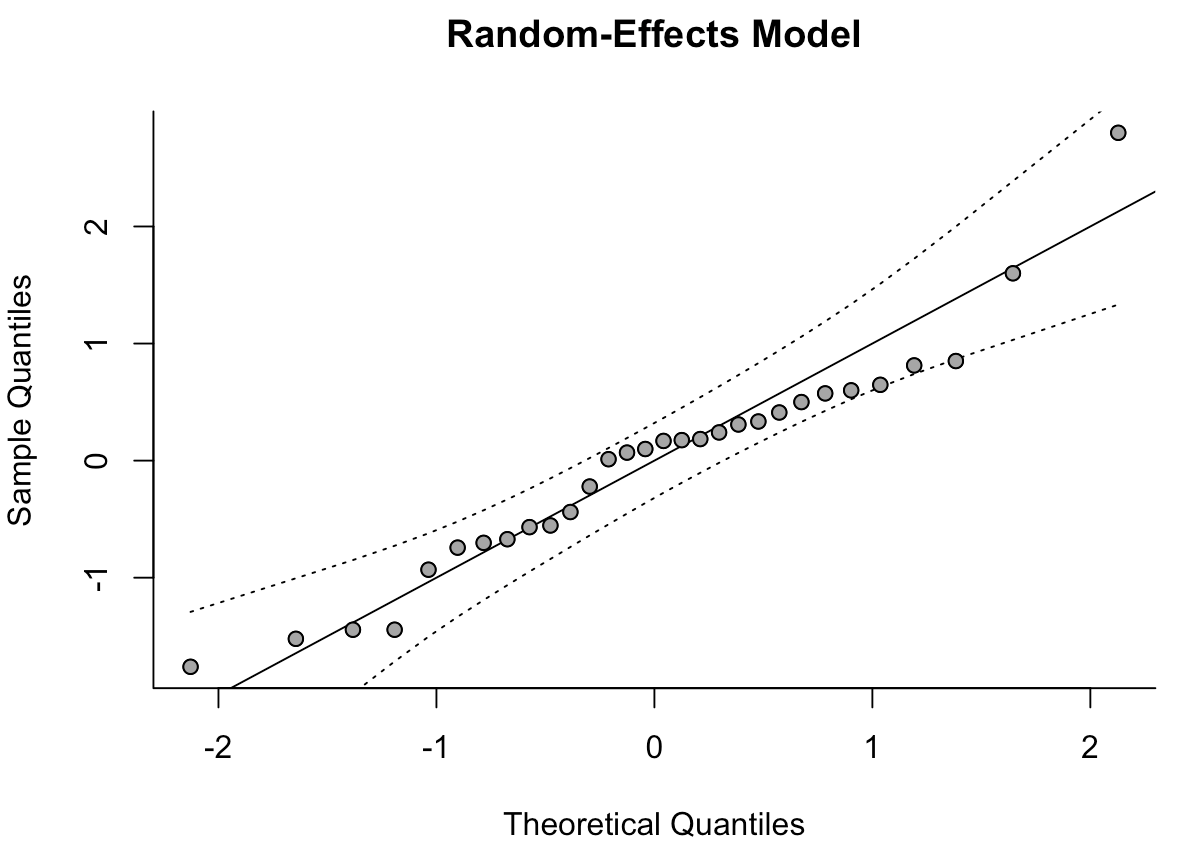


**Figure S8.** Left: Influential study analysis for the meta-analysis on pollinator species richness (N= 30 studies) using the influence() function from the *metafor* package in R. Each data point represents one study included in the meta-analysis, and red dots represent studies flagged as influential based on key influence diagnostics, such as studentised residuals, Cook’s distance, and covariance ratios metrics (Viechtbauer and Cheung 2010). **Right:** Normal Q-Q plot of residuals from the random-effects meta-analysis model. The plot compares the standardised residuals to a theoretical normal distribution. It indicates that the residuals conform to normality, suggesting that the model assumptions are appropriately met.

**Table S19.** Leave-one-out sensitivity analysis for the meta-analysis on pollinator richness (N = 30 studies). The table reports the effect size estimate (estimate), standard error (se), z-value (zval), and 95% confidence interval (ci.lb, ci.ub) after excluding each study. Heterogeneity measures include Cochran’s Q statistic (Q) and p-value (Qp), between-study variance (tau²), proportion of variation due to heterogeneity (I²), and total-to-sampling variability ratio (H²).

| **estimate** | **se** | **zval** | **pval** | **ci.lb** | **ci.ub** | **Q** | **Qp** | **tau2** | **I2** | **H2** |
| --- | --- | --- | --- | --- | --- | --- | --- | --- | --- | --- |
| -0.05 | 0.03 | -2.09 | 0.04 | -0.11 | 0.00 | 131.25 | 0.00 | 0.01 | 80.25 | 5.06 |
| -0.06 | 0.03 | -2.13 | 0.03 | -0.11 | 0.00 | 130.07 | 0.00 | 0.01 | 80.19 | 5.05 |
| -0.06 | 0.03 | -2.12 | 0.03 | -0.11 | 0.00 | 130.97 | 0.00 | 0.01 | 80.38 | 5.10 |
| -0.05 | 0.03 | -2.06 | 0.04 | -0.11 | 0.00 | 131.32 | 0.00 | 0.01 | 80.45 | 5.12 |
| -0.06 | 0.03 | -2.13 | 0.03 | -0.11 | 0.00 | 129.48 | 0.00 | 0.01 | 79.89 | 4.97 |
| -0.05 | 0.03 | -1.98 | 0.05 | -0.10 | 0.00 | 130.73 | 0.00 | 0.01 | 80.15 | 5.04 |
| -0.05 | 0.03 | -2.08 | 0.04 | -0.11 | 0.00 | 131.33 | 0.00 | 0.01 | 80.18 | 5.05 |
| -0.05 | 0.03 | -1.89 | 0.06 | -0.10 | 0.00 | 128.31 | 0.00 | 0.01 | 79.56 | 4.89 |
| -0.05 | 0.03 | -1.88 | 0.06 | -0.10 | 0.00 | 129.34 | 0.00 | 0.01 | 80.04 | 5.01 |
| -0.05 | 0.03 | -2.01 | 0.04 | -0.10 | 0.00 | 131.26 | 0.00 | 0.01 | 80.37 | 5.10 |
| -0.06 | 0.03 | -2.09 | 0.04 | -0.11 | 0.00 | 130.79 | 0.00 | 0.01 | 80.34 | 5.09 |
| -0.05 | 0.03 | -1.89 | 0.06 | -0.10 | 0.00 | 124.61 | 0.00 | 0.01 | 77.63 | 4.47 |
| -0.05 | 0.03 | -1.99 | 0.05 | -0.11 | 0.00 | 131.32 | 0.00 | 0.01 | 78.73 | 4.70 |
| -0.05 | 0.03 | -1.84 | 0.07 | -0.10 | 0.00 | 123.34 | 0.00 | 0.01 | 78.77 | 4.71 |
| -0.06 | 0.03 | -2.06 | 0.04 | -0.11 | 0.00 | 129.77 | 0.00 | 0.01 | 78.51 | 4.65 |
| -0.05 | 0.03 | -2.02 | 0.04 | -0.11 | 0.00 | 131.34 | 0.00 | 0.01 | 80.41 | 5.11 |
| -0.06 | 0.03 | -2.20 | 0.03 | -0.11 | -0.01 | 125.60 | 0.00 | 0.01 | 79.21 | 4.81 |
| -0.05 | 0.03 | -1.94 | 0.05 | -0.10 | 0.00 | 130.67 | 0.00 | 0.01 | 80.29 | 5.07 |
| -0.06 | 0.03 | -2.06 | 0.04 | -0.11 | 0.00 | 131.26 | 0.00 | 0.01 | 80.53 | 5.14 |
| -0.05 | 0.03 | -2.03 | 0.04 | -0.11 | 0.00 | 131.35 | 0.00 | 0.01 | 80.54 | 5.14 |
| -0.05 | 0.03 | -1.90 | 0.06 | -0.10 | 0.00 | 130.05 | 0.00 | 0.01 | 80.21 | 5.05 |
| -0.05 | 0.03 | -1.90 | 0.06 | -0.10 | 0.00 | 129.66 | 0.00 | 0.01 | 79.96 | 4.99 |
| -0.04 | 0.03 | -1.73 | 0.08 | -0.09 | 0.01 | 113.39 | 0.00 | 0.01 | 77.22 | 4.39 |
| -0.07 | 0.02 | -3.34 | 0.00 | -0.11 | -0.03 | 66.30 | 0.00 | 0.01 | 63.71 | 2.76 |
| -0.06 | 0.03 | -2.17 | 0.03 | -0.11 | -0.01 | 130.31 | 0.00 | 0.01 | 80.18 | 5.05 |
| -0.06 | 0.03 | -2.37 | 0.02 | -0.11 | -0.01 | 125.81 | 0.00 | 0.01 | 78.89 | 4.74 |
| -0.05 | 0.03 | -1.87 | 0.06 | -0.10 | 0.00 | 127.99 | 0.00 | 0.01 | 79.44 | 4.86 |
| -0.06 | 0.03 | -2.12 | 0.03 | -0.11 | 0.00 | 130.91 | 0.00 | 0.01 | 80.15 | 5.04 |
| -0.05 | 0.03 | -2.07 | 0.04 | -0.11 | 0.00 | 131.32 | 0.00 | 0.01 | 80.42 | 5.11 |
| -0.04 | 0.03 | -1.75 | 0.08 | -0.09 | 0.01 | 121.56 | 0.00 | 0.01 | 77.69 | 4.48 |

**Publication bias**

Egger’s regression test showed no evidence of small-study effects in the pollinator richness meta-analysis (z = -0.57, p = 0.57, 95% CI: -0.12 to 0.06), suggesting no significant publication bias.


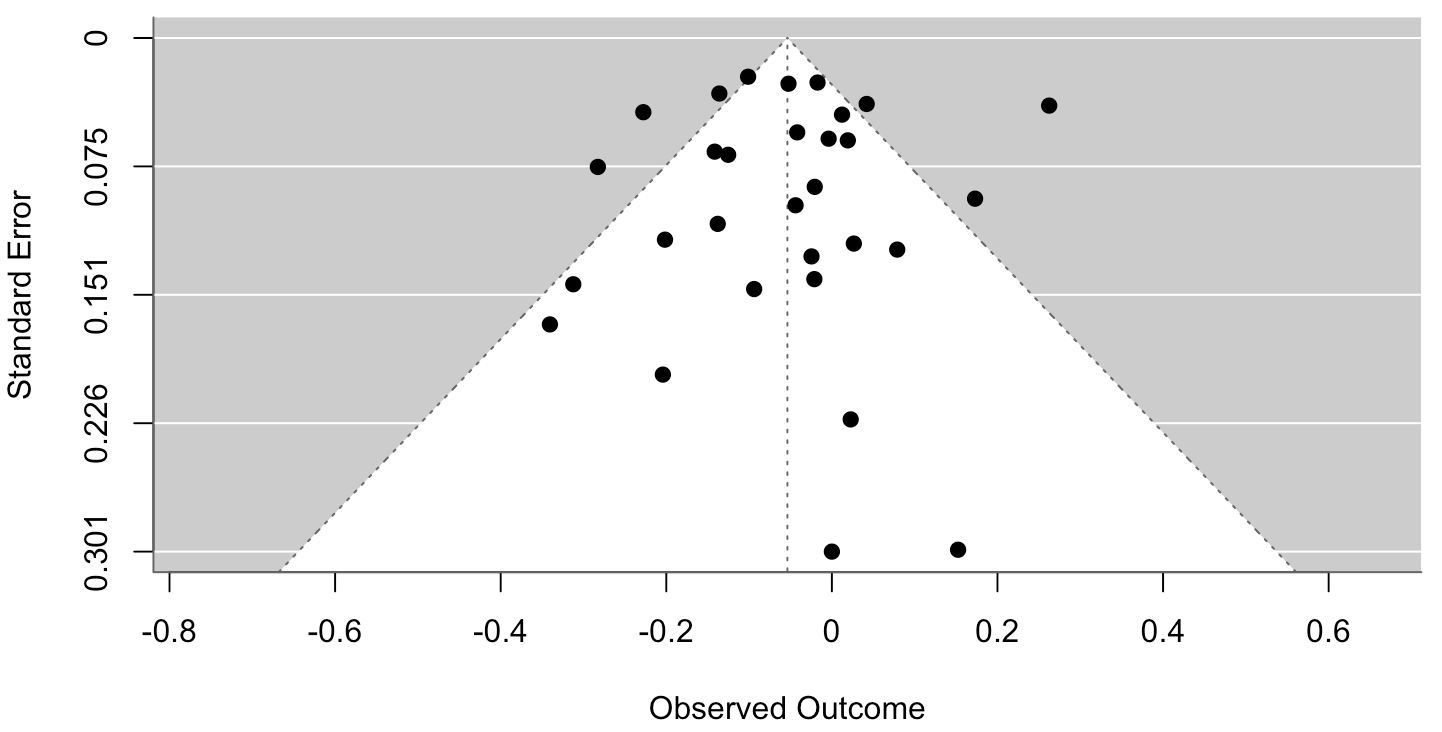


**Figure S9.** Funnel plot for the pollinator species richness meta-analysis.

## Fruit set


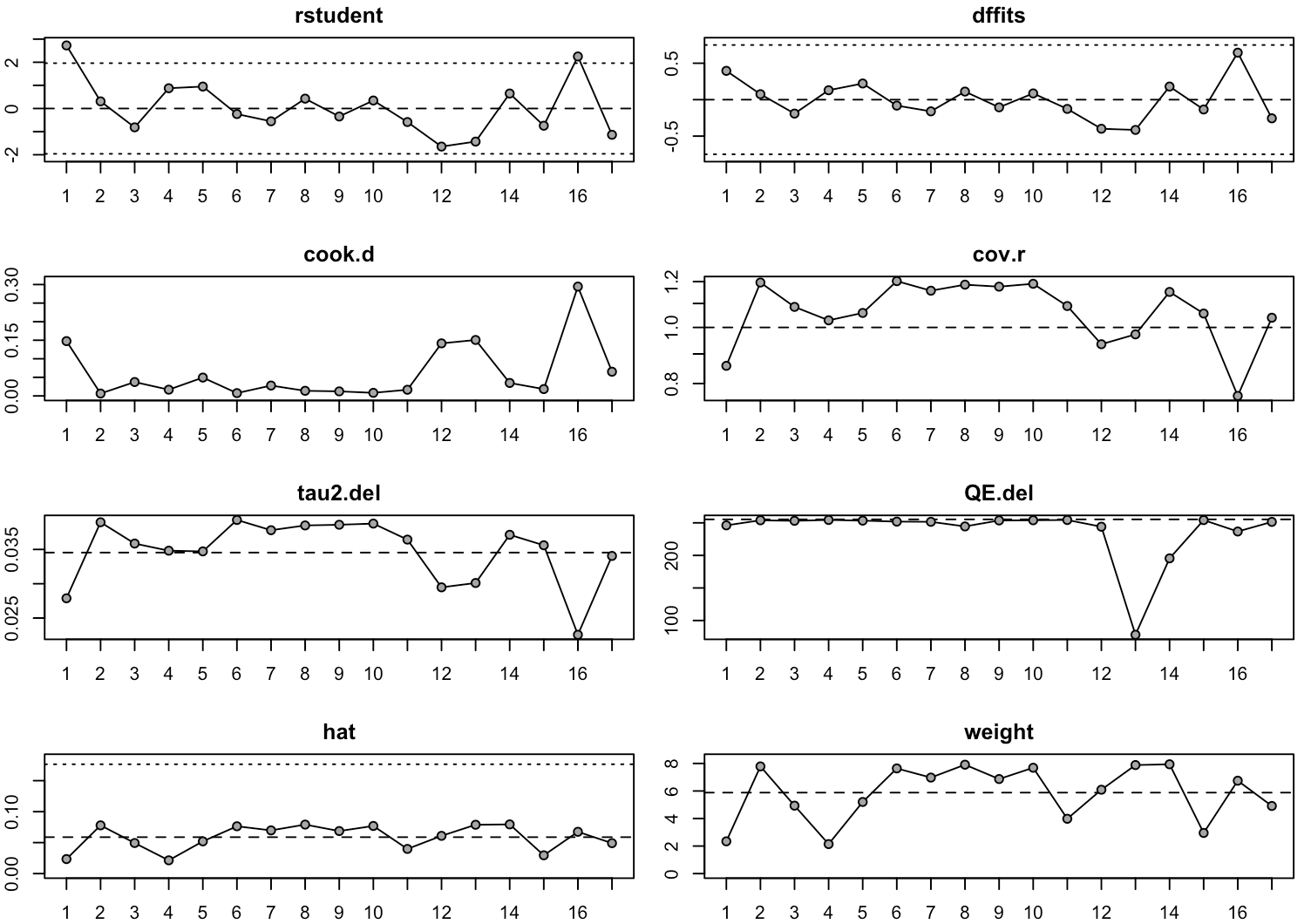

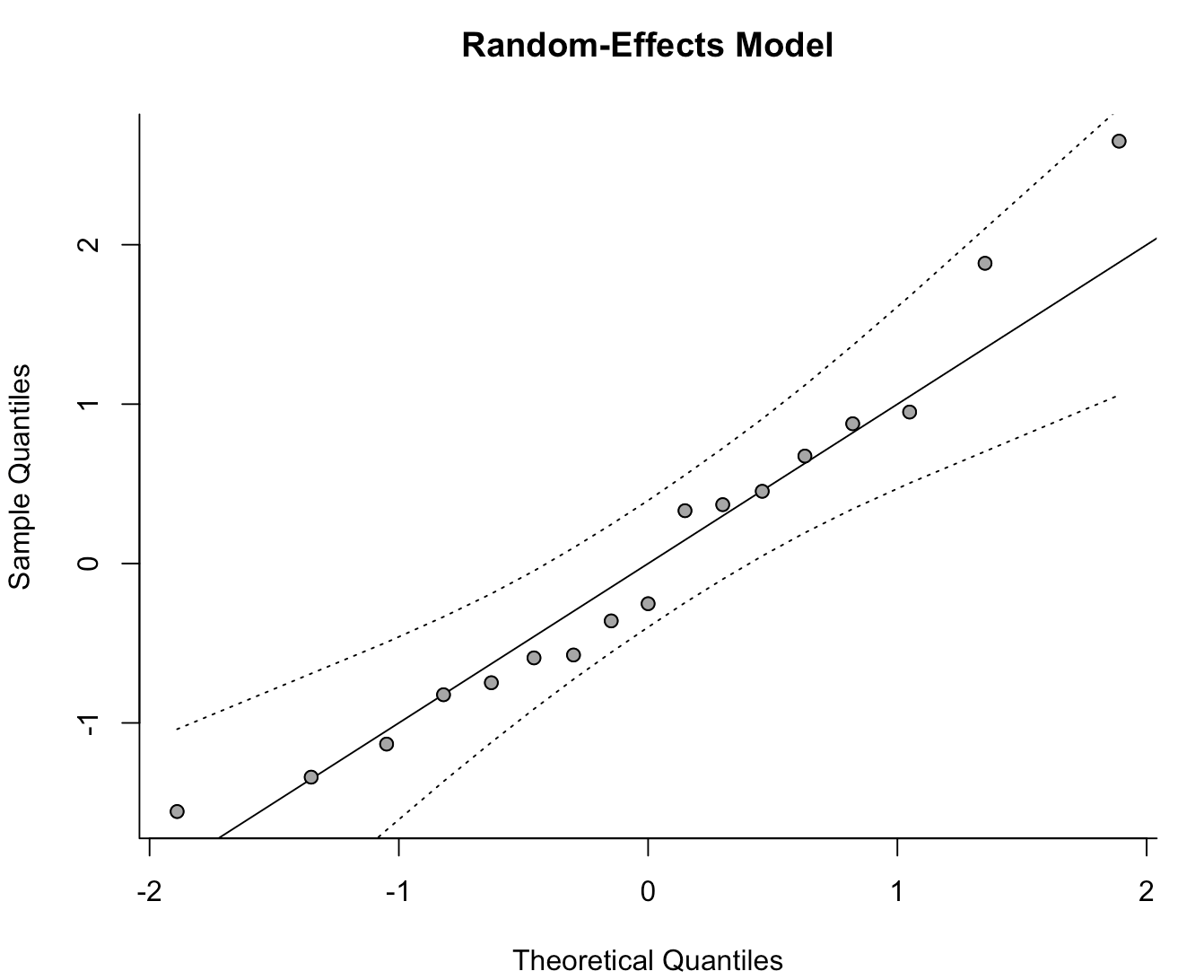


**Figure S10.** Left: Influential study analysis for the meta-analysis on fruit set (N= 17 studies) using the influence() function from the *metafor* package in R. Each data point represents one study included in the meta-analysis, and red dots represent studies flagged as influential based on key influence diagnostics, such as studentised residuals, Cook’s distance, and covariance ratios metrics (Viechtbauer and Cheung 2010). **Right:** Normal Q-Q plot of residuals from the random-effects meta-analysis model. The plot compares the standardised residuals to a theoretical normal distribution. The plot indicates that the residuals conform to normality, suggesting that the model assumptions are appropriately met.

**Table S20.** Leave-one-out sensitivity analysis for the meta-analysis on fruit set (N= 17 studies). The table reports the effect size estimate (estimate), standard error (se), z-value (zval), and 95% confidence interval (ci.lb, ci.ub) after excluding each study. Heterogeneity measures include Cochran’s Q statistic (Q) and p-value (Qp), between-study variance (tau²), proportion of variation due to heterogeneity (I²), and total-to-sampling variability ratio (H²).

| **estimate** | **se** | **zval** | **pval** | **ci.lb** | **ci.ub** | **Q** | **Qp** | **tau2** | **I2** | **H2** |
| --- | --- | --- | --- | --- | --- | --- | --- | --- | --- | --- |
| -0.09 | 0.05 | -1.81 | 0.07 | -0.18 | 0.01 | 246.14 | 0.00 | 0.03 | 94.78 | 19.16 |
| -0.07 | 0.06 | -1.25 | 0.21 | -0.18 | 0.04 | 253.91 | 0.00 | 0.04 | 95.69 | 23.20 |
| -0.06 | 0.05 | -1.05 | 0.29 | -0.16 | 0.05 | 253.13 | 0.00 | 0.04 | 95.88 | 24.25 |
| -0.07 | 0.05 | -1.40 | 0.16 | -0.18 | 0.03 | 254.44 | 0.00 | 0.03 | 95.78 | 23.67 |
| -0.08 | 0.05 | -1.47 | 0.14 | -0.19 | 0.03 | 253.40 | 0.00 | 0.03 | 95.74 | 23.49 |
| -0.06 | 0.06 | -1.10 | 0.27 | -0.18 | 0.05 | 251.92 | 0.00 | 0.04 | 95.95 | 24.71 |
| -0.06 | 0.06 | -1.04 | 0.30 | -0.17 | 0.05 | 251.59 | 0.00 | 0.04 | 96.01 | 25.05 |
| -0.07 | 0.06 | -1.29 | 0.20 | -0.19 | 0.04 | 244.52 | 0.00 | 0.04 | 94.78 | 19.16 |
| -0.06 | 0.06 | -1.09 | 0.28 | -0.17 | 0.05 | 253.67 | 0.00 | 0.04 | 96.10 | 25.62 |
| -0.07 | 0.06 | -1.27 | 0.21 | -0.18 | 0.04 | 253.97 | 0.00 | 0.04 | 95.85 | 24.09 |
| -0.06 | 0.05 | -1.11 | 0.27 | -0.17 | 0.05 | 254.39 | 0.00 | 0.04 | 95.95 | 24.67 |
| -0.05 | 0.05 | -0.94 | 0.35 | -0.15 | 0.05 | 244.07 | 0.00 | 0.03 | 95.00 | 20.00 |
| -0.05 | 0.05 | -0.91 | 0.36 | -0.15 | 0.05 | 78.14 | 0.00 | 0.03 | 93.84 | 16.24 |
| -0.08 | 0.06 | -1.38 | 0.17 | -0.19 | 0.03 | 195.45 | 0.00 | 0.04 | 94.03 | 16.74 |
| -0.06 | 0.05 | -1.12 | 0.26 | -0.17 | 0.05 | 254.23 | 0.00 | 0.04 | 95.86 | 24.17 |
| -0.10 | 0.05 | -2.10 | 0.04 | -0.19 | -0.01 | 236.72 | 0.00 | 0.02 | 93.52 | 15.44 |
| -0.05 | 0.05 | -1.01 | 0.31 | -0.16 | 0.05 | 251.47 | 0.00 | 0.03 | 95.67 | 23.08 |

**Publication bias**

Egger’s regression test showed no evidence of small-study effects in the pollinator fruit set meta-analysis (z = 0.73, p = 0.47, 95% CI: -0.28 to 0.05), suggesting no significant publication bias.


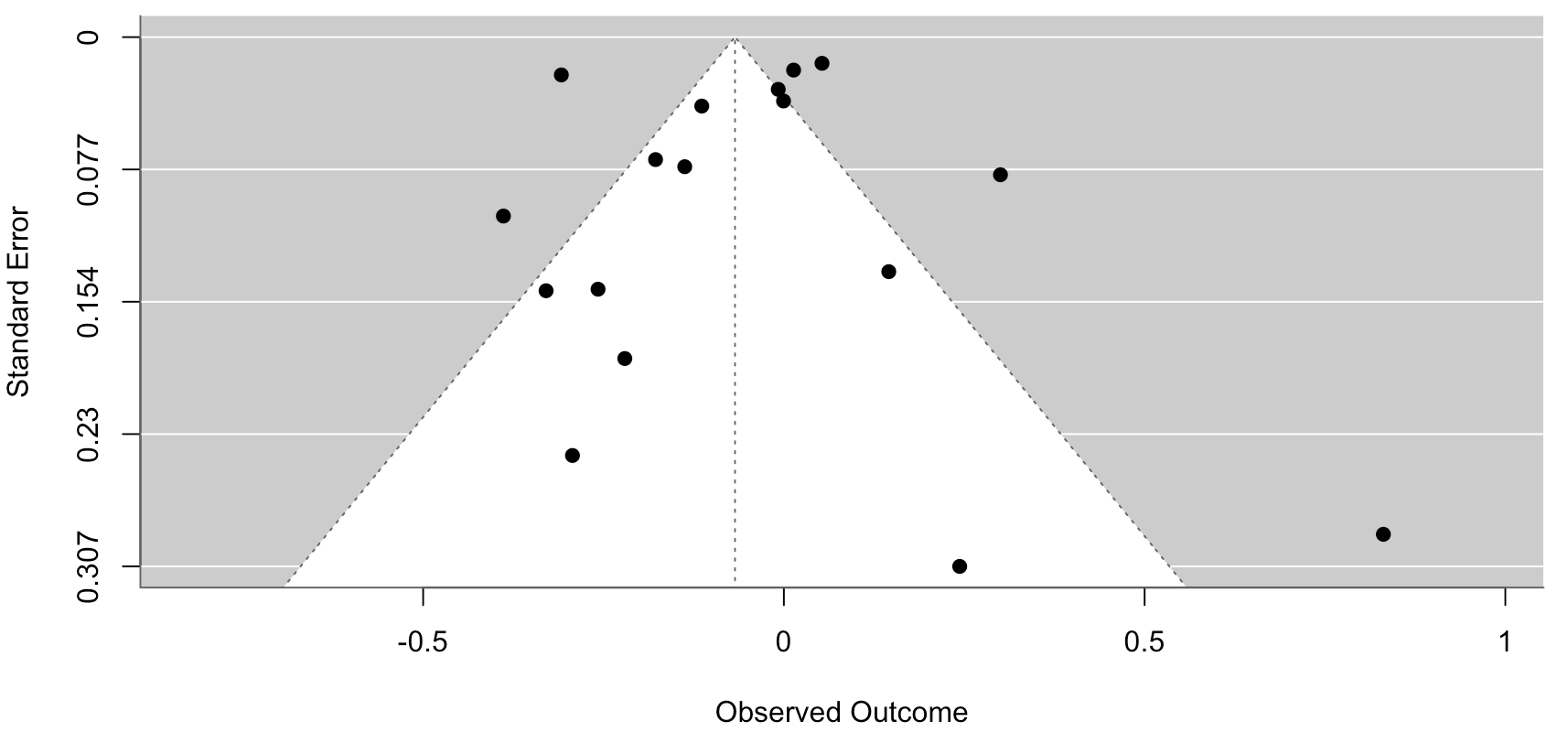


**Figure S11.** Funnel plot for the fruit set meta-analysis.

# Certainty assessment

**Table S21.** Overview of justification for GRADE assessment following the Cochrane guidelines (Schünemann et al. 2023).

| **Domains for assessing certainty of evidence by outcome** | **Examples of reasons for lowering or increasing the certainty of evidence** | | |
| --- | --- | --- | --- |
|  | **Abundance** | **Richness** | **Fruit set** |
| Risk of bias | Downgraded because of medium to high risk of bias in studies. | | |
| Inconsistency | Downgraded due  to high heterogeneity (I^2^= 74.70%). | Downgraded due to high heterogeneity (I^2^= 79.49%). | Downgraded due to high heterogeneity (I^2^= 95.48%). |
| Indirectness | Downgraded due to variability in pollinator taxa (for pollinator abundance and richness) and spatial scales of habitat isolation. Different taxa may respond differently to distance effects, and studies used varying distance ranges and definitions of natural habitat. | | |
| Imprecision | Downgraded due to wide confidence intervals, which included both positive and negative values. | Not downgraded because the confidence interval for richness remained entirely in the negative range | Downgraded due to wide confidence intervals, which included both positive and negative values. |
| Publication bias | Not downgraded because Egger’s regression test did not indicate publication bias | | |
| Large effects (upgrading) | As no established thresholds are available, upgrading based on large effects not application here. | | |
| Dose response (upgrading) | Not applicable to this question. | | |
| Opposing plausible residual bias and confounding (upgrading) | Not upgraded because the estimates of effects are not controlled for the following possible confounders: pesticide use and seasonal variability. | | |

# Funding statements for data-contributing authors

**Table S22.** Summary of the funding sources acknowledged by each data-contributing author for the original research from which they shared data for this systematic review and meta-analysis.

| **Corresponding author** | **Funding statement** |
| --- | --- |
| John E. Banks | Funded by Earthwatch Institute |
| Parthiba Basu | Funded by Darwin Initiatives, UK granted through DEFRA, Govt. of UK |
| Quebin Bosbely Casiá-Ajché | This research was co-financed by the General Research Directoriate (Dirección General de Investigación) of the University of San Carlos of Guatemala (DIGI) through the Grant B32-2020, and by the Early Career Fellowship Program, Organization for Women in Science for the Developing World (OWSD) through the Grant 45004272284 (2021–2022). |
| Aditi Dutta | funded by Darwin Initiatives, UK granted through DEFRA, Govt. of UK |
| Eunice Enríquez | Funded by Secretaría Nacional de Ciencia y Tecnología (SENACYT) of Guatemala (Grant No. 26-2012). |
| Natalia Escobedo-Kenefic | This study was supported by the University of San Carlos of Guatemala (USAC) and by the National Autonomous University of Mexico (UNAM) by providing human and logistical resources, and laboratory facilities. |
| Jaboury Ghazoul | Funded by the Swiss National Science Foundation, ETH Zurich Research Grant, and the Mercator Foundation |
| Annika L. Hass | Funded by BMBF (German Federal Ministry of Education and Research) Framework Programme Research for Sustainable Development (FONA, FKZ: 01LL0917A, 01LL0917D). |
| Olivier Honnay | Funded by VLIR-UOS |
| John Muo Kasina | This research was financially supported by the German Ministry of Education and Research (BMBF) (01LC0413) |
| Alexandra-Maria Klein | funded by the German Science Foundation (DFG) and by the project RestPoll "Restoring pollinator habitats across European agricultural landscapes based on multi-actor participatory approaches" within European Union’s Horizon Europe Framework Programme under project No. 101082101 |
| Smitha Krishnan | Funded by North South Centre, ETH Zurich Research Grant, the Mercator Foundation and the CGIAR Science Program on Multifunctional Landscapes. |
| Patricia Landaverde | PLG was funded by the German Academic Exchange Service (DAAD) |
| Kevin Li | Deutsche Forschungsgemeinschaft (DFG, German Research Foundation) – project ID 192626868 – SFB 990 |
| J. Javier G. Quezada-Euán | Funding reference: FONCICYT 94293 |
| Pornpimon Tangtorwangsakul | Funded by The National Science andTechnology Development Agency (NSTDA) |
| Manuel Toledo-Hernandez | Funded by the Consejo Nacional de Humanidades, Ciencias y Tecnologías (Conhacyt), German Academic Exchange Service (DAAD), and Agroecology Group (University of Göttingen) |
| Poornima Viswanathan | Critical Ecosystem Partnership Fund (CEPF Grant No. 62927) |
| Cassandra Vogel | 2017-2018 Belmont Forum and BiodivERsA joint call for research proposals, under the BiodivScen ERA-NetCOFUND program, and funded by the Natural Sciences and Engineering Research Council of Canada (NSERC Grant #523660-2018), National Science Foundation (NSF Grant #1852587), German Federal Education and Research (BMBF #01LC11804A) and the Research Ministry of Council of Norway (#295442). |
| Catrin Westphal | Funded by BMBF (German Federal Ministry of Education and Research) Framework Programme Research for Sustainable Development (FONA, FKZ: 01LL0917A, 01LL0917D). |

# References

Armas-Quiñonez, G., R. Ayala-Barajas, C. Avendaño-Mendoza, R. Lindig-Cisneros, and E. del-Val. 2020. Bee diversity in secondary forests and coffee plantations in a transition between foothills and highlands in the Guatemalan Pacific Coast. PEERJ **2020**.

Arnold, S. E. J., F. Elisante, P. A. Mkenda, Y. L. B. Tembo, P. A. Ndakidemi, G. M. Gurr, I. A. Darbyshire, S. R. Belmain, and P. C. Stevenson. 2021. Beneficial insects are associated with botanically rich margins with trees on small farms. Scientific Reports **11**.

Banks, J. E., L. Hannon, P. Hanson, T. Dietsch, S. Castro, N. Urena, and M. Chandler. 2013. Effects of proximity to forest habitat on hymenoptera diversity in a Costa Rican coffee agroecosystem. Pan-Pacific Entomologist **89**:60-68.

Banks, J. E., L. M. Hannon, T. V. Dietsch, and M. Chandler. 2014. Effects of seasonality and farm proximity to forest on Hymenoptera in Tarrazu coffee farms. International Journal of Biodiversity Science, Ecosystem Services and Management **10**:128-132.

Chiawo, D. O., C. K. P. O. Ogol, E. N. Kioko, V. A. Otiende, and M. W. Gikungu. 2017. Bee diversity and floral resources along a disturbance gradient in Kaya Muhaka forest and surrounding farmlands of coastal Kenya. Journal of Pollination Ecology **20**:51-59.

De Aguiar, W. M., S. H. Sofia, G. A. R. Melo, and M. C. Gaglianone. 2015. Changes in Orchid Bee Communities Across Forest-Agroecosystem Boundaries in Brazilian Atlantic Forest Landscapes. ENVIRONMENTAL ENTOMOLOGY **44**:1465-1471.

de Sousa, F. G., J. S. dos Santos, F. Martello, M. F. Diniz, L. L. Bergamini, M. C. Ribeiro, R. G. Collevatti, and D. P. Silva. 2022. Natural habitat cover and fragmentation per se influence orchid-bee species richness in agricultural landscapes in the Brazilian Cerrado. APIDOLOGIE **53**.

Escobedo-Kenefic, N., E. Cardona, M. d. C. Arizmendi, and C. A. Domínguez. 2024. Do forest reserves help maintain pollinator diversity and pollination services in tropical agricultural highlands? A case study using Brassica rapa as a model. Frontiers in Bee Science **2**.

Escobedo-Kenefic, N., Q. B. Casiá-Ajché, E. Cardona, D. Escobar-González, A. Mejía-Coroy, E. Enríquez, and P. Landaverde-González. 2022. Landscape or local? Distinct responses of flower visitor diversity and interaction networks to different land use scales in agricultural tropical highlands. Frontiers in Sustainable Food Systems **6**.

FAO. 2017. Defining small-scale food producers to monitor target 2.3. of the 2030 Agenda for Sustainable Development. Rome.

Ferreira, J. V. A., V. Arroyo-Rodríguez, J. C. Morante, D. Storck-Tonon, A. Somavilla, J. A. dos Santos-Silva, T. Mahlmann, M. L. Oliveira, and M. Benchimol. 2024. Landscape forest cover and regional context shape the conservation value of shaded cocoa agroforests for bees and social wasps. LANDSCAPE ECOLOGY **39**.

Geeraert, L., R. Aerts, G. Berecha, G. Daba, N. De Fruyt, J. D'Hollander, K. Helsen, H. Stynen, and O. Honnay. 2020. Effects of landscape composition on bee communities and coffee pollination in Coffea arabica production forests in southwestern Ethiopia. Agriculture, Ecosystems and Environment **288**.

Gemmill-Herren, B., and A. O. Ochieng. 2008. Role of native bees and natural habitats in eggplant (Solanum melongena) pollination in Kenya. Agriculture, Ecosystems & Environment **127**:31-36.

Gikungu, M., D. Wittmann, D. Irungu, and M. Kraemer. 2011. Bee diversity along a forest regeneration gradient in Western Kenya. Journal of Apicultural Research **50**:22-34.

Gonzalez, E., A. Salvo, and G. Valladares. 2017. Natural vegetation cover in the landscape and edge effects: differential responses of insect orders in a fragmented forest. Insect Science **24**:891-901.

González-Chaves, A. D., L. Gigante Carvalheiro, P. Ribero Piffer, F. d’Albertas, T. C. Giannini, B. F. Viana, and J. P. Metzger. 2023. Evidence of time-lag in the provision of ecosystem services by tropical regenerating forests to coffee yields. ENVIRONMENTAL RESEARCH LETTERS **18**:025002.

Hagen, M., M. Kraemer, and S. Boutin. 2010. Agricultural surroundings support flower-visitor networks in an Afrotropical rain forest. Special Section: Conservation planning within emerging global climate and economic realities. **143**:1654-1663.

Jeronimo, F., and I. G. Varassin. 2023. Like an “espresso” but not like a “cappuccino”: landscape metrics are useful for predicting coffee production at the farm level but not at the municipality level. Environmental Monitoring and Assessment **195**.

Klein, A. M., J. Tylianakis, I. Steffan-Dewenter, T. Tscharntke, K. H. Hartfelder, D. d. Jong, R. A. Pereira, A. d. Santos Cristino, M. M. Morais, E. D. Tanaka, A. P. Lourenco, J. E. B. d. Silva, G. F. d. Almeida, and A. M. d. Nascimento. 2004. Landscape context affects bee diversity and pollination in European and tropical agroecosystems. Proceedings of the 8th IBRA International Conference on Tropical Bees and VI Encontro sobre Abelhas, Ribeirao Preto, Brasil, 6-10 September, 2004:121-126.

Konno, K., B. Livoreil, and A. S. Pullin. 2021. Collaboration for Environmental Evidence Critical Appraisal Tool Version 0.3 (Prototype).

Laha, S., S. Chatterjee, A. Das, B. Smith, and P. Basu. 2020. Exploring the importance of floral resources and functional trait compatibility for maintaining bee fauna in tropical agricultural landscapes. JOURNAL OF INSECT CONSERVATION **24**:431-443.

Landaverde-Gonzalez, P., J. J. G. Quezada-Euan, P. Theodorou, T. E. Murray, M. Husemann, R. Ayala, H. Moo-Valle, R. Vandame, and R. J. Paxton. 2017. Sweat bees on hot chillies: provision of pollination services by native bees in traditional slash-and-burn agriculture in the Yucatan Peninsula of tropical Mexico. Journal of Applied Ecology **54**:1814-1824.

Lasway, J. V., M. K. Peters, H. K. Njovu, C. Eardley, A. Pauly, and I. Steffan-Dewenter. 2022. Agricultural intensification with seasonal fallow land promotes high bee diversity in Afrotropical drylands. Journal of Applied Ecology **59**:3014-3026.

Li, K., I. Grass, T.-Y. Fung, R. Fardiansah, M. Rohlfs, D. Buchori, and T. Tscharntke. 2022. Adjacent forest moderates insect pollination of oil palm. Agriculture, Ecosystems & Environment **338**.

Lima, M. M., and E. Mariano Neto. 2014. Extinction thresholds for Sapotaceae due to forest cover in Atlantic Forest landscapes. Forest Ecology and Management **312**:260-270.

Livingston, G., S. Jha, A. Vega, and L. Gilbert. 2013. Conservation value and permeability of neotropical oil palm landscapes for orchid bees. PLOS ONE **8**:e78523.

Lowder, S. K., M. V. Sánchez, and R. Bertini. 2021. Which farms feed the world and has farmland become more concentrated? World Development **142**:105455.

Nery, L. S., J. T. Takata, B. B. Camargo, A. M. Chaves, P. A. Ferreira, and D. Boscolo. 2018. Bee diversity responses to forest and open areas in heterogeneous Atlantic Forest. SOCIOBIOLOGY **65**:686-695.

Obregon, D., O. Guerrero, E. Stashenko, and K. Poveda. 2021. Natural habitat partially mitigates negative pesticide effects on tropical pollinator communities. Global Ecology and Conservation **28**:e01668.

Olson, D. M., J. Gibbs, and J. M. Schmidt. 2021. Wild Bee Pollinators Foraging in Peanut and Cotton Adjacent to Native Wildflower Strips. Florida Entomologist **104**:165-172.

Outhwaite, C. L., A. M. D. Ortiz, F. E. B. Spooner, C. Dalin, and T. Newbold. 2022. Availability and proximity of natural habitat influence cropland biodiversity in forest biomes globally. GLOBAL ECOLOGY AND BIOGEOGRAPHY **31**:1589-1602.

Page, M. J., J. E. McKenzie, P. M. Bossuyt, I. Boutron, T. C. Hoffmann, C. D. Mulrow, L. Shamseer, J. M. Tetzlaff, E. A. Akl, S. E. Brennan, R. Chou, J. Glanville, J. M. Grimshaw, A. Hróbjartsson, M. M. Lalu, T. Li, E. W. Loder, E. Mayo-Wilson, S. McDonald, L. A. McGuinness, L. A. Stewart, J. Thomas, A. C. Tricco, V. A. Welch, P. Whiting, and D. Moher. 2021. The PRISMA 2020 statement: an updated guideline for reporting systematic reviews. BMJ **372**:n71.

Priess, J. A., M. Mimler, A. M. Klein, S. Schwarze, T. Tscharntke, and I. Steffan-Dewenter. 2007. Linking deforestation scenarios to pollination services and economic returns in coffee agroforestry systems. Ecol Appl **17**:407-417.

Rethlefsen, M. L., A. M. Farrell, L. C. Osterhaus Trzasko, and T. J. Brigham. 2015. Librarian co-authors correlated with higher quality reported search strategies in general internal medicine systematic reviews. J Clin Epidemiol **68**:617-626.

Rosa, J. F., M. Ramalho, D. Monteiro, and M. D. E. Silva. 2015. Permeability of matrices of agricultural crops to Euglossina bees (Hymenoptera, Apidae) in the Atlantic Rain Forest. APIDOLOGIE **46**:691-702.

Schrader, J., M. Franzen, C. Sattler, P. Ferderer, and C. Westphal. 2018. Woody habitats promote pollinators and complexity of plant-pollinator interactions in homegardens located in rice terraces of the Philippine Cordilleras. Paddy and Water Environment. Special Issue: Rice ecosystem services. **16**:253-263.

Schüepp, C., S. Rittiner, and M. Entling. 2012. High Bee and Wasp Diversity in a Heterogeneous Tropical Farming System Compared to Protected Forest. PLOS ONE **7**:e52109.

Schünemann, H., J. Higgins, G. Vist, P. Glasziou, E. Akl, N. Skoetz, and G. Guyatt. 2023. Chapter 14: Completing ‘Summary of findings’ tables and grading the certainty of the evidence [last updated August 2023].*in* T. J. Higgins JPT, Chandler J, Cumpston M, Li T, Page MJ, Welch VA (editors), editor. Cochrane Handbook for Systematic Reviews of Interventions version 6.5. Cochrane, 2024. Available from <www.training.cochrane.org/handbook>.

Serralta-Batun, L. P., J. J. Jiménez-Osornio, V. Meléndez-Ramírez, and M. A. Munguía-Rosas. 2024. Taxonomic and Functional Diversity of Bees in Traditional Agroecosystems and Tropical Forest Patches on the Yucatan Peninsula. TROPICAL CONSERVATION SCIENCE **17**.

Sritongchuay, T., A. C. Hughes, J. Memmott, and S. Bumrungsri. 2019. Forest proximity and lowland mosaic increase robustness of tropical pollination networks in mixed fruit orchards. Landscape and Urban Planning **192**:103646.

Steffan-Dewenter, I., U. Münzenberg, C. Bürger, C. Thies, and T. Tscharntke. 2002. Scale-dependent effects of landscape context on three pollinator guilds. Ecology **83**:1421-1432.

Tangtorwongsakul, P., N. Warrit, and G. A. Gale. 2018. Effects of landscape cover and local habitat characteristics on visiting bees in tropical orchards. Agricultural and Forest Entomology. **20**:28-40.

Vansynghel, J., C. Ocampo-Ariza, B. Maas, E. A. Martin, E. Thomas, T. Hanf-Dressler, N.-C. Schumacher, C. Ulloque-Samatelo, T. Tscharntke, and I. Steffan-Dewenter. 2022. Cacao flower visitation: lowpollen deposition, low fruit set and dominance of herbivores. Ecological Solutions and Evidence **3**.

Vides-Borrell, E., L. Porter-Bolland, B. G. Ferguson, P. Gasselin, R. Vaca, J. Valle-Mora, and R. Vandame. 2019. Polycultures, pastures and monocultures: effects of land use intensity on wild bee diversity in tropical landscapes of southeastern Mexico. Biological Conservation **236**:269-280.

Viechtbauer, W., and M. W.-L. Cheung. 2010. Outlier and influence diagnostics for meta-analysis. Research Synthesis Methods **1**:112-125.

Vogel, C., T. L. Chunga, X.-x. Sun, K. Poveda, and I. Steffan-Dewenter. 2021. Higher bee abundance, but not pest abundance, in landscapes with more agriculture on a late-flowering legume crop in tropical smallholder farms. PEERJ **9**.

Vogel, C., T. Mkandawire, M. Mkandawire, G. Küstner, L. Dakishoni, R. Bezner Kerr, A. Iverson, K. Poveda, and I. Steffan-Dewenter. 2023. The importance of shrubland and local agroecological practices for pumpkin production in sub-Saharan smallholdings. Basic and Applied Ecology **73**:51-61.

Widhiono, I., E. Sudiana, and E. T. Sucianto. 2016. Insect pollinator diversity along a habitat quality gradient on Mount Slamet, Central Java, Indonesia. Biodiversitas: Journal of Biological Diversity **17**:746-752.

Willroth, E. C., and O. E. Atherton. 2024. Best Laid Plans: A Guide to Reporting Preregistration Deviations. Advances in Methods and Practices in Psychological Science **7**:25152459231213802.

Young, A. M. 1986. Habitat differences in cocoa tree flowering, fruit-set, and pollinator availability in Costa Rica. JOURNAL OF TROPICAL ECOLOGY **2**:163-186.
